# The optimal blood glucose is significantly associated with lower mortality in critically ill patients with cardiogenic shock: an analysis revealed with time series blood glucose records

**DOI:** 10.1186/s40001-024-01724-8

**Published:** 2024-02-17

**Authors:** Ce Sun, Ji-Hong Zhou, Yan-Ling Huang, Yi-Le Ning, Xiang-Hui Xu

**Affiliations:** 1 Department of Critical Care Medicine, Meizhou Hospital of Guangzhou University of Chinese Medicine, Meizhou, China; 2 Guangzhou University of Chinese Medicine, Guangzhou, China; 3 Department of Pulmonary and Critical Care Medicine (PCCM), Bao’an District Hospital of Chinese Medicine, Shenzhen, China; 4 The First Clinical School, Guangzhou University of Chinese Medicine, Guangzhou, China; 5 Department of Critical Care Medicine, Bao’an District Hospital of Chinese Medicine, Shenzhen, China

**Keywords:** Cardiogenic shock, Time-weighted average, Blood glucose, Intensive care unit, Critical care

## Abstract

**Background:**

The optimal blood glucose (BG) level for patients with cardiogenic shock in the intensive care unit (ICU) remains unclear. Studies have found that both excessively high and low BG levels contribute to adverse cardiovascular events. Our study aims to investigate the optimal BG level for critically ill patients with cardiogenic shock and evaluate the effects of optimal BG on the prognosis of patients.

**Methods:**

A total of 2013 patients with cardiogenic shock obtained from the Medical Information Mart for Intensive Care (MIMIC) IV database were included in the final cohort for our retrospective observational study for data analysis. The exposure was time-weighted average BG (TWA-BG), which was calculated by the time-series BG records and corresponding time stamps of patients with cardiogenic shock during their stay in the ICU. The cut-off value of TWA-BG was identified by the restricted cubic spline curve and included patients were categorized into three groups: low TWA-BG group (TWA-BG ≤ 104 mg/dl), optimal TWA-BG group (104 < TWA-BG ≤ 138 mg/dl), and high TWA-BG group (TWA-BG > 138 mg/dl). The primary outcome was 28-day mortality, and the secondary outcomes were ICU and in-hospital mortality. We performed the log-rank test to detect whether there is a difference in mortality among different groups in the original cohort. Multiple distinct models were employed to validate the robustness of the results.

**Results:**

Our study revealed that the optimal BG level for critically ill patients with cardiogenic shock is 104–138 mg/dl. Compared to the optimal TWA-BG group, the low TWA-BG group (hazard ratio (HR): 1.67, 95% confidence interval (CI): 1.19–2.33, *p* = 0.002) and high TWA-BG group (HR: 1.72, 95% CI: 1.46–2.03, *p* < 0.001) exhibited higher 28-day mortality. Similarly, the low TWA-BG group and high TWA-BG group demonstrated higher risks in terms of ICU mortality (low TWA-BG group: HR: 2.30, 95% CI: 1.40–3.79, *p* < 0.001; high TWA-BG group: HR: 1.77, 95% CI: 1.45–2.17, *p* < 0.001) and in-hospital mortality (low TWA-BG group: HR: 1.73, 95% CI: 1.19–2.51, *p* = 0.001; high TWA-BG group: HR: 1.64, 95% CI: 1.38–1.95, *p* < 0.001). Sensitivity analysis conducted through propensity score matching and the subgroup analysis further substantiated the robustness of the results.

**Conclusion:**

The optimal BG level for patients with cardiogenic shock is 104–138 mg/dl. BG levels below 104 mg/dl and above 138 mg/dl were associated with a less favorable prognosis.

**Supplementary Information:**

The online version contains supplementary material available at 10.1186/s40001-024-01724-8.

## Background

Cardiogenic shock presents a high mortality rate ranging from 27 to 51% in hospitals [1], highlighting the significance of optimizing patient management in this condition. While current guidelines primarily focus on hemodynamic treatment and primary pathologies, comprehensive recommendations regarding glycemic control are relatively limited [2]. Managing blood glucose (BG) levels poses a challenge in the treatment of cardiogenic shock, given the presence of stress states, disruptions in glucose metabolism, and other factors that contribute to significant fluctuations in BG levels. These fluctuations may harm patient outcomes [3]. Therefore, the effective control of BG is an imperative topic that warrants discussion in the treatment process of patients with cardiogenic shock.

Existing research has already explored the correlation between hyperglycemia and adverse outcomes in acute myocardial infarction and cardiogenic shock [4–7]. It has been proved that the BG level on admission can serve as a prognostic indicator for patients diagnosed with cardiogenic shock. Patients with admission BG levels exceeding 7.8 mmol/L exhibit elevated mortality rates during their hospital stay, as well as within one year and five years post-admission [8]. Similarly, a retrospective study revealed that individuals experiencing acute myocardial infarction and exhibiting a random BG maximum value surpassing 140 mg/dl face a heightened risk of developing cardiogenic shock [9]. Additionally, a study involving 3078 patients revealed an association between the highest in-hospital BG levels ≥ 11.5 mmol/L and increased mortality in patients with acute coronary syndrome. All these findings underscore the potential hazards of hyperglycemia in individuals with cardiogenic shock [10].

The detrimental effects of hypoglycemia on cardiovascular diseases are also gradually receiving attention. Researchers found in a nationwide population-based cohort study of over 1.5 million individuals in South Korea that experiencing severe hypoglycemic events can lead to an increase in adverse cardiovascular events and all-cause mortality. This risk escalates with the frequency of hypoglycemic episodes [11]. Subsequently, the same team of researchers discovered that for patients with acute heart failure, hypoglycemic events (BG < 3.9 mmol/L) increase the risk of major adverse cardiovascular events and all-cause mortality [12].

The studies mentioned above predominantly focused on a single random BG record or the maximum BG level during specific periods, thereby neglecting an examination of the dynamic fluctuations in BG levels. Patients in the intensive care unit (ICU) often exhibit a high degree of variability in their BG due to their changing pathophysiological state [13]. Assessing overall BG levels based solely on a single measurement is inadequate. Hence, we contend that utilizing dynamic recordings of BG data is appropriate for evaluating the overall level of glycemic control in patients with cardiogenic shock during their ICU stay. While previous studies have examined the impact of BG on the prognosis of patients with cardiogenic shock [14, 15], they have not provided sufficient evidence to delineate the optimal range for BG control. In view of both excessively elevated and excessively diminished BG levels can give rise to unfavorable outcomes, we hypothesize the existence of an optimal level for glycemic control in patients with cardiogenic shock. However, existing guidelines for intensive glycemic control do not provide specific recommendations for BG management in this population, and there is a lack of high-quality clinical evidence regarding BG management in conditions such as cardiac arrest and related diseases.

Our research aims to explore the optimal level of BG control in patients with cardiogenic shock by analyzing time series BG records. By comparing the mortality of patients within and outside this range, we aim to provide a rational target for optimal BG control.

## Methods

The Medical Information Mart for Intensive Care IV (MIMIC-IV) database version 2.0 was used for our retrospective observational study, in compliance with the REporting of studies Conducted using Observational Routinely collected health Data (RECORD) declaration. Data gathered from patients admitted to Beth Israel Deaconess Medical Center's intensive care units between 2008 and 2019 are included in the MIMIC-IV database. Yi-Le Ning, a member of our team, has been granted authorization to access the MIMIC-IV database (Record ID 40974208). The Beth Israel Deaconess Medical Center's Institutional Review Board (IRB #2001P001699) approved a waiver of informed consent because the patient information had been de-identified.

### Study population

The International Classification of Disease (ICD) codes were used to identify adult patients with cardiogenic shock. Patients who were taken out of the ICU in less than 24 h were not included. The components of the time series data for BG were all the BG records and the related record time stamps of the patients during their stay in the ICU, which we retrieved. We intended to align the BG records into 1-h resolution time series data by imputation with Stineman interpolation algorithm [16]. We therefore removed patients whose total counts of BG records were fewer than 3 in order to facilitate the interpolation using the previously proposed algorithm. Furthermore, we verified that patients maintain a minimum of one BG record daily. Here are the specific exclusion standards that were established for this retrospective observational study: (1) ICU stay less than 24 h; (2) non-adult patients younger than 18; and (3) no BG is recorded on any given day of the ICU stay, or fewer than 3 BG records in total.

### Data extraction

In order to obtain all the patient data necessary for the study, we collected all BG records, demographic information, study outcomes, laboratory results, vital signs, and scoring systems like the SAPS II and SOFA score, within the first 24 h of ICU admission. Structured query language (SQL) codes were developed and tested using DBeaver Community version 23.1.2. The DBI package was then used to execute the codes, creating the relevant tables in the MIMIC-IV database and related variables in the R global environment for data collection.

### Exposure and outcomes

We measured total BG control during an ICU stay using the time-weighted average BG (TWA-BG). This was achieved by calculating the area under the dynamic BG-time curve and dividing the result by the total ICU stay duration. The nonlinear relationship between TWA-BG and 28-day mortality was examined using a restricted cubic spline (RCS) curve based on the Cox regression model using rms package [17], determining the optimal threshold for patient grouping as the exposure factor. In the present investigation, the primary outcome was set as 28-day mortality, with the secondary outcome being mortality specifically within the confines of the ICU and hospital.

### Covariates

In the current study, a comprehensive set of 38 variables, clustered into 5 distinct categories, served as potential confounders. These categories encompassed demographic and admission data (e.g., age, sex, weight, SAPS II, SOFA score, Charlson comorbidity index), therapeutic interventions [e.g., coronary artery bypass grafting (CABG), percutaneous coronary intervention (PCI), intra-aortic balloon pump (IABP), pulse index continuous cardiac output (PiCCO), non-invasive cardiac output monitoring (NICOM), mechanical ventilation, sedative therapy], pre-existing comorbid conditions [e.g., heart failure (HF), hypertension, atrial fibrillation (AFIB), type 2 diabetes mellitus (T2DM), chronic renal disease, liver disease, chronic obstructive pulmonary disease (COPD), coronary artery disease (CAD), stroke, malignancy, cardiomyopathy, heart valvular disease (HVD)], vital signs [e.g., mean arterial pressure (MAP), temperature, heart rate], along with laboratory tests (e.g., white blood cell (WBC) count, hemoglobin, platelet count, potential of hydrogen (pH), partial pressure of oxygen (PO2), partial pressure of carbon dioxide (PCO2), lactate, creatinine, cardiac output (CO), central venous pressure (CVP)].

### Statistical analysis

We executed the Shapiro–Wilk normality test to gauge the normal distribution of the data. Bartlett's examination (for the original cohort) or the *F*-test (for the pairwise cohort) was applied to assess the equality of variances. Given the circumstances where the data exhibited normal distribution across groups and the homogeneity of variance test revealed no statistical difference, we conducted one-way ANOVA (for the original cohort) or the *t*-test (for the pairwise cohort) for continuous covariates. In contrast, if such conditions were not met, the Kruskal–Wallis test (for the original cohort) or the Wilcoxon test (for the pairwise cohort) was deemed suitable. The Chi-square test was utilized for categorical covariates, while Fisher's exact test was used if the sample size for any cell was less than 10. Continuous variables were articulated as mean (standard deviation), while categorical variables were conveyed as numerical values (percentage).

We employed both pairwise propensity score matching (PSM) and inverse probability of treatment weighting (IPTW) based on the propensity score to adjust for covariates, thereby fortifying the robustness of our results. The Matching package was utilized to conduct pairwise PSM, resulting in the creation of two matched cohorts (Cohort 1: optimal TWA-BG group versus low TWA-BG group, and Cohort 2: optimal TWA-BG group versus high TWA-BG group). The propensity score, generated with the logistic regression model, was used as the basis for further propensity score-based analysis. We 1:1 matched cohorts with the Match function in Matching package based on their propensity scores with a caliper width equal to 0.2 of the standard deviation of the logit of the propensity score [18–20]. Whether the absolute values of standardized mean difference (SMD) of all covariates between groups exceeded the threshold of 0.1 was used to assess the balance of covariates. Multiple imputations were performed with the mice package for these two pairwise cohorts before pairwise PSM. Variables with missing values exceeding 40% will not be included as covariates in the model for analysis [21]. The unadjusted log-rank test was employed with the survival package [22] to estimate the original cohort.

To ensure the robustness of the results, we applied a series of models, such as sensitivity analysis for 28-day, ICU, and in-hospital mortality, which included the multivariate Cox model adjusted with all covariates, multivariate Cox model adjusted with unbalanced covariates, multivariate Cox model adjusted with all covariates using IPTW, and doubly robust estimation (survey-weighted Cox model) with all covariates using IPTW. The assumption of proportional hazards was tested via the analysis of Schoenfeld residuals. In the event that the assumption of proportional hazards was statistically significant, time-changing covariates with time-transform features were incorporated with the tt function into the Cox regression model or survey-weighted Cox model.

All statistical approaches were deployed with R version 4.2.3. The threshold of statistical significance is established at *p* < 0.05.

## Results

### Baseline characters and grouping

**Fig. 1:**
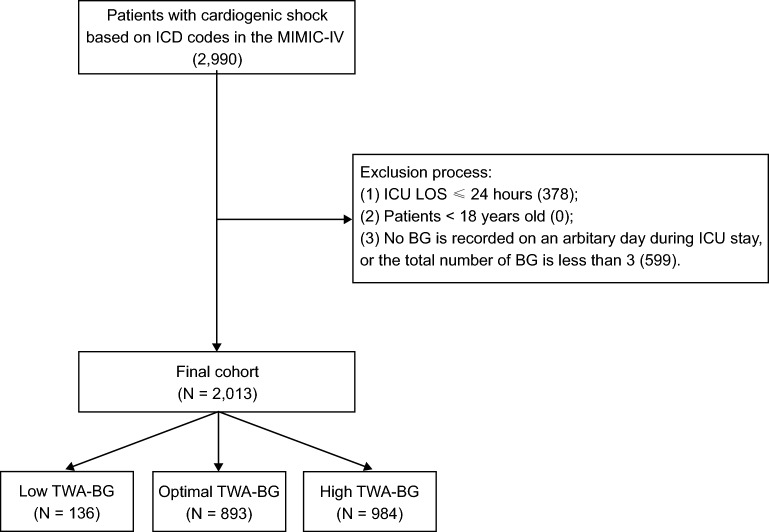
The study flowchart outlines the inclusion and exclusion criteria utilized to select the final cohort of 2013 patients

**Fig. 2:**
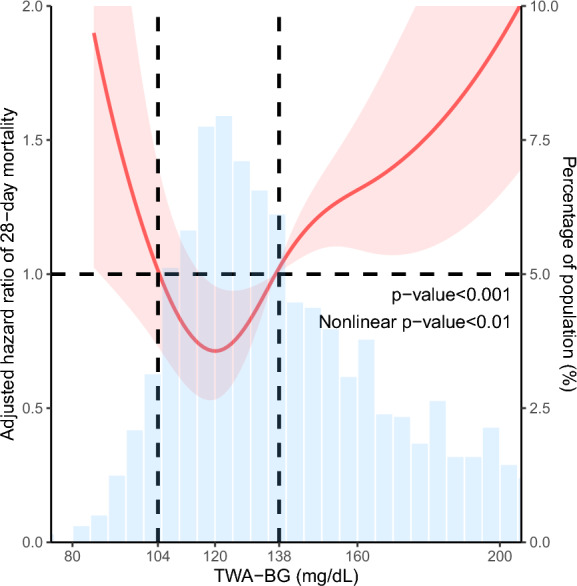
The nonlinear relationship of TWA-BG and the risk of 28-day mortality fit by multivariate Cox regression with RCS analysis

We initially identified a total of 2990 patients with cardiogenic shock using ICD codes in the database. After excluding 977 patients, a final cohort of 2013 patients was included in the data analysis (Fig. 1). To investigate the optimal cut-off value of TWA-BG for 28-day mortality, we performed the RCS curve based on the Cox regression model (Fig. 2), which indicated a nonlinear relationship between TWA-BG and the 28-day mortality in patients with cardiogenic shock (*p* < 0.001, nonlinear *p* < 0.01). Thus, we identified an optimal TWA-BG range of 104 to 138 mg/dL. BG levels exceeding 138 mg/dL and falling below 104 mg/dL were associated with an increased risk of 28-day mortality. Based on these findings, we categorized the patients into 3 groups according to the TWA-BG levels: low TWA-BG group (TWA-BG ≤ 104 mg/dL, *n* = 136), optimal TWA-BG group (104 mg/dL < TWA-BG ≤ 138 mg/dL, *n* = 893), and high TWA-BG group (TWA-BG > 138 mg/dL, *n* = 984). The frequency distribution of TWA-BG data of all included patients can be found in Additional file 1, which revealed that the lowest TWA-BG recorded was 69 mg/dl, with a relatively fewer proportion of patients below 104 mg/dl. And the range distribution of TWA-BG was showed in Additional file 2. Interestingly, we observed a smaller number of patients in the low TWA-BG group compared to the optimal and high TWA-BG groups. This may be attributable to a clinical preference among ICU medical staff to maintain patients' BG levels within a safer (namely higher) cushion range. We simultaneously analyzed the first 24 h, 48 h, 72 h, and 7 days TWA-BG trend for each group. It can be observed that the trend in TWA-BG aligns with the characteristic BG distributions for each group (Additional file 3: Fig. S1).

In the study population, the overall mean age was 68.24 ± 14.32 years. There were 733 female cases, constituting 36.41% of the total. The average weight was 83.58 ± 21.93 kg, the mean SAPS II score was 44.89 ± 15.02, and the average SOFA score was7.71 ± 3.82. There exists imbalance among the 3 groups in terms of age, weight, SAPS II score, Charlson comorbidity index, and other baseline characteristics. Detailed baseline characteristics data of original cohort are shown in Additional file 4: Table S1.

To mitigate bias arising from the imbalances in baseline characteristics, we employed pairwise PSM: the high and low TWA-BG groups were each matched with the optimal TWA-BG group, respectively, resulting in two matched cohorts (cohort 1: optimal TWA-BG group versus low TWA-BG group; cohort 2: optimal TWA-BG group versus high TWA-BG group). The detailed comparison of before and after PSM of these two cohorts is shown in Tables 1 and 2. After pairwise PSM, the SMDs of all covariates with greater than 0.1 between groups were notably reduced, indicating improved baseline balance between the matched groups (Additional file 4: Table S2-S7, Additional file 3: Fig. S2).

**Table 1:** Baseline characteristics before and after propensity score matching of cohort 1

	Before matching				After matching			
	Overall (*N* = 1029)	Optimal TWA-BG (*N* = 893)	Low TWA-BG (*N* = 136)	SMD	Overall (*N* = 264)	Optimal TWA-BG (*N* = 132)	Low TWA-BG (*N* = 132)	SMD
Age	66.95 (15.54)	67.33 (15.16)	64.51 (17.69)	0.171	63.81 (16.72)	62.67 (15.71)	64.95 (17.65)	0.137
Gender (female)	372 (36.15%)	316 (35.39%)	56 (41.18%)	0.119	108 (40.91%)	54 (40.91%)	54 (40.91%)	< 0.001
Weight	81.94 (22.52)	82.07 (22.02)	81.09 (25.65)	0.041	82.61 (25.70)	83.45 (25.83)	81.77 (25.64)	0.065
SAPS II	43.19 (14.46)	43.26 (14.06)	42.71 (16.92)	0.035	42.79 (15.82)	42.67 (14.51)	42.90 (17.09)	0.014
SOFA score	7.60 (3.83)	7.63 (3.76)	7.40 (4.30)	0.058	7.46 (3.98)	7.53 (3.60)	7.39 (4.33)	0.036
Charlson comorbidity index	5.67 (2.59)	5.64 (2.51)	5.93 (3.08)	0.104	6.00 (3.02)	6.06 (2.93)	5.94 (3.11)	0.04
*Interventions (Boolean for 1st 24 h)*								
CABG (YES)	**78 (7.58%)**	**74 (8.29%)**	**4 (2.94%)**	**0.234**	8 (3.03%)	4 (3.03%)	4 (3.03%)	< 0.001
PCI (YES)	40 (3.89%)	34 (3.81%)	6 (4.41%)	0.03	11 (4.17%)	5 (3.79%)	6 (4.55%)	0.038
IABP (YES)	**164 (15.94%)**	**151 (16.91%)**	**13 (9.56%)**	**0.218**	23 (8.71%)	10 (7.58%)	13 (9.85%)	0.081
PiCCO (YES)	3 (0.29%)	2 (0.22%)	1 (0.74%)	0.074	2 (0.76%)	1 (0.76%)	1 (0.76%)	< 0.001
NICOM (YES)	21 (2.04%)	16 (1.79%)	5 (3.68%)	0.116	5 (1.89%)	2 (1.52%)	3 (2.27%)	0.056
Mechanical ventilation (YES)	**602 (58.50%)**	**545 (61.03%)**	**57 (41.91%)**	**0.39**	118 (44.70%)	62 (46.97%)	56 (42.42%)	0.092
Sedative therapy (YES)	**625 (60.74%)**	**566 (63.38%)**	**59 (43.38%)**	**0.409**	123 (46.59%)	65 (49.24%)	58 (43.94%)	0.106
*Comorbidities (Boolean)*								
HF (YES)	795 (77.26%)	686 (76.82%)	109 (80.15%)	0.081	220 (83.33%)	114 (86.36%)	106 (80.30%)	0.163
Hypertension (YES)	702 (68.22%)	618 (69.20%)	84 (61.76%)	0.157	163 (61.74%)	82 (62.12%)	81 (61.36%)	0.016
AFIB (YES)	247 (24.00%)	216 (24.19%)	31 (22.79%)	0.033	63 (23.86%)	33 (25.00%)	30 (22.73%)	0.053
T2DM (YES)	176 (17.10%)	157 (17.58%)	19 (13.97%)	0.099	47 (17.80%)	28 (21.21%)	19 (14.39%)	0.179
Renal (YES)	335 (32.56%)	286 (32.03%)	49 (36.03%)	0.085	105 (39.77%)	57 (43.18%)	48 (36.36%)	0.14
Liver (YES)	49 (4.76%)	39 (4.37%)	10 (7.35%)	0.127	21 (7.95%)	11 (8.33%)	10 (7.58%)	0.028
COPD (YES)	174 (16.91%)	148 (16.57%)	26 (19.12%)	0.066	47 (17.80%)	22 (16.67%)	25 (18.94%)	0.059
CAD (YES)	**581 (56.46%)**	**519 (58.12%)**	**62 (45.59%)**	**0.253**	126 (47.73%)	65 (49.24%)	61 (46.21%)	0.061
Stroke (YES)	106 (10.30%)	89 (9.97%)	17 (12.50%)	0.08	29 (10.98%)	15 (11.36%)	14 (10.61%)	0.024
Malignancy (YES)	**110 (10.69%)**	**87 (9.74%)**	**23 (16.91%)**	**0.212**	48 (18.18%)	26 (19.70%)	22 (16.67%)	0.079
Cardiomyopathy (YES)	337 (32.75%)	286 (32.03%)	51 (37.50%)	0.115	100 (37.88%)	50 (37.88%)	50 (37.88%)	< 0.001
HVD (YES)	483 (46.94%)	430 (48.15%)	53 (38.97%)	0.186	99 (37.50%)	48 (36.36%)	51 (38.64%)	0.047
*Vital signs (1st 24 h)*								
MAP	78.89 (17.85)	79.14 (17.98)	77.27 (16.98)	0.107	77.05 (17.31)	76.62 (17.58)	77.48 (17.09)	0.05
Temperature	36.43 (0.87)	36.43 (0.87)	36.44 (0.92)	0.01	36.42 (0.90)	36.40 (0.90)	36.44 (0.90)	0.048
Heart rate	90.20 (20.36)	90.66 (20.13)	87.17 (21.66)	0.167	86.83 (20.38)	86.45 (18.73)	87.22 (21.97)	0.038
CVP	15.27 (21.57)	15.30 (22.51)	14.95 (8.96)	0.021	15.12 (7.83)	15.10 (6.82)	15.14 (9.05)	0.006
CO	4.39 (1.59)	4.39 (1.59)	4.47 (1.62)	0.054	4.56 (1.48)	4.67 (1.36)	4.43 (1.66)	0.163
*Laboratory tests (1st 24 h)*								
WBC count	**13.60 (7.32)**	**13.75 (7.07)**	**12.56 (8.74)**	**0.15**	12.40 (7.83)	12.12 (6.72)	12.67 (8.82)	0.07
Hemoglobin	10.61 (2.52)	10.64 (2.54)	10.44 (2.45)	0.081	10.37 (2.37)	10.27 (2.28)	10.47 (2.47)	0.083
Platelet	196.86 (103.14)	194.65 (101.79)	211.40 (110.94)	0.158	207.97 (109.91)	205.14 (109.60)	210.80 (110.57)	0.052
pH	7.35 (0.10)	7.35 (0.10)	7.35 (0.10)	0.031	7.36 (0.10)	7.36 (0.11)	7.35 (0.10)	0.066
PO2	**185.52 (127.29)**	**189.81 (128.89)**	**157.36 (112.66)**	**0.269**	163.19 (116.30)	168.22 (119.86)	158.15 (112.85)	0.087
PCO2	40.61 (11.82)	40.49 (11.67)	41.39 (12.83)	0.074	40.89 (12.35)	40.71 (12.23)	41.06 (12.51)	0.028
Lactate	**2.98 (2.42)**	**3.03 (2.42)**	**2.67 (2.41)**	**0.149**	2.83 (2.51)	2.98 (2.57)	2.67 (2.44)	0.126
Creatinine	**1.86 (1.65)**	**1.83 (1.69)**	**2.04 (1.38)**	**0.137**	2.21 (1.85)	2.38 (2.20)	2.03 (1.39)	0.192
*Outcomes (Boolean)*								
28-day mortality (Death)	**251 (24.39%)**	**204 (22.84%)**	**47 (34.56%)**	**0.261**	87 (32.95%)	42 (31.82%)	45 (34.09%)	0.048
ICU mortality (Death)	156 (15.16%)	129 (14.45%)	27 (19.85%)	0.144	48 (18.18%)	22 (16.67%)	26 (19.70%)	0.079
In-hospital mortality (Death)	**226 (21.96%)**	**186 (20.83%)**	**40 (29.41%)**	**0.199**	75 (28.41%)	38 (28.79%)	37 (28.03%)	0.017
*Length of stay (days)*								
ICU LOS	**7.45 (7.89)**	**7.88 (8.25)**	**4.60 (3.86)**	**0.51**	**5.73 (4.87)**	**6.95 (5.46)**	**4.51 (3.83)**	**0.519**
In-hospital LOS	**14.83 (16.99)**	**15.31 (17.46)**	**11.66 (13.13)**	**0.237**	**13.34 (15.74)**	**15.35 (17.82)**	**11.34 (13.09)**	**0.257**

Values are presented as mean (standard deviation) for continuous variables and number (percentage) for categorical variables

Variables in bold indicate statistical significance (p-value < 0.05)

**Table 2:** Baseline characteristics before and after propensity score matching of cohort 2

	Before matching				After matching			
	Overall (*N* = 1877)	Optimal TWA-BG (*N* = 893)	High TWA-BG (*N* = 984)	SMD	Overall (*N* = 1058)	Optimal TWA-BG (*N* = 529)	High TWA-BG (*N* = 529)	SMD
Age	**68.51 (14.01)**	**67.33 (15.16)**	**69.58 (12.78)**	**0.16**	68.07 (14.93)	68.44 (15.31)	67.70 (14.54)	0.049
Gender (Female)	677 (36.07%)	316 (35.39%)	361 (36.69%)	0.027	385 (36.39%)	191 (36.11%)	194 (36.67%)	0.012
Weight	**83.75 (21.63)**	**82.07 (22.02)**	**85.26 (21.17)**	**0.148**	83.22 (21.82)	83.38 (22.29)	83.05 (21.37)	0.015
SAPS II	**45.05 (14.86)**	**43.26 (14.06)**	**46.67 (15.39)**	**0.231**	44.79 (14.93)	44.94 (14.62)	44.65 (15.25)	0.019
SOFA score	7.74 (3.79)	7.63 (3.76)	7.83 (3.81)	0.053	7.57 (3.79)	7.58 (3.80)	7.56 (3.78)	0.005
Charlson comorbidity index	**6.33 (2.65)**	**5.64 (2.51)**	**6.97 (2.62)**	**0.518**	6.18 (2.56)	6.27 (2.52)	6.10 (2.60)	0.066
*Interventions (Boolean for 1st 24 h)*								
CABG (YES)	**122 (6.50%)**	**74 (8.29%)**	**48 (4.88%)**	**0.138**	55 (5.20%)	25 (4.73%)	30 (5.67%)	0.043
PCI (YES)	75 (4.00%)	34 (3.81%)	41 (4.17%)	0.018	50 (4.73%)	26 (4.91%)	24 (4.54%)	0.018
IABP (YES)	313 (16.68%)	151 (16.91%)	162 (16.46%)	0.012	188 (17.77%)	89 (16.82%)	99 (18.71%)	0.049
PiCCO (YES)	7 (0.37%)	2 (0.22%)	5 (0.51%)	0.047	4 (0.38%)	2 (0.38%)	2 (0.38%)	< 0.001
NICOM (YES)	**53 (2.82%)**	**16 (1.79%)**	**37 (3.76%)**	**0.12**	27 (2.55%)	15 (2.84%)	12 (2.27%)	0.036
Mechanical ventilation (YES)	1111 (59.19%)	545 (61.03%)	566 (57.52%)	0.071	614 (58.03%)	304 (57.47%)	310 (58.60%)	0.023
Sedative therapy (YES)	**1142 (60.84%)**	**566 (63.38%)**	**576 (58.54%)**	**0.099**	637 (60.21%)	315 (59.55%)	322 (60.87%)	0.027
*Comorbidities (Boolean)*								
HF (YES)	**1490 (79.38%)**	**686 (76.82%)**	**804 (81.71%)**	**0.121**	851 (80.43%)	427 (80.72%)	424 (80.15%)	0.014
Hypertension (YES)	**1387 (73.89%)**	**618 (69.20%)**	**769 (78.15%)**	**0.204**	764 (72.21%)	383 (72.40%)	381 (72.02%)	0.008
AFIB (YES)	438 (23.34%)	216 (24.19%)	222 (22.56%)	0.038	255 (24.10%)	129 (24.39%)	126 (23.82%)	0.013
T2DM (YES)	**755 (40.22%)**	**157 (17.58%)**	**598 (60.77%)**	**0.987**	321 (30.34%)	156 (29.49%)	165 (31.19%)	0.037
Renal (YES)	**732 (39.00%)**	**286 (32.03%)**	**446 (45.33%)**	**0.276**	393 (37.15%)	202 (38.19%)	191 (36.11%)	0.043
Liver (YES)	68 (3.62%)	39 (4.37%)	29 (2.95%)	0.076	35 (3.31%)	16 (3.02%)	19 (3.59%)	0.032
COPD (YES)	310 (16.52%)	148 (16.57%)	162 (16.46%)	0.003	173 (16.35%)	84 (15.88%)	89 (16.82%)	0.026
CAD (YES)	**1169 (62.28%)**	**519 (58.12%)**	**650 (66.06%)**	**0.164**	646 (61.06%)	326 (61.63%)	320 (60.49%)	0.023
Stroke (YES)	166 (8.84%)	89 (9.97%)	77 (7.83%)	0.075	86 (8.13%)	45 (8.51%)	41 (7.75%)	0.028
Malignancy (YES)	203 (10.82%)	87 (9.74%)	116 (11.79%)	0.066	115 (10.87%)	61 (11.53%)	54 (10.21%)	0.043
Cardiomyopathy (YES)	569 (30.31%)	286 (32.03%)	283 (28.76%)	0.071	332 (31.38%)	166 (31.38%)	166 (31.38%)	< 0.001
HVD (YES)	**837 (44.59%)**	**430 (48.15%)**	**407 (41.36%)**	**0.137**	452 (42.72%)	228 (43.10%)	224 (42.34%)	0.015
*Vital signs (1st 24 h)*								
MAP	**78.49 (18.82)**	**79.14 (17.98)**	**77.90 (19.54)**	**0.066**	79.05 (18.64)	79.04 (16.86)	79.06 (20.27)	0.001
Temperature	**36.47 (0.88)**	**36.43 (0.87)**	**36.51 (0.89)**	**0.098**	36.52 (0.85)	36.51 (0.80)	36.53 (0.90)	0.02
Heart rate	91.41 (20.53)	90.66 (20.13)	92.10 (20.88)	0.07	92.43 (20.70)	92.29 (20.55)	92.56 (20.87)	0.013
CVP	15.22 (19.51)	15.30 (22.51)	15.14 (15.64)	0.008	14.12 (7.03)	14.18 (7.27)	14.06 (6.78)	0.017
CO	4.46 (1.52)	4.39 (1.59)	4.59 (1.39)	0.135	4.50 (1.46)	4.45 (1.52)	4.59 (1.37)	0.092
*Laboratory tests (1st 24 h)*								
WBC count	**14.29 (9.31)**	**13.75 (7.07)**	**14.78 (10.93)**	**0.112**	14.21 (7.72)	14.22 (7.71)	14.21 (7.74)	0.002
Hemoglobin	10.64 (2.47)	10.64 (2.54)	10.64 (2.42)	< 0.001	10.81 (2.52)	10.82 (2.57)	10.81 (2.48)	0.002
Platelet	**202.61 (101.30)**	**194.65 (101.79)**	**209.84 (100.35)**	**0.15**	205.15 (101.30)	206.31 (107.18)	203.99 (95.15)	0.023
pH	**7.34 (0.11)**	**7.35 (0.10)**	**7.34 (0.11)**	**0.117**	7.34 (0.11)	7.34 (0.11)	7.35 (0.11)	0.023
PO2	**175.00 (123.67)**	**189.81 (128.89)**	**161.55 (117.19)**	**0.23**	172.95 (122.15)	174.19 (123.94)	171.71 (120.43)	0.02
PCO2	40.59 (12.17)	40.49 (11.67)	40.69 (12.61)	0.017	40.34 (11.86)	40.22 (11.44)	40.47 (12.27)	0.022
Lactate	3.15 (2.55)	3.03 (2.42)	3.25 (2.66)	0.087	3.15 (2.58)	3.14 (2.56)	3.16 (2.60)	0.007
Creatinine	**1.93 (1.56)**	**1.83 (1.69)**	**2.02 (1.43)**	**0.119**	1.85 (1.45)	1.86 (1.54)	1.85 (1.35)	0.004
*Outcomes (Boolean)*								
28-day mortality (Death)	**551 (29.36%)**	**204 (22.84%)**	**347 (35.26%)**	**0.276**	**317 (29.96%)**	**132 (24.95%)**	**185 (34.97%)**	**0.22**
ICU mortality (Death)	**374 (19.93%)**	**129 (14.45%)**	**245 (24.90%)**	**0.265**	**215 (20.32%)**	**81 (15.31%)**	**134 (25.33%)**	**0.251**
In-hospital mortality (Death)	**503 (26.80%)**	**186 (20.83%)**	**317 (32.22%)**	**0.26**	**291 (27.50%)**	**120 (22.68%)**	**171 (32.33%)**	**0.217**
*Length of stay (days)*								
ICU LOS	7.75 (8.11)	7.88 (8.25)	7.62 (7.99)	0.031	7.77 (8.23)	7.88 (8.03)	7.66 (8.44)	0.026
In-hospital LOS	**14.66 (15.84)**	**15.31 (17.46)**	**14.08 (14.19)**	**0.077**	**15.29 (17.78)**	**16.45 (20.65)**	**14.12 (14.26)**	**0.131**

Values are presented as mean (standard deviation) for continuous variables and number (percentage) for categorical variables

Variables in bold indicate statistical significance (p-value < 0.05)

### Primary and secondary outcome

**Fig. 3:**
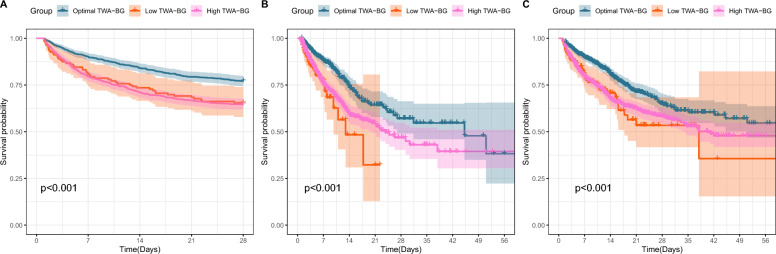
Unadjusted Kaplan–Meier survival curve for primary and secondary outcome of original cohort. **A** Unadjusted Kaplan–Meier survival curve for 28-day mortality. **B** Unadjusted Kaplan–Meier survival curve for ICU mortality. **C** Unadjusted Kaplan–Meier survival curve for in-hospital mortality

In our study population of 2013 patients with cardiogenic shock, the incidence of 28-day mortality was 22.84% (204 patients) in the optimal TWA-BG group, 34.56% (47 patients) and 35.26% (347 patients) in the low and high TWA-BG groups. The unadjusted Kaplan–Meier survival analysis revealed the lowest 28-day mortality in the optimal TWA-BG group compared to the low and high TWA-BG groups (Fig. 3A). The unadjusted log-rank test further revealed that the low TWA-BG group (HR:1.67, 95% CI: 1.19–2.33, *p* = 0.002) and high TWA-BG group (HR:1.72, 95% CI: 1.46–2.03, *p* < 0.001) both carried more than 1.6 times higher risk of 28-day mortality than the optimal TWA-BG group (Additional file 4: Table S8).

Similar trends were observed for both ICU mortality and in-hospital mortality. The unadjusted Kaplan–Meier curves delineated the difference in ICU mortality (Fig. 3B) and in-hospital mortality (Fig. 3C) among groups. The unadjusted log-rank test revealed increased risks of ICU mortality in the low TWA-BG group (HR: 2.30, 95% CI: 1.40–3.79, *p* < 0.001) and the high TWA-BG group (HR: 1.77, 95% CI: 1.45–2.17, *p* < 0.001) compared to the optimal TWA-BG group (Additional file 4: Table S9). In terms of in-hospital mortality, the unadjusted log-rank test indicated unfavorable prognosis in the low TWA-BG group (HR: 1.73, 95% CI: 1.19–2.51, *p* = 0.001) and the high TWA-BG group (HR: 1.64, 95% CI: 1.38–1.95, *p* < 0.001) relative to the optimal TWA-BG group (Additional file 4: Table S10).

### Sensitivity analysis and subgroup analysis

To validate the robustness of our findings, we employed a series of models to perform the sensitivity analysis. These models comprise the following: multivariate Cox model adjusted with all covariates, multivariate Cox model adjusted with unbalanced covariates, Cox model adjusted with all covariates using IPTW, and survey-weighted Cox model adjusted with all covariates using IPTW. As summarized in Table 3, all estimated models converge towards the same conclusion: patients with TWA-BG levels ranging from 104–138 mg/dl exhibit the lowest mortality risk (including 28-day, ICU, and in-hospital mortality). Detailed estimates of covariates for each model can be found in Additional file 4: Table S11–S34.

**Table 3:** Primary and secondary outcome analyses with different models for cohort 1 and cohort 2

	Cohort 1 ^1^	*p*-value	Cohort 2 ^2^	*p*-value
*28-day mortality*				
Log-rank test [HR (95% CI)]^3^	**1.67 (1.19, 2.33)**	** < 0.01**	**1.72 (1.46, 2.03)**	** < 0.001**
Multivariate Cox model adjusted with all covariates [HR (95% CI)]^3^	**1.58 (1.12, 2.22)**	** < 0.01**	**1.15 (1.08, 1.22)**	** < 0.001**
Multivariate Cox model adjusted with unbalanced covariates [HR (95% CI)]^3^	**1.61 (1.16, 2.25)**	** < 0.01**	**1.15 (1.08, 1.22)**	** < 0.001**
Cox model adjusted with all covariates using IPTW [HR (95% CI)]^3^	**1.89 (1.16, 3.07)**	** < 0.05**	**1.38 (1.08, 1.76)**	** < 0.01**
Survey-weighted Cox model adjusted with all covariates using IPTW [HR (95% CI)]^3^	**1.91 (1.17, 3.12)**	** < 0.01**	**1.10 (1.02, 1.18)**	** < 0.05**
*ICU mortality*				
Log-rank test [HR (95% CI)]^3^	**2.30 (1.40, 3.79)**	** < 0.001**	**1.77 (1.45, 2.17)**	** < 0.001**
Multivariate Cox model adjusted with all covariates [HR (95% CI)]^3^	**2.29 (1.41, 3.73)**	** < 0.001**	**1.87 (1.45, 2.41)**	** < 0.001**
Multivariate Cox model adjusted with unbalanced covariates [HR (95% CI)]^3^	**2.30 (1.46, 3.62)**	** < 0.001**	**1.82 (1.42, 2.34)**	** < 0.001**
Cox model adjusted with all covariates using IPTW [HR (95% CI)]^3^	**1.38 (1.12, 1.71)**	** < 0.01**	**1.38 (1.01, 1.87)**	** < 0.05**
Survey-weighted Cox model adjusted with all covariates using IPTW [HR (95% CI)]^3^	**2.81 (1.42, 5.57)**	** < 0.01**	**1.38 (1.01, 1.88)**	** < 0.05**
*In-hospital mortality*				
Log-rank test [HR (95% CI)]^3^	**1.73 (1.19, 2.51)**	** < 0.01**	**1.64 (1.38, 1.95)**	** < 0.001**
Multivariate Cox model adjusted with all covariates [HR (95% CI)]^3^	**1.89 (1.29, 2.75)**	** < 0.001**	**1.66 (1.34, 2.06)**	** < 0.001**
Multivariate Cox model adjusted with unbalanced covariates [HR (95% CI)]^3^	**1.95 (1.35, 2.80)**	** < 0.001**	**1.15 (1.08, 1.23)**	** < 0.001**
Cox model adjusted with all covariates using IPTW [HR (95% CI)]^3^	**2.07 (1.23, 3.48)**	** < 0.01**	**1.32 (1.02, 1.71)**	** < 0.05**
Survey-weighted Cox model adjusted with all covariates using IPTW [HR (95% CI)]^3^	**2.09 (1.24, 3.52)**	** < 0.01**	**1.08 (1.00, 1.17)**	** < 0.05**

Statistical analyses of different models with *p*-value < 0.05 were displayed in bold

^1^Cohort 1: optimal TWA-BG (104 < TWA-BG ≤ 138 mg/dL) versus low TWA-BG (TWA-BG ≤ 104 mg/dL)

^2^Cohort 2: optimal TWA-BG (104 < TWA-BG ≤ 138 mg/dL) versus high TWA-BG (TWA-BG > 138 mg/dL)

^3^HR = hazard ratio, CI = confidence interval

**Fig. 4:**
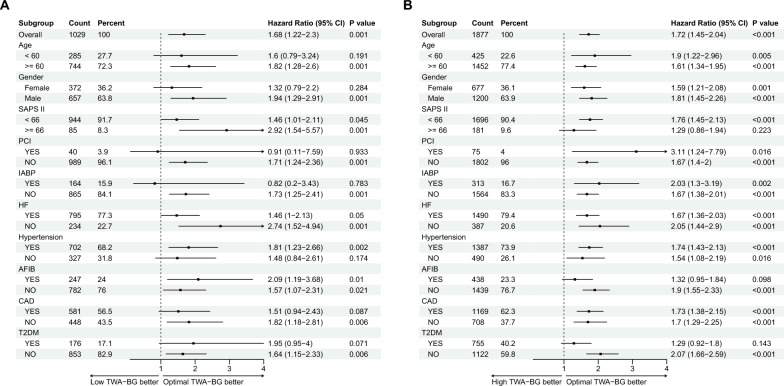
Forest plot of subgroup analysis for 28-day mortality in cohort 1 (**A**) and cohort 2 (**B**)

Furthermore, we conducted a stratified analysis of the two cohorts based on age, gender, SAPS II score [23], PCI, IABP, HF, Hypertension, AFIB, CAD, and T2DM. As depicted in Fig. 4, for cohort 1, patients older than 60 years, male, SAPS II score ≥ 66, without PCI, IABP, HF, AFIB, CAD, T2DM, and with hypertension, displayed a higher 28-day mortality risk in the low TWA-BG group compared to the optimal TWA-BG group. Conversely, in cohort 2, the high TWA-BG group exhibited a higher 28-day mortality risk across most strata. Similar outcomes were observed for ICU mortality rate and in-hospital mortality rate (Additional file 3: Figs. S3, S4).

## Discussion

In our study, by analyzing time series BG data from a large sample of patients with cardiogenic shock, we have identified a potential optimal level for BG control. The research reveals that patients with 104 < TWA-BG ≤ 138 mg/dl exhibit the most favorable outcomes in terms of 28-day, in-hospital, and ICU mortality. Through control bias with PSM-based IPTW and subsequent sensitivity analysis with multiple models, this conclusion remains robust. It suggested that maintaining BG levels within this optimal range yields the best clinical prognosis, as deviations towards higher or lower BG levels result in increased mortality. Our study expands upon previous research and provides clinicians with reasonable evidence for BG control in patients with cardiogenic shock.

Previous research has extensively discussed the correlation between hyperglycemia and adverse prognosis in cardiovascular diseases. Critically ill patients often experience disturbances in glucose metabolism or stress-induced hyperglycemia [24], leading to elevated BG levels. Excessively high BG levels can disrupt cellular function in patients, including elevated cytokines, increased oxidative stress, and impaired nitric oxide production [25–27]. These factors can activate inflammatory responses, subsequently causing secondary damage to the heart. Elevated fasting BG [7], admission hyperglycemia [4], elevated peak BG levels during hospitalization [10], and random BG elevations [9] are all associated with poor outcomes in cardiac patients. These outcomes include higher in-hospital mortality rates, increased incidence of major adverse cardiovascular events, and a greater likelihood of developing cardiogenic shock. A study from Poland analyzed 258 patients with cardiogenic shock, comparing those with hyperglycemia upon admission to those with BG levels < 7.8 mmol/L. The study revealed that patients with elevated BG had a higher in-hospital mortality rate (41.5% vs. 28%, *p* = 0.041), as well as higher 1-year (51.4% vs. 34.7%, *p* = 0.015) and 5-year mortality rates (65.8% vs. 43.3%, *p* = 0.034) [8]. Another study conducted in China, which included 2412 patients with acute myocardial infarction, also found that compared to individuals with BG levels below 140 mg/dL, patients with BG levels ranging from 140–200 mg/dL (odds ratio (OR): 1.68, 95% CI: 1.21–2.31) and above 200 mg/dL (OR: 3.72, 95% CI: 2.50–5.46) had a higher risk of developing cardiogenic shock [9]. Insulin therapy serves as the primary approach to manage hyperglycemic events in critically ill patients [28].

The use of insulin can protect mitochondrial function, reduce inflammation factors, and mitigate endothelial activation, thereby exerting a cardioprotective effect [29]. Excessively strict BG control often carries the risk of hypoglycemic events [30, 31]. Low BG levels can also lead to adverse outcomes in cardiac patients [32–34]. A study from Korea found that among heart failure patients upon admission, those with BG levels < 3.9 mmol/L and concomitant type 2 diabetes had a higher risk of 3-point major cardiovascular event (3P-MACE) (HR: 2.29, 95% CI: 1.04–5.06) and increased all-cause mortality (HR: 2.58, 95% CI: 1.26–5.31) compared to patients without severe hypoglycemia or diabetes [12]. Moreover, a greater number of occurrences of hypoglycemic events were associated with adverse cardiovascular outcomes and increased all-cause mortality [11], and iatrogenic hypoglycemic events can also contribute to poor prognosis in critically ill patients [35]. However, research has shown that insulin therapy-induced hypoglycemia can be better detected and promptly treated in ICU patients, thereby reducing adverse events caused by low BG [36]. For patients without early parenteral nutrition in ICU, tight BG control (maintaining BG within the range of 80–110 mg/dl) does not increase ICU length of stay or 90-day mortality. Moreover, there is no significant difference in the incidence of severe hypoglycemia [37]. Current guidelines also recommend maintaining BG levels between 6.1–7.8 mmol/L (109.8–140.4 mg/dl) for ICU patients without diabetes. For critically ill patients newly admitted, the interval for BG monitoring should not exceed 1 h until stable BG and insulin infusion levels are achieved [28]. This provides a foundation for more precise BG control. Additionally, with the introduction of various BG control and insulin treatment protocols, protocol-driven BG monitoring can standardize monitoring practices, potentially reducing the frequency of BG measurements [38].

The CardShock study revealed a significant correlation between admission BG levels and in-hospital mortality in patients with cardiogenic shock. Patients with severe hyperglycemia (≥ 16.0 mmol/L) and hypoglycemia (< 4.0 mmol/L) have the highest rates of in-hospital mortality. Severe hyperglycemia emerges as an independent predictive factor for in-hospital mortality in patients with cardiogenic shock (OR: 3.7, 95% CI: 1.19–11.7, *p* = 0.02) [5]. Hence, there has been a continuous endeavor to identify a rational BG control level. As early as 2013, researchers discovered that strict BG control yields better outcomes compared to lenient control for critically ill patients [39]. Another trial compared the prognosis of cardiac and thoracic surgical patients with BG control at 80–110 mg/dl versus 90–140 mg/dl. The results revealed lower 30-day mortality in the 80–110 mg/dl group, indicating that stringent BG control contributes to improved prognosis in cardiac patients [40]. However, to date, despite considerable academic research, a robust consensus on optimal BG level for patients during hospitalization specifically for patients with cardiogenic shock remains elusive. Therefore, our study provides novel insight and strategies regarding BG control during hospitalization for critically ill patients with cardiogenic shock.

The aforementioned study shares a common limitation, namely the assessment of BG based solely on single BG measurements or extreme values. However, in clinical practice, patients' BG levels may exhibit dynamic fluctuations during the progression of the disease or treatment. Therefore, relying solely on single BG values or extremes does not adequately reflect the overall control of patients' BG levels throughout their hospitalization.

In addition, in comparison to previous findings, our discoveries possess distinct advantages. Firstly, we selected TWA-BG as the target for BG control, which better reflects the overall BG control level throughout the hospitalization process compared to fasting BG, admission BG, or the highest and lowest BG values. Secondly, through extensive analysis of large-scale data, we identified a reasonable BG control level. This target range of 104–138 mg/dl is clinically feasible to achieve, particularly in situations where continuous BG monitoring methods are lacking. Selecting a reasonable range not only facilitates better attainment of the target but also reduces the occurrence of hypoglycemic events.

The sensitivity analysis further corroborated the robustness of the results, confirming that TWA-BG within the range of 104–138 mg/dl exhibited a better prognosis compared to excessively high or low BG levels. Subgroup analyses also supported these findings. Adverse outcomes have been observed with both excessively high and low BG levels across varying disease severities, etiologies of cardiogenic shock, comorbidities, age groups, and treatment modalities. This underscores the importance of maintaining BG within an optimal range to improve the 28-day, ICU, and in-hospital mortality rates in patients with cardiogenic shock. Despite the occurrence of nonsignificant outcomes in a minority of strata, the overall trend remained consistent with the previously mentioned results, indicating an increased mortality rate with both excessively high and low BG levels. The nonsignificance in the low TWA-BG group might be attributed to the relatively smaller sample volume, potentially underestimating the actual adverse effects of low BG. Further prospective trials are necessary to validate these particular findings.

Our study has several limitations. Firstly, it is a retrospective study, and therefore, subsequent prospective, randomized controlled trials are needed to confirm our conclusions. Secondly, the number of patients in the hypoglycemia group within our study population is significantly lower compared to the other two groups, and data on severely hypoglycemic patients are even scarcer. Therefore, further validation of the lower cut-off value of the optimal BG level may require a larger sample size. Thirdly, due to variations in the frequency of BG measurements in the database, there may be undetected episodes of BG abnormalities. Although we conducted time series imputation to address this issue, a more rigorous randomized controlled study is still necessary to mitigate this bias. Lastly, despite controlling for all possible confounding factors, there may be unmeasured confounders that could affect the results. Moreover, due to inherent limitations in the database, a substantial number of missing values were identified for crucial parameters related to cardiogenic shock, such as cardiac function, cardiac output, and filling pressures. Including these variables in the analysis could introduce further bias. Hence, this study opted not to incorporate these variables. Future randomized controlled trials should comprehensively include these parameters to assess their potential impact on outcomes more thoroughly.

## Conclusion

In our retrospective observational study, an optimal glycemic control threshold in the ICU for critically ill patients suffering from cardiogenic shock was identified between the level of 104–138 mg/dL through analysis of time series BG records. Within this target range of BG, included patients exhibit the most favorable outcomes, as evidenced by minimized risks of 28-day, ICU, and in-hospital mortality. Further rigorously and strictly designed randomized controlled trials with time series BG records are needed to validate our findings.

### Supplementary Information

**Additional file 1. **Blood glucose frequency distribution.

**Additional file 2. **Blood glucose range frequency distribution.

**Additional file 3: Fig. S1.** TWA-BG trend in ICU for the first 24 h (A), 48 h (B), 72 h (C) and 7 days (D). Fig. S2 Change in SMD before and after matching of cohort 1 (A) and cohort 2 (B). Fig. S3 Forest plot of subgroup analysis for ICU mortality in cohort 1 (A) and cohort 2 (B). Fig. S4 Forest plot of subgroup analysis for in-hospital mortality in cohort 1 (A) and cohort 2 (B).

**Additional file 4: Table S1.** Basic demographic characteristics of the original cohort. **Table S2.** Standardized mean difference (SMD) of covariates before and after propensity score matching of cohort 1. **Table S3.** Baseline characteristics before propensity score matching of cohort 1. **Table S4.** Baseline characteristics after propensity score matching of cohort 1. **Table S5.** Standardized mean difference (SMD) of covariates before and after propensity score matching of cohort 2.** Table S6.** Baseline characteristics before propensity score matching of cohort 2. **Table S7.** Baseline characteristics after propensity score matching of cohort 2. **Table S8.** Unadjusted log-rank test for 28-day mortality of original cohort. **Table S9.** Unadjusted log-rank test for ICU mortality of original cohort. **Table S10.** Unadjusted log-rank test for in-hospital mortality of original cohort. **Table S11.** Multivariate Cox model adjusted with all covariates for 28-day mortality of original cohort 1. **Table S12.** Multivariate Cox model adjusted with unbalanced covariates for 28-day mortality of original cohort 1. **Table S13.** Multivariate Cox model adjusted with all covariates and IPTW for 28-day mortality of cohort 1. **Table S14.** Survey-weighted Cox model adjusted with all covariates and IPTW for 28-day mortality of cohort 1. **Table S15.** Multivariate Cox model adjusted with all covariates for ICU mortality of original cohort 1. **Table S16.** Multivariate Cox model adjusted with unbalanced covariates for ICU mortality of original cohort 1. **Table S17.** Multivariate Cox model adjusted with all covariates and IPTW for ICU mortality of cohort 1. **Table S18.** Survey-weighted Cox model adjusted with all covariates and IPTW for ICU mortality of cohort 1. **Table S19.** Multivariate Cox model adjusted with all covariates for in-hospital mortality of original cohort 1. **Table S20.** Multivariate Cox model adjusted with unbalanced covariates for in-hospital mortality of original cohort 1. **Table S21.** Multivariate Cox model adjusted with all covariates and IPTW for in-hospital mortality of cohort 1. **Table S22.** Survey-weighted Cox model adjusted with all covariates and IPTW for in-hospital mortality of cohort 1. **Table S23.** Multivariate Cox model adjusted with all covariates for 28-day mortality of original cohort 2. **Table S24.** Multivariate Cox model adjusted with unbalanced covariates for 28-day mortality of original cohort 2. **Table S25.** Multivariate Cox model adjusted with all covariates and IPTW for 28-day mortality of cohort. **Table S26.** Survey-weighted Cox model adjusted with all covariates and IPTW for 28-day mortality of cohort 2. **Table S27.** Multivariate Cox model adjusted with all covariates for ICU mortality of original cohort 2. **Table S28.** Multivariate Cox model adjusted with unbalanced covariates for ICU mortality of original cohort 2. **Table S29.** Multivariate Cox model adjusted with all covariates and IPTW for ICU mortality of cohort 2. **Table S30.** Survey-weighted Cox model adjusted with all covariates and IPTW for ICU mortality of cohort 2. **Table S31.** Multivariate Cox model adjusted with all covariates for in-hospital mortality of original cohort 2. **Table S32.** Multivariate Cox model adjusted with unbalanced covariates for in-hospital mortality of original cohort 2. **Table S33.** Multivariate Cox model adjusted withall covariates and IPTW for in-hospital mortality of cohort 2. **Table S34.** Survey-weighted Cox model adjusted with all covariates and IPTW for in-hospital mortality of cohort 2.

## Data Availability

The MIMIC-IV database is publicly available on PhysioNet (https://www.physionet.org/). Concepts codes are available in the MIMIC Code Repository (​​https://github.com/MIT-LCP/mimic-code/).

## Abbreviations

BG: Blood glucose
ICU: Intensive care unit
MIMIC: Medical Information Mart for Intensive Care
TWA-BG: Time-weighted average blood glucose
RECORD: REporting of studies Conducted using Observational Routinely-collected health Data
ICD: International Classification of Disease
SOFA: Sequential Organ Failure Assessment
SAPS II: Simplified Acute Physiology Score II
SQL: Structured query language
RCS: Restricted cubic spline
CABG: Coronary artery bypass grafting
PCI: Percutaneous coronary intervention
IABP: Intra-aortic balloon pump
PiCCO: Pulse index continuous cardiac output
NICOM: Non-invasive cardiac output monitoring
HF: Heart failure
AFIB: Atrial fibrillation
T2DM: Type 2 diabetes mellitus
COPD: Chronic obstructive pulmonary disease
CAD: Coronary artery disease
HVD: Heart valvular disease
MAP: Mean arterial pressure
WBC: White blood cell
pH: Potential of hydrogen
PO2: Partial pressure of oxygen
PCO2: Partial pressure of carbon dioxide
CO: Cardiac output
CVP: Central venous pressure
PSM: Propensity score matching
IPTW: Inverse probability of treatment weighting
SMD: Standardized mean difference
CI: Confidence interval
HR: Hazard ratio
OR: Odds ratio
3P-MACE: 3-Point major cardiovascular events
