# Supplementary figures and images for: The optimal blood glucose is significantly associated with lower mortality in critically ill patients with cardiogenic shock: an analysis revealed with time series blood glucose records
Source: Eur J Med Res. 2024 Feb 17;29:129. doi: 10.1186/s40001-024-01724-8 (PMC10874009; doi:10.1186/s40001-024-01724-8)

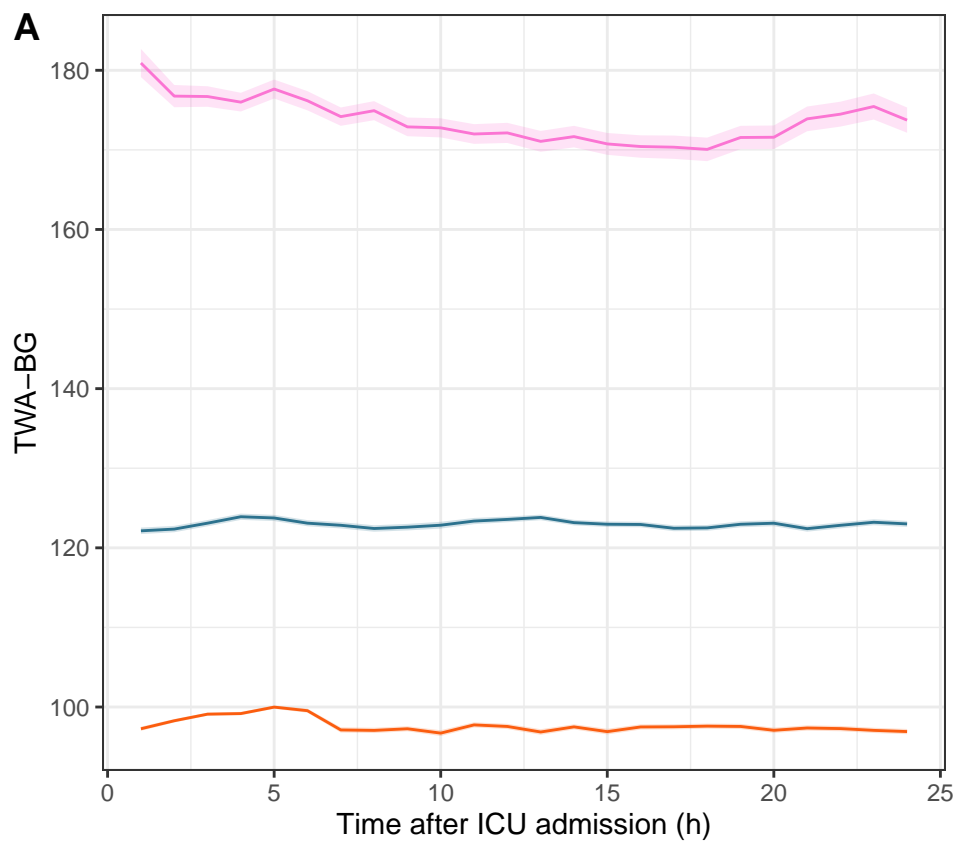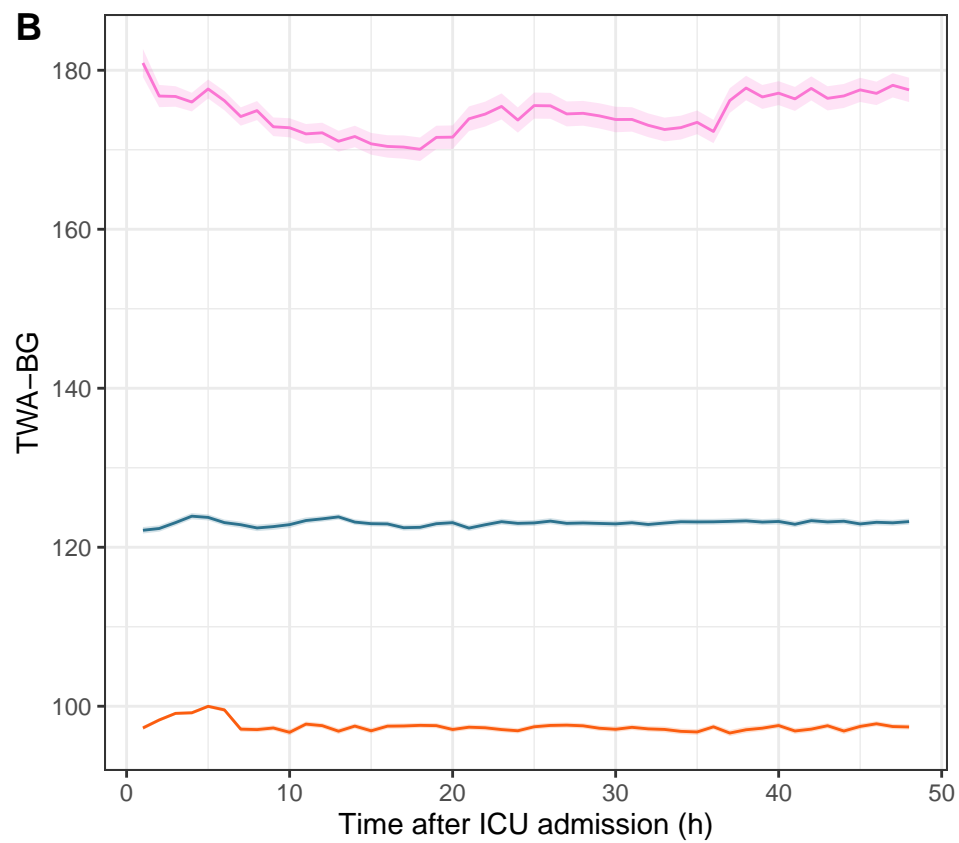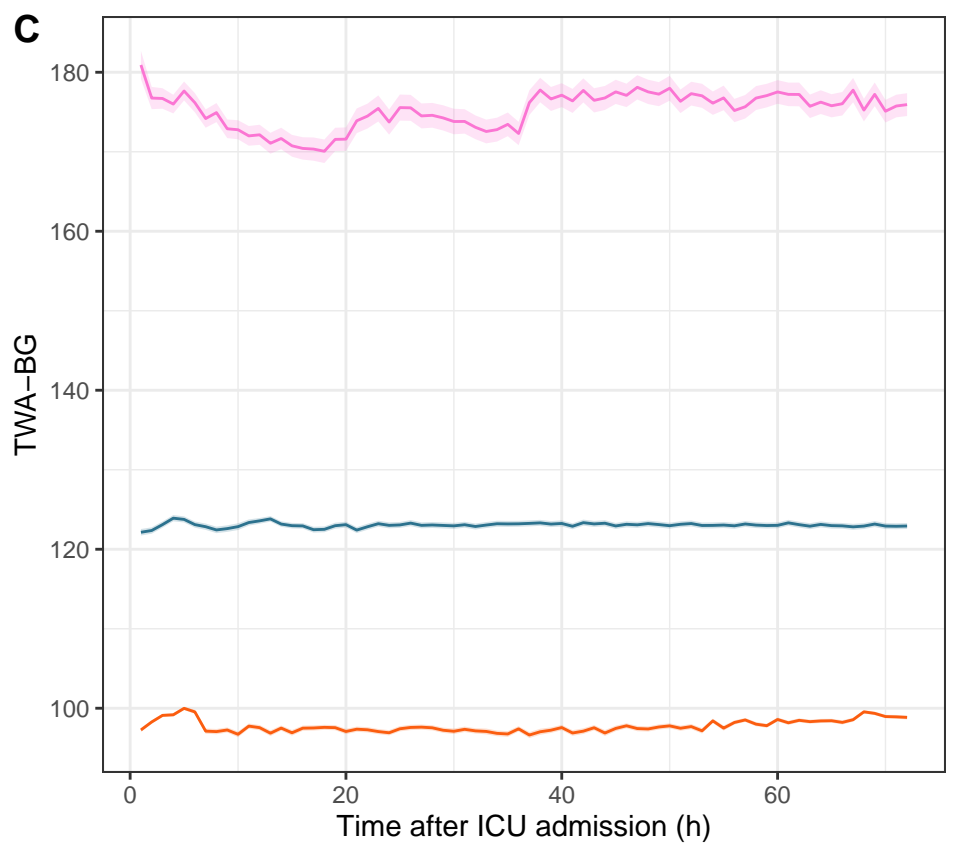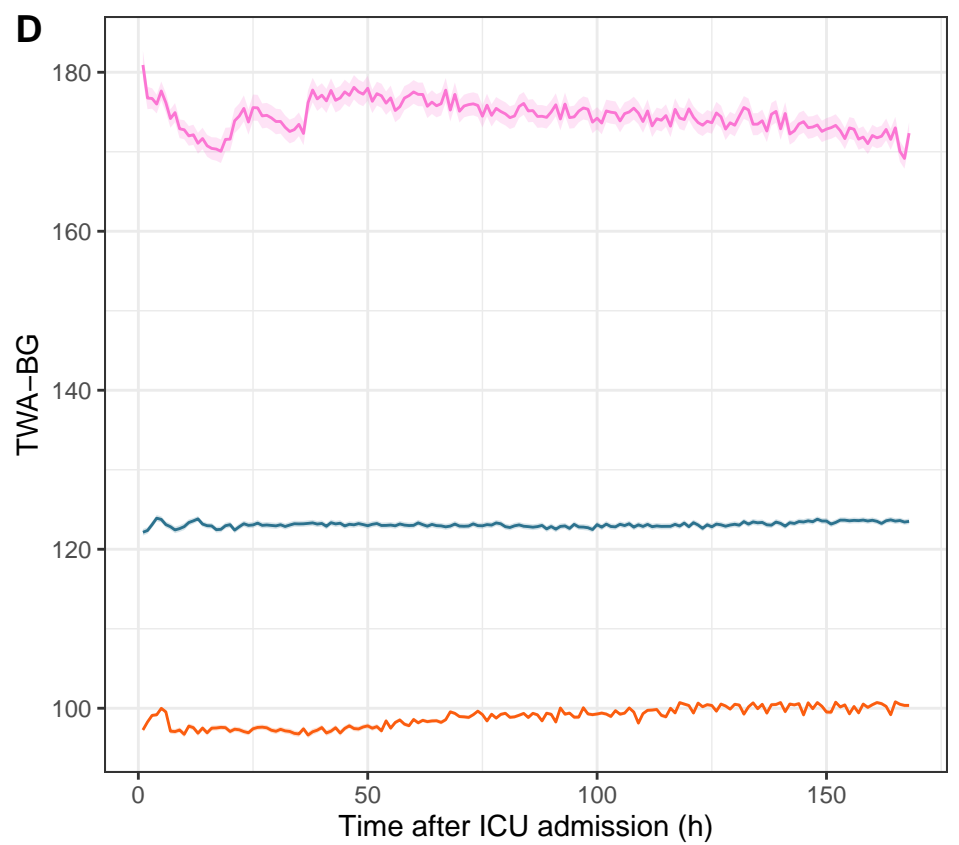

**A**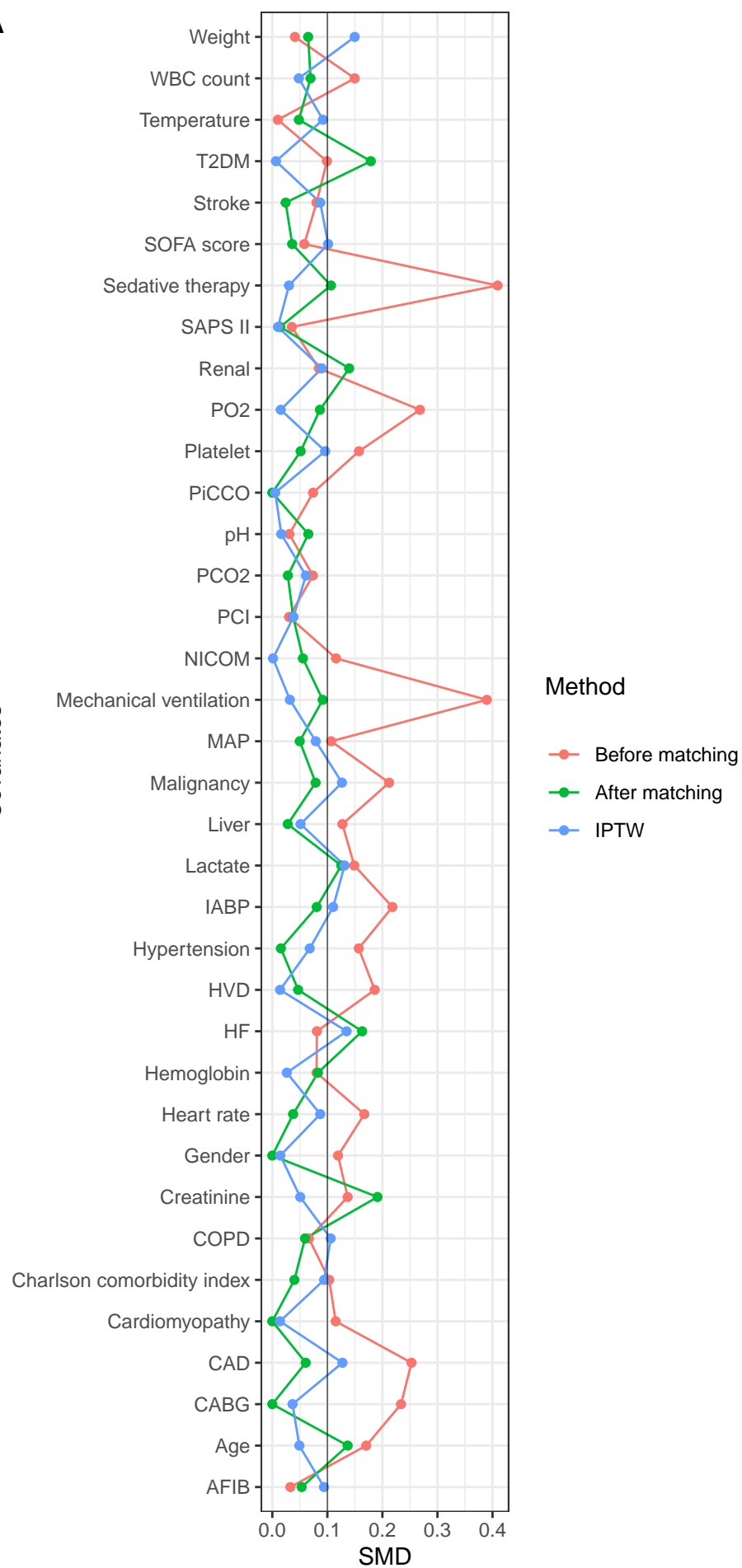**B**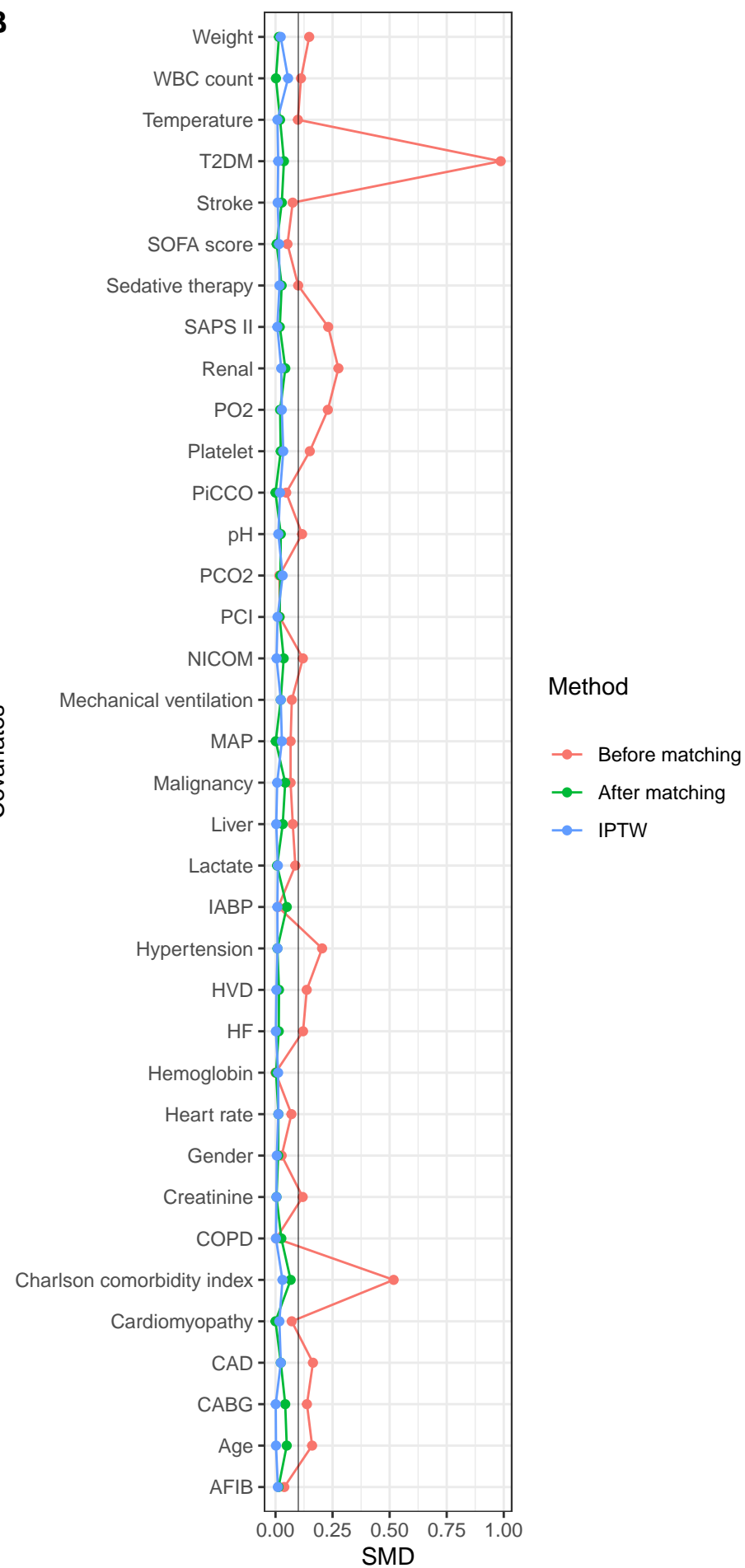

**A**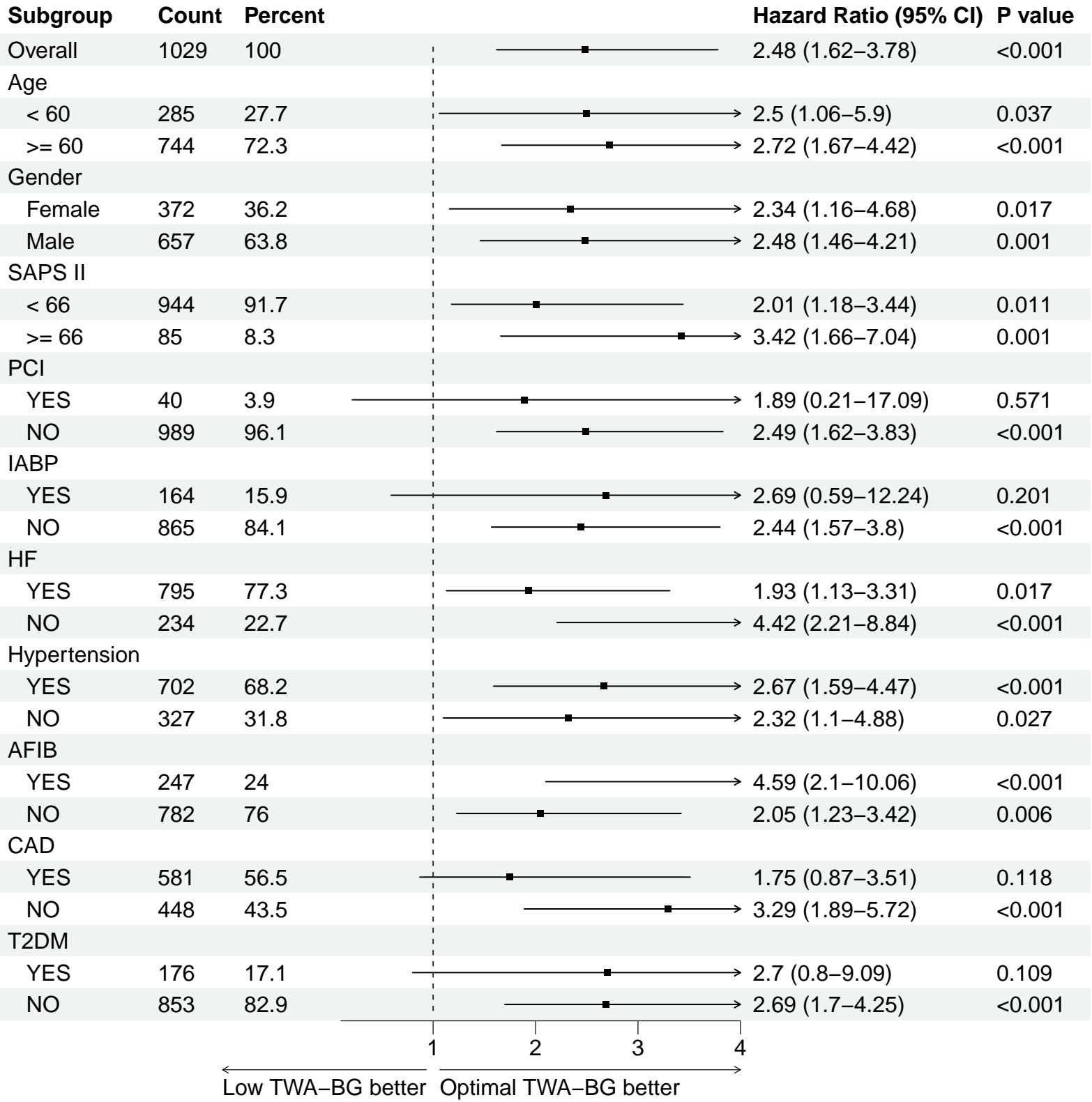**B**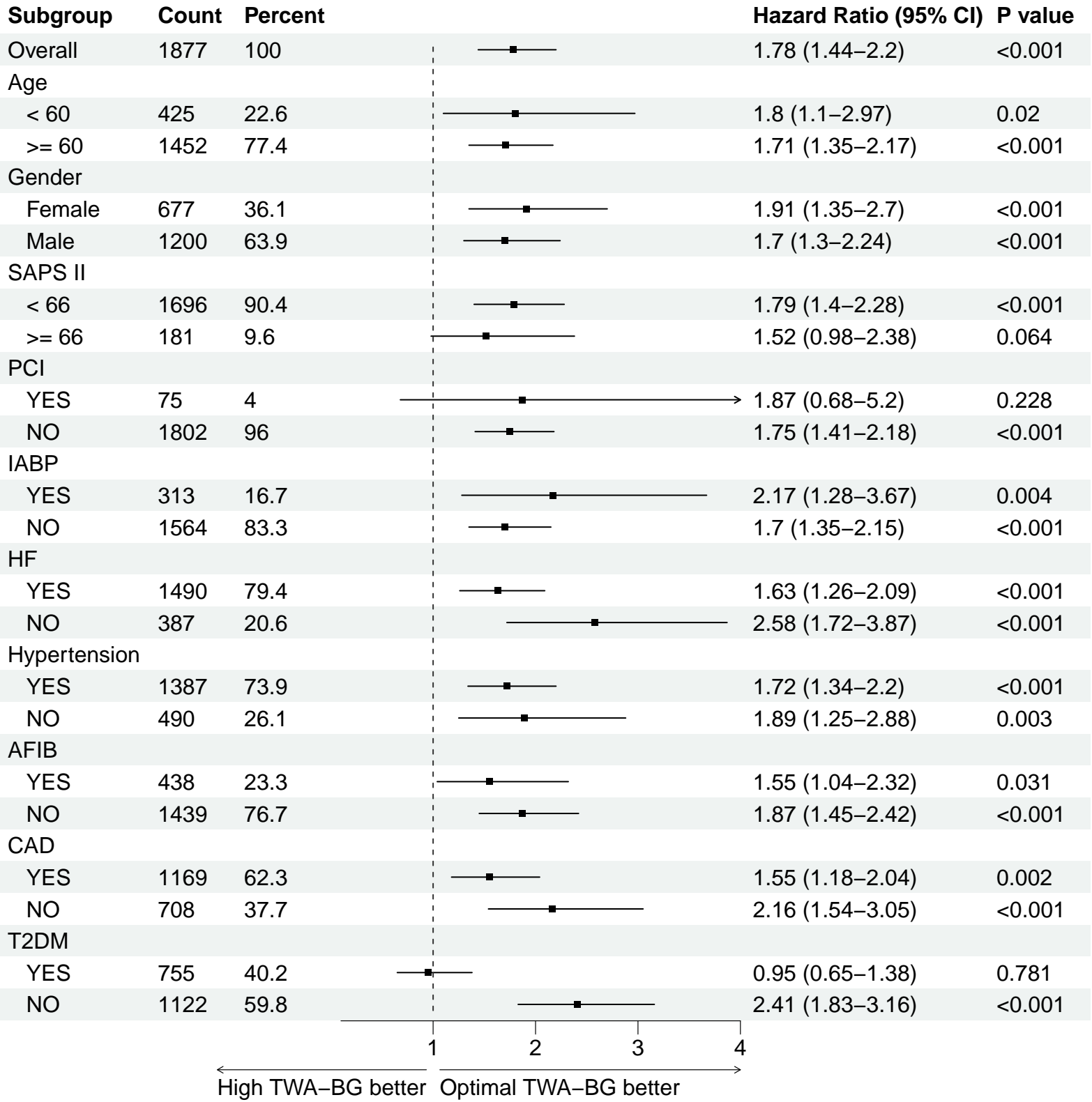

**A**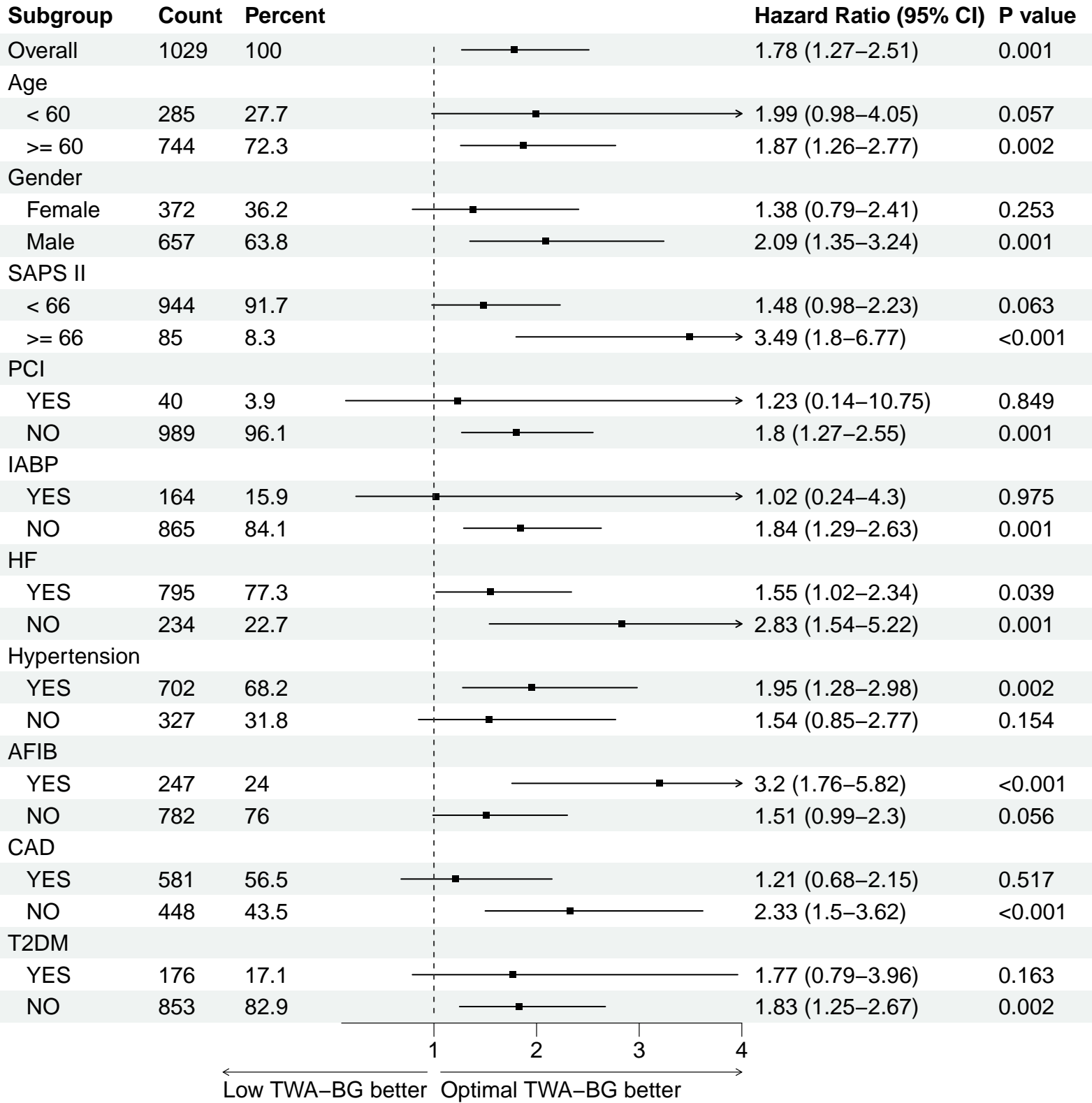**B**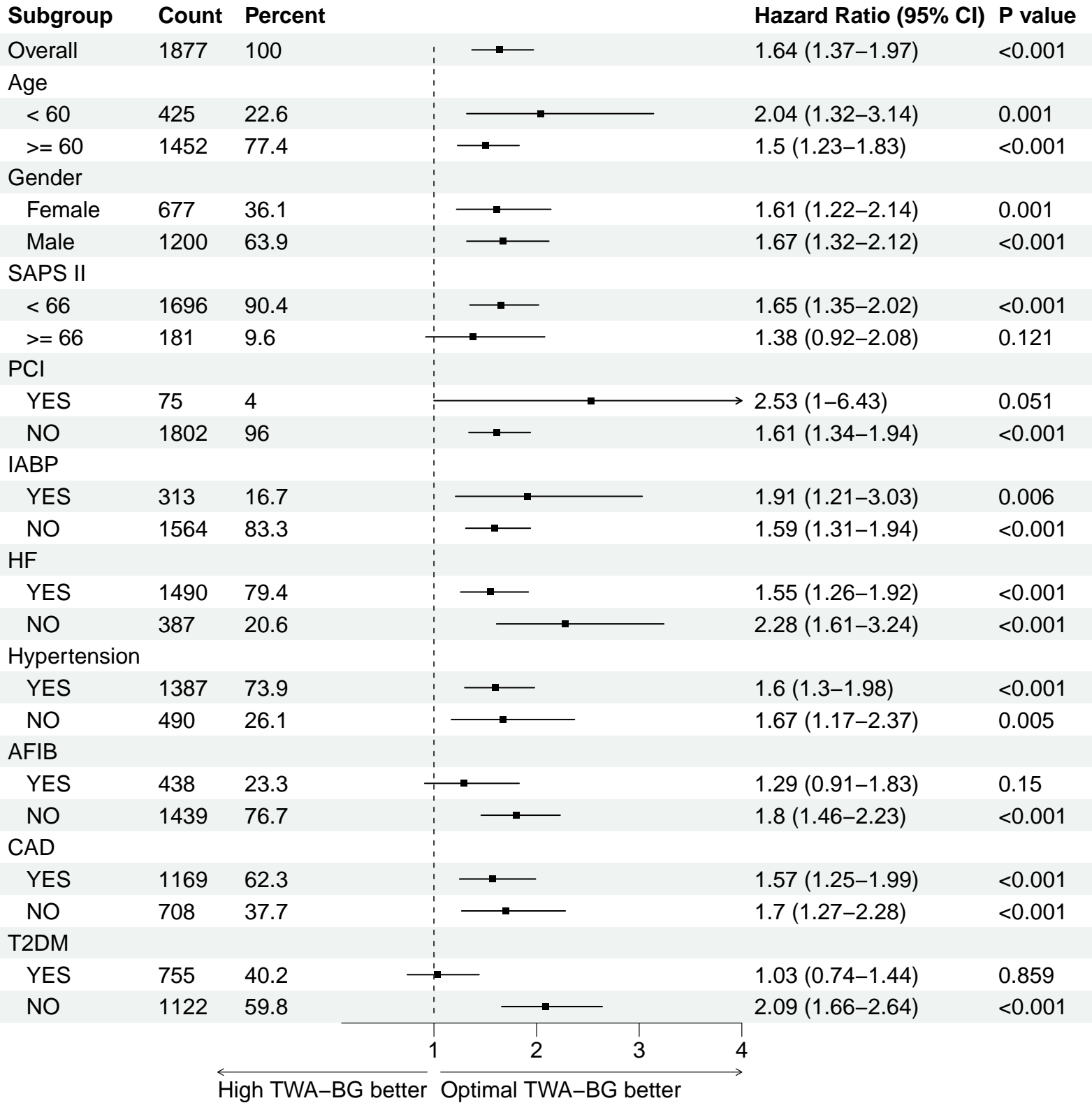

Supplement: Supplementary file 3 — Additional file 3: Fig. S1. TWA-BG trend in ICU for the first 24 h (A), 48 h (B), 72 h (C) and 7 days (D). Fig. S2 Change in SMD before and after matching of cohort 1 (A) and cohort 2 (B). Fig. S3 Forest plot of subgroup analysis for ICU mortality in cohort 1 (A) and cohort 2 (B). Fig. S4 Forest plot of subgroup analysis for in-hospital mortality in cohort 1 (A) and cohort 2 (B). [file 40001_2024_1724_MOESM3_ESM.pdf]
