# Supplementary material for: The optimal blood glucose is significantly associated with lower mortality in critically ill patients with cardiogenic shock: an analysis revealed with time series blood glucose records
Source: Eur J Med Res. 2024 Feb 17;29:129. doi: 10.1186/s40001-024-01724-8 (PMC10874009; doi:10.1186/s40001-024-01724-8)
Supplement: Supplementary file 4 — Additional file 4: Table S1. Basic demographic characteristics of the original cohort. Table S2. Standardized mean difference (SMD) of covariates before and after propensity score matching of cohort 1. Table S3. Baseline characteristics before propensity score matching of cohort 1. Table S4. Baseline characteristics after propensity score matching of cohort 1. Table S5. Standardized mean difference (SMD) of covariates before and after propensity score matching of cohort 2. Table S6. Baseline characteristics before propensity score matching of cohort 2. Table S7. Baseline characteristics after propensity score matching of cohort 2. Table S8. Unadjusted log-rank test for 28-day mortality of original cohort. Table S9. Unadjusted log-rank test for ICU mortality of original cohort. Table S10. Unadjusted log-rank test for in-hospital mortality of original cohort. Table S11. Multivariate Cox model adjusted with all covariates for 28-day mortality of original cohort 1. Table S12. Multivariate Cox model adjusted with unbalanced covariates for 28-day mortality of original cohort 1. Table S13. Multivariate Cox model adjusted with all covariates and IPTW for 28-day mortality of cohort 1. Table S14. Survey-weighted Cox model adjusted with all covariates and IPTW for 28-day mortality of cohort 1. Table S15. Multivariate Cox model adjusted with all covariates for ICU mortality of original cohort 1. Table S16. Multivariate Cox model adjusted with unbalanced covariates for ICU mortality of original cohort 1. Table S17. Multivariate Cox model adjusted with all covariates and IPTW for ICU mortality of cohort 1. Table S18. Survey-weighted Cox model adjusted with all covariates and IPTW for ICU mortality of cohort 1. Table S19. Multivariate Cox model adjusted with all covariates for in-hospital mortality of original cohort 1. Table S20. Multivariate Cox model adjusted with unbalanced covariates for in-hospital mortality of original cohort 1. Table S21. Multivariate Cox model adjusted wi [file 40001_2024_1724_MOESM4_ESM.docx]

## Table S1. Basic demographic characteristics of the original cohort

|  | **Overall (N=2013)** | **Optimal TWA-BG (N=893)** | **Low TWA-BG (N=136)** | **High TWA-BG (N=984)** | **p-value** | **SMD (Compare to Optimal TWA-BG, respectively)** | **Missing data (%)** |
| --- | --- | --- | --- | --- | --- | --- | --- |
| Age | **68.24 (14.32)** | **67.33 (15.16)** | **64.51 (17.69)** | **69.58 (12.78)** | **<0.01** | **[0.171, 0.160]** | **0.00** |
| Gender (Female) | 733 (36.41%) | 316 (35.39%) | 56 (41.18%) | 361 (36.69%) | 0.412 | [0.119, 0.027] | 0.00 |
| Weight | **83.58 (21.93)** | **82.10 (22.03)** | **81.09 (25.65)** | **85.26 (21.17)** | **<0.001** | **[0.042, 0.147]** | **0.05** |
| SAPS II | **44.89 (15.02)** | **43.26 (14.06)** | **42.71 (16.92)** | **46.67 (15.39)** | **<0.001** | **[0.035, 0.231]** | **0.00** |
| SOFA score | 7.71 (3.82) | 7.63 (3.76) | 7.40 (4.30) | 7.83 (3.81) | 0.218 | [0.058, 0.053] | 0.00 |
| Charlson comorbidity index | **6.31 (2.69)** | **5.64 (2.51)** | **5.93 (3.08)** | **6.97 (2.62)** | **<0.001** | **[0.104, 0.518]** | **0.00** |
| **Interventions (boolean for 1st 24 h)** | | | | | | | |
| CABG (YES) | **126 (6.26%)** | **74 (8.29%)** | **4 (2.94%)** | **48 (4.88%)** | **<0.01** | **[0.234, 0.138]** | **0.00** |
| PCI (YES) | 81 (4.02%) | 34 (3.81%) | 6 (4.41%) | 41 (4.17%) | 0.899 | [0.030, 0.018] | 0.00 |
| IABP (YES) | 326 (16.19%) | 151 (16.91%) | 13 (9.56%) | 162 (16.46%) | 0.091 | [0.218, 0.012] | 0.00 |
| PiCCO (YES) | 8 (0.40%) | 2 (0.22%) | 1 (0.74%) | 5 (0.51%) | 0.503 | [0.074, 0.047] | 0.00 |
| NICOM (YES) | **58 (2.88%)** | **16 (1.79%)** | **5 (3.68%)** | **37 (3.76%)** | **<0.05** | **[0.116, 0.120]** | **0.00** |
| Mechanical ventilation (YES) | **1168 (58.02%)** | **545 (61.03%)** | **57 (41.91%)** | **566 (57.52%)** | **<0.001** | **[0.390, 0.071]** | **0.00** |
| Sedative therapy (YES) | **1201 (59.66%)** | **566 (63.38%)** | **59 (43.38%)** | **576 (58.54%)** | **<0.001** | **[0.409, 0.099]** | **0.00** |
| **Comorbidities (boolean)** | | | | | | | |
| HF (YES) | **1599 (79.43%)** | **686 (76.82%)** | **109 (80.15%)** | **804 (81.71%)** | **<0.05** | **[0.081, 0.121]** | **0.00** |
| Hypertension (YES) | **1471 (73.08%)** | **618 (69.20%)** | **84 (61.76%)** | **769 (78.15%)** | **<0.001** | **[0.157, 0.204]** | **0.00** |
| AFIB (YES) | 469 (23.30%) | 216 (24.19%) | 31 (22.79%) | 222 (22.56%) | 0.7 | [0.033, 0.038] | 0.00 |
| T2DM (YES) | **774 (38.45%)** | **157 (17.58%)** | **19 (13.97%)** | **598 (60.77%)** | **<0.001** | **[0.099, 0.987]** | **0.00** |
| Renal (YES) | **781 (38.80%)** | **286 (32.03%)** | **49 (36.03%)** | **446 (45.33%)** | **<0.001** | **[0.085, 0.276]** | **0.00** |
| Liver (YES) | **78 (3.87%)** | **39 (4.37%)** | **10 (7.35%)** | **29 (2.95%)** | **<0.05** | **[0.127, 0.076]** | **0.00** |
| COPD (YES) | 336 (16.69%) | 148 (16.57%) | 26 (19.12%) | 162 (16.46%) | 0.733 | [0.066, 0.003] | 0.00 |
| CAD (YES) | **1231 (61.15%)** | **519 (58.12%)** | **62 (45.59%)** | **650 (66.06%)** | **<0.001** | **[0.253, 0.164]** | **0.00** |
| Stroke (YES) | 183 (9.09%) | 89 (9.97%) | 17 (12.50%) | 77 (7.83%) | 0.098 | [0.080, 0.075] | 0.00 |
| Malignancy (YES) | **226 (11.23%)** | **87 (9.74%)** | **23 (16.91%)** | **116 (11.79%)** | **<0.05** | **[0.212, 0.066]** | **0.00** |
| Cardiomyopathy (YES) | 620 (30.80%) | 286 (32.03%) | 51 (37.50%) | 283 (28.76%) | 0.067 | [0.115, 0.071] | 0.00 |
| HVD (YES) | **890 (44.21%)** | **430 (48.15%)** | **53 (38.97%)** | **407 (41.36%)** | **<0.01** | **[0.186, 0.137]** | **0.00** |
| **Vital signs (1st 24 h)** | | | | | | | |
| MAP | 78.41 (18.71) | 79.16 (17.99) | 77.27 (16.98) | 77.89 (19.55) | 0.109 | [0.108, 0.067] | 0.15 |
| Temperature | 36.49 (0.87) | 36.45 (0.83) | 36.46 (0.89) | 36.53 (0.89) | 0.216 | [0.012, 0.090] | 13.31 |
| Heart rate | **91.14 (20.64)** | **90.69 (20.14)** | **87.17 (21.66)** | **92.10 (20.89)** | **<0.05** | **[0.169, 0.069]** | **0.15** |
| CVP | 15.21 (19.09) | 15.30 (22.51) | 14.95 (8.96) | 15.14 (15.64) | 0.17 | [0.021, 0.008] | 45.11 |
| CO | 4.46 (1.52) | 4.39 (1.59) | 4.47 (1.62) | 4.59 (1.39) | 0.159 | [0.054, 0.135] | 76.75 |
| **Laboratory tests (1st 24 h)** | | | | | | | |
| WBC count | **14.18 (9.29)** | **13.75 (7.07)** | **12.56 (8.74)** | **14.79 (10.95)** | **<0.001** | **[0.150, 0.113]** | **0.40** |
| Hemoglobin | 10.63 (2.47) | 10.64 (2.54) | 10.44 (2.45) | 10.64 (2.42) | 0.501 | [0.080, 0.001] | 0.40 |
| Platelet | **202.92 (101.24)** | **193.73 (100.01)** | **211.40 (110.94)** | **210.07 (100.36)** | **<0.001** | **[0.168, 0.163]** | **0.40** |
| pH | 7.34 (0.11) | 7.35 (0.10) | 7.35 (0.10) | 7.34 (0.11) | 0.157 | [0.034, 0.116] | 13.21 |
| PO2 | **178.10 (124.22)** | **199.96 (130.00)** | **141.82 (101.82)** | **161.51 (117.36)** | **<0.001** | **[0.499, 0.311]** | **31.30** |
| PCO2 | 41.26 (12.98) | 41.19 (12.23) | 43.93 (15.09) | 41.02 (13.40) | 0.427 | [0.200, 0.013] | 31.94 |
| Lactate | **3.16 (2.60)** | **3.08 (2.45)** | **2.74 (2.51)** | **3.30 (2.74)** | **<0.05** | **[0.136, 0.086]** | **10.38** |
| Creatinine | **1.94 (1.55)** | **1.83 (1.69)** | **2.04 (1.38)** | **2.02 (1.43)** | **<0.001** | **[0.136, 0.118]** | **0.05** |
| **Outcomes (boolean)** | | | | | | | |
| 28-day mortality (Death) | **598 (29.71%)** | **204 (22.84%)** | **47 (34.56%)** | **347 (35.26%)** | **<0.001** | **[0.261, 0.276]** | **0.00** |
| ICU mortality (Death) | **401 (19.92%)** | **129 (14.45%)** | **27 (19.85%)** | **245 (24.90%)** | **<0.001** | **[0.144, 0.265]** | **0.00** |
| In-hospital mortality (Death) | **543 (26.97%)** | **186 (20.83%)** | **40 (29.41%)** | **317 (32.22%)** | **<0.001** | **[0.199, 0.260]** | **0.00** |
| **Length of Stay (days)** | | | | | | | |
| ICU LOS | **7.53 (7.94)** | **7.88 (8.25)** | **4.60 (3.86)** | **7.62 (7.99)** | **<0.001** | **[0.510, 0.031]** | **0.00** |
| In-hospital LOS | **14.46 (15.69)** | **15.31 (17.46)** | **11.66 (13.13)** | **14.08 (14.19)** | **<0.001** | **[0.237, 0.077]** | **0.00** |
| Values are presented as mean (standard deviation) for continuous variables and number (percentage) for categorical variables. Variables in bold have p-value < 0.05. | | | | | | | |

## Table S2. Standardized mean difference (SMD) of covariates before and after propensity score matching of cohort 1

| Characteristic | Before matcing | After matcing |
| --- | --- | --- |
| SMD ≤ 0.1 | 15 | 29 |
| SMD > 0.1 | 21 | 7 |
| Total number of covariates | 36 | 36 |

## Table S3. Baseline characteristics before propensity score matching of cohort 1

|  | **Overall (N=1029)** | **Optimal TWA-BG (N=893)** | **Low TWA-BG (N=136)** | **p-value** | **SMD** | **Missing data (%)** |
| --- | --- | --- | --- | --- | --- | --- |
| Age | 66.95 (15.54) | 67.33 (15.16) | 64.51 (17.69) | 0.127 | 0.171 | 0.00 |
| Gender (Female) | 372 (36.15%) | 316 (35.39%) | 56 (41.18%) | 0.225 | 0.119 | 0.00 |
| Weight | 81.94 (22.52) | 82.07 (22.02) | 81.09 (25.65) | 0.26 | 0.041 | 0.00 |
| SAPS II | 43.19 (14.46) | 43.26 (14.06) | 42.71 (16.92) | 0.459 | 0.035 | 0.00 |
| SOFA score | 7.60 (3.83) | 7.63 (3.76) | 7.40 (4.30) | 0.23 | 0.058 | 0.00 |
| Charlson comorbidity index | 5.67 (2.59) | 5.64 (2.51) | 5.93 (3.08) | 0.491 | 0.104 | 0.00 |
| **Interventions (boolean for 1st 24 h)** | | | | | | |
| CABG (YES) | **78 (7.58%)** | **74 (8.29%)** | **4 (2.94%)** | **<0.05** | **0.234** | **0.00** |
| PCI (YES) | 40 (3.89%) | 34 (3.81%) | 6 (4.41%) | 0.919 | 0.03 | 0.00 |
| IABP (YES) | **164 (15.94%)** | **151 (16.91%)** | **13 (9.56%)** | **<0.05** | **0.218** | **0.00** |
| PiCCO (YES) | 3 (0.29%) | 2 (0.22%) | 1 (0.74%) | 0.86 | 0.074 | 0.00 |
| NICOM (YES) | 21 (2.04%) | 16 (1.79%) | 5 (3.68%) | 0.262 | 0.116 | 0.00 |
| Mechanical ventilation (YES) | **602 (58.50%)** | **545 (61.03%)** | **57 (41.91%)** | **<0.001** | **0.39** | **0.00** |
| Sedative therapy (YES) | **625 (60.74%)** | **566 (63.38%)** | **59 (43.38%)** | **<0.001** | **0.409** | **0.00** |
| **Comorbidities (boolean)** | | | | | | |
| HF (YES) | 795 (77.26%) | 686 (76.82%) | 109 (80.15%) | 0.452 | 0.081 | 0.00 |
| Hypertension (YES) | 702 (68.22%) | 618 (69.20%) | 84 (61.76%) | 0.102 | 0.157 | 0.00 |
| AFIB (YES) | 247 (24.00%) | 216 (24.19%) | 31 (22.79%) | 0.805 | 0.033 | 0.00 |
| T2DM (YES) | 176 (17.10%) | 157 (17.58%) | 19 (13.97%) | 0.358 | 0.099 | 0.00 |
| Renal (YES) | 335 (32.56%) | 286 (32.03%) | 49 (36.03%) | 0.407 | 0.085 | 0.00 |
| Liver (YES) | 49 (4.76%) | 39 (4.37%) | 10 (7.35%) | 0.191 | 0.127 | 0.00 |
| COPD (YES) | 174 (16.91%) | 148 (16.57%) | 26 (19.12%) | 0.539 | 0.066 | 0.00 |
| CAD (YES) | **581 (56.46%)** | **519 (58.12%)** | **62 (45.59%)** | **<0.01** | **0.253** | **0.00** |
| Stroke (YES) | 106 (10.30%) | 89 (9.97%) | 17 (12.50%) | 0.451 | 0.08 | 0.00 |
| Malignancy (YES) | **110 (10.69%)** | **87 (9.74%)** | **23 (16.91%)** | **<0.05** | **0.212** | **0.00** |
| Cardiomyopathy (YES) | 337 (32.75%) | 286 (32.03%) | 51 (37.50%) | 0.242 | 0.115 | 0.00 |
| HVD (YES) | 483 (46.94%) | 430 (48.15%) | 53 (38.97%) | 0.057 | 0.186 | 0.00 |
| **Vital signs (1st 24 h)** | | | | | | |
| MAP | 78.89 (17.85) | 79.14 (17.98) | 77.27 (16.98) | 0.293 | 0.107 | 0.00 |
| Temperature | 36.43 (0.87) | 36.43 (0.87) | 36.44 (0.92) | 0.45 | 0.01 | 0.00 |
| Heart rate | 90.20 (20.36) | 90.66 (20.13) | 87.17 (21.66) | 0.087 | 0.167 | 0.00 |
| CVP | 15.27 (21.57) | 15.30 (22.51) | 14.95 (8.96) | 0.644 | 0.021 | 41.50 |
| CO | 4.39 (1.59) | 4.39 (1.59) | 4.47 (1.62) | 0.803 | 0.054 | 71.14 |
| **Laboratory tests (1st 24 h)** | | | | | | |
| WBC count | **13.60 (7.32)** | **13.75 (7.07)** | **12.56 (8.74)** | **<0.001** | **0.15** | **0.00** |
| Hemoglobin | 10.61 (2.52) | 10.64 (2.54) | 10.44 (2.45) | 0.281 | 0.081 | 0.00 |
| Platelet | 196.86 (103.14) | 194.65 (101.79) | 211.40 (110.94) | 0.059 | 0.158 | 0.00 |
| pH | 7.35 (0.10) | 7.35 (0.10) | 7.35 (0.10) | 0.85 | 0.031 | 0.00 |
| PO2 | **185.52 (127.29)** | **189.81 (128.89)** | **157.36 (112.66)** | **<0.05** | **0.269** | **0.00** |
| PCO2 | 40.61 (11.82) | 40.49 (11.67) | 41.39 (12.83) | 0.884 | 0.074 | 0.00 |
| Lactate | **2.98 (2.42)** | **3.03 (2.42)** | **2.67 (2.41)** | **<0.01** | **0.149** | **0.00** |
| Creatinine | **1.86 (1.65)** | **1.83 (1.69)** | **2.04 (1.38)** | **<0.001** | **0.137** | **0.00** |
| **Outcomes (boolean)** | | | | | | |
| 28-day mortality (Death) | **251 (24.39%)** | **204 (22.84%)** | **47 (34.56%)** | **<0.01** | **0.261** | **0.00** |
| ICU mortality (Death) | 156 (15.16%) | 129 (14.45%) | 27 (19.85%) | 0.131 | 0.144 | 0.00 |
| In-hospital mortality (Death) | **226 (21.96%)** | **186 (20.83%)** | **40 (29.41%)** | **<0.05** | **0.199** | **0.00** |
| **Length of Stay (days)** | | | | | | |
| ICU LOS | **7.45 (7.89)** | **7.88 (8.25)** | **4.60 (3.86)** | **<0.001** | **0.51** | **0.00** |
| In-hospital LOS | **14.83 (16.99)** | **15.31 (17.46)** | **11.66 (13.13)** | **<0.001** | **0.237** | **0.00** |
| Values are presented as mean (standard deviation) for continuous variables and number (percentage) for categorical variables. Variables in bold have p-value < 0.05. | | | | | | |

## Table S4. Baseline characteristics after propensity score matching of cohort 1

|  | **Overall (N=264)** | **Optimal TWA-BG (N=132)** | **Low TWA-BG (N=132)** | **p-value** | **SMD** | **Missing data (%)** |
| --- | --- | --- | --- | --- | --- | --- |
| Age | 63.81 (16.72) | 62.67 (15.71) | 64.95 (17.65) | 0.166 | 0.137 | 0.00 |
| Gender (Female) | 108 (40.91%) | 54 (40.91%) | 54 (40.91%) | 1 | <0.001 | 0.00 |
| Weight | 82.61 (25.70) | 83.45 (25.83) | 81.77 (25.64) | 0.531 | 0.065 | 0.00 |
| SAPS II | 42.79 (15.82) | 42.67 (14.51) | 42.90 (17.09) | 0.802 | 0.014 | 0.00 |
| SOFA score | 7.46 (3.98) | 7.53 (3.60) | 7.39 (4.33) | 0.435 | 0.036 | 0.00 |
| Charlson comorbidity index | 6.00 (3.02) | 6.06 (2.93) | 5.94 (3.11) | 0.729 | 0.04 | 0.00 |
| **Interventions (boolean for 1st 24 h)** | | | | | | |
| CABG (YES) | 8 (3.03%) | 4 (3.03%) | 4 (3.03%) | 1 | <0.001 | 0.00 |
| PCI (YES) | 11 (4.17%) | 5 (3.79%) | 6 (4.55%) | 1 | 0.038 | 0.00 |
| IABP (YES) | 23 (8.71%) | 10 (7.58%) | 13 (9.85%) | 0.662 | 0.081 | 0.00 |
| PiCCO (YES) | 2 (0.76%) | 1 (0.76%) | 1 (0.76%) | 1 | <0.001 | 0.00 |
| NICOM (YES) | 5 (1.89%) | 2 (1.52%) | 3 (2.27%) | 1 | 0.056 | 0.00 |
| Mechanical ventilation (YES) | 118 (44.70%) | 62 (46.97%) | 56 (42.42%) | 0.536 | 0.092 | 0.00 |
| Sedative therapy (YES) | 123 (46.59%) | 65 (49.24%) | 58 (43.94%) | 0.459 | 0.106 | 0.00 |
| **Comorbidities (boolean)** | | | | | | |
| HF (YES) | 220 (83.33%) | 114 (86.36%) | 106 (80.30%) | 0.248 | 0.163 | 0.00 |
| Hypertension (YES) | 163 (61.74%) | 82 (62.12%) | 81 (61.36%) | 1 | 0.016 | 0.00 |
| AFIB (YES) | 63 (23.86%) | 33 (25.00%) | 30 (22.73%) | 0.773 | 0.053 | 0.00 |
| T2DM (YES) | 47 (17.80%) | 28 (21.21%) | 19 (14.39%) | 0.198 | 0.179 | 0.00 |
| Renal (YES) | 105 (39.77%) | 57 (43.18%) | 48 (36.36%) | 0.314 | 0.14 | 0.00 |
| Liver (YES) | 21 (7.95%) | 11 (8.33%) | 10 (7.58%) | 1 | 0.028 | 0.00 |
| COPD (YES) | 47 (17.80%) | 22 (16.67%) | 25 (18.94%) | 0.748 | 0.059 | 0.00 |
| CAD (YES) | 126 (47.73%) | 65 (49.24%) | 61 (46.21%) | 0.712 | 0.061 | 0.00 |
| Stroke (YES) | 29 (10.98%) | 15 (11.36%) | 14 (10.61%) | 1 | 0.024 | 0.00 |
| Malignancy (YES) | 48 (18.18%) | 26 (19.70%) | 22 (16.67%) | 0.632 | 0.079 | 0.00 |
| Cardiomyopathy (YES) | 100 (37.88%) | 50 (37.88%) | 50 (37.88%) | 1 | <0.001 | 0.00 |
| HVD (YES) | 99 (37.50%) | 48 (36.36%) | 51 (38.64%) | 0.799 | 0.047 | 0.00 |
| **Vital signs (1st 24 h)** | | | | | | |
| MAP | 77.05 (17.31) | 76.62 (17.58) | 77.48 (17.09) | 0.686 | 0.05 | 0.00 |
| Temperature | 36.42 (0.90) | 36.40 (0.90) | 36.44 (0.90) | 0.611 | 0.048 | 0.00 |
| Heart rate | 86.83 (20.38) | 86.45 (18.73) | 87.22 (21.97) | 0.891 | 0.038 | 0.00 |
| CVP | 15.12 (7.83) | 15.10 (6.82) | 15.14 (9.05) | 0.728 | 0.006 | 51.14 |
| CO | 4.56 (1.48) | 4.67 (1.36) | 4.43 (1.66) | 0.631 | 0.163 | 85.23 |
| **Laboratory tests (1st 24 h)** | | | | | | |
| WBC count | 12.40 (7.83) | 12.12 (6.72) | 12.67 (8.82) | 0.897 | 0.07 | 0.00 |
| Hemoglobin | 10.37 (2.37) | 10.27 (2.28) | 10.47 (2.47) | 0.643 | 0.083 | 0.00 |
| Platelet | 207.97 (109.91) | 205.14 (109.60) | 210.80 (110.57) | 0.514 | 0.052 | 0.00 |
| pH | 7.36 (0.10) | 7.36 (0.11) | 7.35 (0.10) | 0.398 | 0.066 | 0.00 |
| PO2 | 163.19 (116.30) | 168.22 (119.86) | 158.15 (112.85) | 0.624 | 0.087 | 0.00 |
| PCO2 | 40.89 (12.35) | 40.71 (12.23) | 41.06 (12.51) | 0.883 | 0.028 | 0.00 |
| Lactate | 2.83 (2.51) | 2.98 (2.57) | 2.67 (2.44) | 0.175 | 0.126 | 0.00 |
| Creatinine | 2.21 (1.85) | 2.38 (2.20) | 2.03 (1.39) | 0.783 | 0.192 | 0.00 |
| **Outcomes (boolean)** | | | | | | |
| 28-day mortality (Death) | 87 (32.95%) | 42 (31.82%) | 45 (34.09%) | 0.793 | 0.048 | 0.00 |
| ICU mortality (Death) | 48 (18.18%) | 22 (16.67%) | 26 (19.70%) | 0.632 | 0.079 | 0.00 |
| In-hospital mortality (Death) | 75 (28.41%) | 38 (28.79%) | 37 (28.03%) | 1 | 0.017 | 0.00 |
| **Length of Stay (days)** | | | | | | |
| ICU LOS | **5.73 (4.87)** | **6.95 (5.46)** | **4.51 (3.83)** | **<0.001** | **0.519** | **0.00** |
| In-hospital LOS | **13.34 (15.74)** | **15.35 (17.82)** | **11.34 (13.09)** | **<0.001** | **0.257** | **0.00** |
| Values are presented as mean (standard deviation) for continuous variables and number (percentage) for categorical variables. Variables in bold have p-value < 0.05. | | | | | | |

## Table S5. Standardized mean difference (SMD) of covariates before and after propensity score matching of cohort 2

| Characteristic | Before matcing | After matcing |
| --- | --- | --- |
| SMD ≤ 0.1 | 19 | 36 |
| SMD > 0.1 | 17 | 0 |
| Total number of covariates | 36 | 36 |

## Table S6. Baseline characteristics before propensity score matching of cohort 2

|  | **Overall (N=1877)** | **Optimal TWA-BG (N=893)** | **High TWA-BG (N=984)** | **p-value** | **SMD** | **Missing data (%)** |
| --- | --- | --- | --- | --- | --- | --- |
| Age | **68.51 (14.01)** | **67.33 (15.16)** | **69.58 (12.78)** | **<0.05** | **0.16** | **0.00** |
| Gender (Female) | 677 (36.07%) | 316 (35.39%) | 361 (36.69%) | 0.591 | 0.027 | 0.00 |
| Weight | **83.75 (21.63)** | **82.07 (22.02)** | **85.26 (21.17)** | **<0.001** | **0.148** | **0.00** |
| SAPS II | **45.05 (14.86)** | **43.26 (14.06)** | **46.67 (15.39)** | **<0.001** | **0.231** | **0.00** |
| SOFA score | 7.74 (3.79) | 7.63 (3.76) | 7.83 (3.81) | 0.364 | 0.053 | 0.00 |
| Charlson comorbidity index | **6.33 (2.65)** | **5.64 (2.51)** | **6.97 (2.62)** | **<0.001** | **0.518** | **0.00** |
| **Interventions (boolean for 1st 24 h)** | | | | | | |
| CABG (YES) | **122 (6.50%)** | **74 (8.29%)** | **48 (4.88%)** | **<0.01** | **0.138** | **0.00** |
| PCI (YES) | 75 (4.00%) | 34 (3.81%) | 41 (4.17%) | 0.78 | 0.018 | 0.00 |
| IABP (YES) | 313 (16.68%) | 151 (16.91%) | 162 (16.46%) | 0.844 | 0.012 | 0.00 |
| PiCCO (YES) | 7 (0.37%) | 2 (0.22%) | 5 (0.51%) | 0.529 | 0.047 | 0.00 |
| NICOM (YES) | **53 (2.82%)** | **16 (1.79%)** | **37 (3.76%)** | **<0.05** | **0.12** | **0.00** |
| Mechanical ventilation (YES) | 1111 (59.19%) | 545 (61.03%) | 566 (57.52%) | 0.134 | 0.071 | 0.00 |
| Sedative therapy (YES) | **1142 (60.84%)** | **566 (63.38%)** | **576 (58.54%)** | **<0.05** | **0.099** | **0.00** |
| **Comorbidities (boolean)** | | | | | | |
| HF (YES) | **1490 (79.38%)** | **686 (76.82%)** | **804 (81.71%)** | **<0.05** | **0.121** | **0.00** |
| Hypertension (YES) | **1387 (73.89%)** | **618 (69.20%)** | **769 (78.15%)** | **<0.001** | **0.204** | **0.00** |
| AFIB (YES) | 438 (23.34%) | 216 (24.19%) | 222 (22.56%) | 0.437 | 0.038 | 0.00 |
| T2DM (YES) | **755 (40.22%)** | **157 (17.58%)** | **598 (60.77%)** | **<0.001** | **0.987** | **0.00** |
| Renal (YES) | **732 (39.00%)** | **286 (32.03%)** | **446 (45.33%)** | **<0.001** | **0.276** | **0.00** |
| Liver (YES) | 68 (3.62%) | 39 (4.37%) | 29 (2.95%) | 0.128 | 0.076 | 0.00 |
| COPD (YES) | 310 (16.52%) | 148 (16.57%) | 162 (16.46%) | 0.999 | 0.003 | 0.00 |
| CAD (YES) | **1169 (62.28%)** | **519 (58.12%)** | **650 (66.06%)** | **<0.001** | **0.164** | **0.00** |
| Stroke (YES) | 166 (8.84%) | 89 (9.97%) | 77 (7.83%) | 0.121 | 0.075 | 0.00 |
| Malignancy (YES) | 203 (10.82%) | 87 (9.74%) | 116 (11.79%) | 0.177 | 0.066 | 0.00 |
| Cardiomyopathy (YES) | 569 (30.31%) | 286 (32.03%) | 283 (28.76%) | 0.137 | 0.071 | 0.00 |
| HVD (YES) | **837 (44.59%)** | **430 (48.15%)** | **407 (41.36%)** | **<0.01** | **0.137** | **0.00** |
| **Vital signs (1st 24 h)** | | | | | | |
| MAP | **78.49 (18.82)** | **79.14 (17.98)** | **77.90 (19.54)** | **<0.05** | **0.066** | **0.00** |
| Temperature | **36.47 (0.88)** | **36.43 (0.87)** | **36.51 (0.89)** | **<0.05** | **0.098** | **0.00** |
| Heart rate | 91.41 (20.53) | 90.66 (20.13) | 92.10 (20.88) | 0.067 | 0.07 | 0.00 |
| CVP | 15.22 (19.51) | 15.30 (22.51) | 15.14 (15.64) | 0.058 | 0.008 | 44.22 |
| CO | 4.46 (1.52) | 4.39 (1.59) | 4.59 (1.39) | 0.055 | 0.135 | 76.03 |
| **Laboratory tests (1st 24 h)** | | | | | | |
| WBC count | **14.29 (9.31)** | **13.75 (7.07)** | **14.78 (10.93)** | **<0.05** | **0.112** | **0.00** |
| Hemoglobin | 10.64 (2.47) | 10.64 (2.54) | 10.64 (2.42) | 0.941 | <0.001 | 0.00 |
| Platelet | **202.61 (101.30)** | **194.65 (101.79)** | **209.84 (100.35)** | **<0.001** | **0.15** | **0.00** |
| pH | **7.34 (0.11)** | **7.35 (0.10)** | **7.34 (0.11)** | **<0.05** | **0.117** | **0.00** |
| PO2 | **175.00 (123.67)** | **189.81 (128.89)** | **161.55 (117.19)** | **<0.001** | **0.23** | **0.00** |
| PCO2 | 40.59 (12.17) | 40.49 (11.67) | 40.69 (12.61) | 0.895 | 0.017 | 0.00 |
| Lactate | 3.15 (2.55) | 3.03 (2.42) | 3.25 (2.66) | 0.212 | 0.087 | 0.00 |
| Creatinine | **1.93 (1.56)** | **1.83 (1.69)** | **2.02 (1.43)** | **<0.001** | **0.119** | **0.00** |
| **Outcomes (boolean)** | | | | | | |
| 28-day mortality (Death) | **551 (29.36%)** | **204 (22.84%)** | **347 (35.26%)** | **<0.001** | **0.276** | **0.00** |
| ICU mortality (Death) | **374 (19.93%)** | **129 (14.45%)** | **245 (24.90%)** | **<0.001** | **0.265** | **0.00** |
| In-hospital mortality (Death) | **503 (26.80%)** | **186 (20.83%)** | **317 (32.22%)** | **<0.001** | **0.26** | **0.00** |
| **Length of Stay (days)** | | | | | | |
| ICU LOS | 7.75 (8.11) | 7.88 (8.25) | 7.62 (7.99) | 0.202 | 0.031 | 0.00 |
| In-hospital LOS | **14.66 (15.84)** | **15.31 (17.46)** | **14.08 (14.19)** | **<0.01** | **0.077** | **0.00** |
| Values are presented as mean (standard deviation) for continuous variables and number (percentage) for categorical variables. Variables in bold have p-value < 0.05. | | | | | | |

## Table S7. Baseline characteristics after propensity score matching of cohort 2

|  | **Overall (N=1058)** | **Optimal TWA-BG (N=529)** | **High TWA-BG (N=529)** | **p-value** | **SMD** | **Missing data (%)** |
| --- | --- | --- | --- | --- | --- | --- |
| Age | 68.07 (14.93) | 68.44 (15.31) | 67.70 (14.54) | 0.173 | 0.049 | 0.00 |
| Gender (Female) | 385 (36.39%) | 191 (36.11%) | 194 (36.67%) | 0.898 | 0.012 | 0.00 |
| Weight | 83.22 (21.82) | 83.38 (22.29) | 83.05 (21.37) | 0.878 | 0.015 | 0.00 |
| SAPS II | 44.79 (14.93) | 44.94 (14.62) | 44.65 (15.25) | 0.675 | 0.019 | 0.00 |
| SOFA score | 7.57 (3.79) | 7.58 (3.80) | 7.56 (3.78) | 0.804 | 0.005 | 0.00 |
| Charlson comorbidity index | 6.18 (2.56) | 6.27 (2.52) | 6.10 (2.60) | 0.277 | 0.066 | 0.00 |
| **Interventions (boolean for 1st 24 h)** | | | | | | |
| CABG (YES) | 55 (5.20%) | 25 (4.73%) | 30 (5.67%) | 0.58 | 0.043 | 0.00 |
| PCI (YES) | 50 (4.73%) | 26 (4.91%) | 24 (4.54%) | 0.885 | 0.018 | 0.00 |
| IABP (YES) | 188 (17.77%) | 89 (16.82%) | 99 (18.71%) | 0.469 | 0.049 | 0.00 |
| PiCCO (YES) | 4 (0.38%) | 2 (0.38%) | 2 (0.38%) | 1 | <0.001 | 0.00 |
| NICOM (YES) | 27 (2.55%) | 15 (2.84%) | 12 (2.27%) | 0.697 | 0.036 | 0.00 |
| Mechanical ventilation (YES) | 614 (58.03%) | 304 (57.47%) | 310 (58.60%) | 0.755 | 0.023 | 0.00 |
| Sedative therapy (YES) | 637 (60.21%) | 315 (59.55%) | 322 (60.87%) | 0.706 | 0.027 | 0.00 |
| **Comorbidities (boolean)** | | | | | | |
| HF (YES) | 851 (80.43%) | 427 (80.72%) | 424 (80.15%) | 0.877 | 0.014 | 0.00 |
| Hypertension (YES) | 764 (72.21%) | 383 (72.40%) | 381 (72.02%) | 0.945 | 0.008 | 0.00 |
| AFIB (YES) | 255 (24.10%) | 129 (24.39%) | 126 (23.82%) | 0.886 | 0.013 | 0.00 |
| T2DM (YES) | 321 (30.34%) | 156 (29.49%) | 165 (31.19%) | 0.593 | 0.037 | 0.00 |
| Renal (YES) | 393 (37.15%) | 202 (38.19%) | 191 (36.11%) | 0.525 | 0.043 | 0.00 |
| Liver (YES) | 35 (3.31%) | 16 (3.02%) | 19 (3.59%) | 0.731 | 0.032 | 0.00 |
| COPD (YES) | 173 (16.35%) | 84 (15.88%) | 89 (16.82%) | 0.74 | 0.026 | 0.00 |
| CAD (YES) | 646 (61.06%) | 326 (61.63%) | 320 (60.49%) | 0.753 | 0.023 | 0.00 |
| Stroke (YES) | 86 (8.13%) | 45 (8.51%) | 41 (7.75%) | 0.736 | 0.028 | 0.00 |
| Malignancy (YES) | 115 (10.87%) | 61 (11.53%) | 54 (10.21%) | 0.553 | 0.043 | 0.00 |
| Cardiomyopathy (YES) | 332 (31.38%) | 166 (31.38%) | 166 (31.38%) | 1 | <0.001 | 0.00 |
| HVD (YES) | 452 (42.72%) | 228 (43.10%) | 224 (42.34%) | 0.852 | 0.015 | 0.00 |
| **Vital signs (1st 24 h)** | | | | | | |
| MAP | 79.05 (18.64) | 79.04 (16.86) | 79.06 (20.27) | 0.418 | 0.001 | 0.00 |
| Temperature | 36.52 (0.85) | 36.51 (0.80) | 36.53 (0.90) | 0.553 | 0.02 | 0.00 |
| Heart rate | 92.43 (20.70) | 92.29 (20.55) | 92.56 (20.87) | 0.733 | 0.013 | 0.00 |
| CVP | 14.12 (7.03) | 14.18 (7.27) | 14.06 (6.78) | 0.708 | 0.017 | 44.61 |
| CO | 4.50 (1.46) | 4.45 (1.52) | 4.59 (1.37) | 0.292 | 0.092 | 77.32 |
| **Laboratory tests (1st 24 h)** | | | | | | |
| WBC count | 14.21 (7.72) | 14.22 (7.71) | 14.21 (7.74) | 0.964 | 0.002 | 0.00 |
| Hemoglobin | 10.81 (2.52) | 10.82 (2.57) | 10.81 (2.48) | 0.982 | 0.002 | 0.00 |
| Platelet | 205.15 (101.30) | 206.31 (107.18) | 203.99 (95.15) | 0.547 | 0.023 | 0.00 |
| pH | 7.34 (0.11) | 7.34 (0.11) | 7.35 (0.11) | 0.61 | 0.023 | 0.00 |
| PO2 | 172.95 (122.15) | 174.19 (123.94) | 171.71 (120.43) | 0.946 | 0.02 | 0.00 |
| PCO2 | 40.34 (11.86) | 40.22 (11.44) | 40.47 (12.27) | 0.851 | 0.022 | 0.00 |
| Lactate | 3.15 (2.58) | 3.14 (2.56) | 3.16 (2.60) | 0.923 | 0.007 | 0.00 |
| Creatinine | 1.85 (1.45) | 1.86 (1.54) | 1.85 (1.35) | 0.297 | 0.004 | 0.00 |
| **Outcomes (boolean)** | | | | | | |
| 28-day mortality (Death) | **317 (29.96%)** | **132 (24.95%)** | **185 (34.97%)** | **<0.001** | **0.22** | **0.00** |
| ICU mortality (Death) | **215 (20.32%)** | **81 (15.31%)** | **134 (25.33%)** | **<0.001** | **0.251** | **0.00** |
| In-hospital mortality (Death) | **291 (27.50%)** | **120 (22.68%)** | **171 (32.33%)** | **<0.001** | **0.217** | **0.00** |
| **Length of Stay (days)** | | | | | | |
| ICU LOS | 7.77 (8.23) | 7.88 (8.03) | 7.66 (8.44) | 0.12 | 0.026 | 0.00 |
| In-hospital LOS | **15.29 (17.78)** | **16.45 (20.65)** | **14.12 (14.26)** | **<0.01** | **0.131** | **0.00** |
| Values are presented as mean (standard deviation) for continuous variables and number (percentage) for categorical variables. Variables in bold have p-value < 0.05. | | | | | | |

## Table S8. Unadjusted log-rank test for 28-day mortality of original cohort

| Group | HR^1^ | 95% CI^1^ | p-value |
| --- | --- | --- | --- |
| Optimal TWA-BG | — | — | — |
| Low TWA-BG | 1.67 | 1.19, 2.33 | 0.002 |
| High TWA-BG | 1.72 | 1.46, 2.03 | <0.001 |
| ^1^HR = Hazard Ratio, CI = Confidence Interval; | | | |

## Table S9. Unadjusted log-rank test for ICU mortality of original cohort

| Group | HR^1^ | 95% CI^1^ | p-value |
| --- | --- | --- | --- |
| Optimal TWA-BG | — | — | — |
| Low TWA-BG | 2.30 | 1.40, 3.79 | <0.001 |
| High TWA-BG | 1.77 | 1.45, 2.17 | <0.001 |
| ^1^HR = Hazard Ratio, CI = Confidence Interval; | | | |

## Table S10. Unadjusted log-rank test for in-hospital mortality of original cohort

| Group | HR^1^ | 95% CI^1^ | p-value |
| --- | --- | --- | --- |
| Optimal TWA-BG | — | — | — |
| Low TWA-BG | 1.73 | 1.19, 2.51 | 0.001 |
| High TWA-BG | 1.64 | 1.38, 1.95 | <0.001 |
| ^1^HR = Hazard Ratio, CI = Confidence Interval; | | | |

## Table S11. Multivariate Cox model adjusted with all covariates for 28-day mortality of original cohort 1

| **Characteristic** | **HR^1^** | **95% CI^1^** | **p-value** |
| --- | --- | --- | --- |
| Group |  |  |  |
| Optimal TWA-BG | 1.00 | Reference |  |
| Low TWA-BG | 1.58 | 1.12, 2.22 | 0.009 |
| Age | 1.02 | 1.00, 1.03 | 0.034 |
| Gender |  |  |  |
| Female | 1.00 | Reference |  |
| Male | 0.98 | 0.74, 1.32 | 0.913 |
| Weight | 1.00 | 0.99, 1.01 | 0.885 |
| SAPS II (time dependent) | 1.01 | 1.01, 1.01 | <0.001 |
| SOFA score (time dependent) | 1.02 | 1.00, 1.04 | 0.058 |
| Charlson comorbidity index | 1.10 | 1.01, 1.20 | 0.033 |
| CABG |  |  |  |
| YES | 1.00 | Reference |  |
| NO | 2.64 | 1.23, 5.65 | 0.012 |
| PCI |  |  |  |
| YES | 1.00 | Reference |  |
| NO | 1.25 | 0.57, 2.77 | 0.581 |
| IABP |  |  |  |
| YES | 1.00 | Reference |  |
| NO | 0.95 | 0.63, 1.44 | 0.818 |
| PiCCO (time dependent) |  |  |  |
| YES | 1.00 | Reference |  |
| NO | 1.02 | 0.64, 1.64 | 0.919 |
| NICOM (time dependent) |  |  |  |
| YES | 1.00 | Reference |  |
| NO | 0.87 | 0.70, 1.08 | 0.198 |
| Mechanical ventilation |  |  |  |
| YES | 1.00 | Reference |  |
| NO | 1.31 | 0.51, 3.37 | 0.581 |
| Sedative therapy |  |  |  |
| YES | 1.00 | Reference |  |
| NO | 1.71 | 0.66, 4.42 | 0.267 |
| HF (time dependent) |  |  |  |
| YES | 1.00 | Reference |  |
| NO | 1.07 | 0.97, 1.18 | 0.202 |
| Hypertension |  |  |  |
| YES | 1.00 | Reference |  |
| NO | 1.21 | 0.90, 1.63 | 0.204 |
| AFIB |  |  |  |
| YES | 1.00 | Reference |  |
| NO | 0.96 | 0.71, 1.30 | 0.786 |
| T2DM (time dependent) |  |  |  |
| YES | 1.00 | Reference |  |
| NO | 0.93 | 0.84, 1.03 | 0.181 |
| Renal |  |  |  |
| YES | 1.00 | Reference |  |
| NO | 1.47 | 1.02, 2.12 | 0.039 |
| Liver |  |  |  |
| YES | 1.00 | Reference |  |
| NO | 0.75 | 0.45, 1.25 | 0.272 |
| COPD (time dependent) |  |  |  |
| YES | 1.00 | Reference |  |
| NO | 0.93 | 0.84, 1.03 | 0.157 |
| CAD |  |  |  |
| YES | 1.00 | Reference |  |
| NO | 0.99 | 0.73, 1.34 | 0.945 |
| Stroke |  |  |  |
| YES | 1.00 | Reference |  |
| NO | 1.04 | 0.69, 1.58 | 0.845 |
| Malignancy |  |  |  |
| YES | 1.00 | Reference |  |
| NO | 1.00 | 0.65, 1.54 | 0.998 |
| Cardiomyopathy (time dependent) |  |  |  |
| YES | 1.00 | Reference |  |
| NO | 0.92 | 0.85, 1.01 | 0.068 |
| HVD |  |  |  |
| YES | 1.00 | Reference |  |
| NO | 1.47 | 1.11, 1.95 | 0.007 |
| MAP | 0.99 | 0.98, 1.00 | 0.002 |
| Temperature | 0.93 | 0.81, 1.07 | 0.325 |
| Heart rate | 1.01 | 1.00, 1.01 | 0.036 |
| WBC count | 1.01 | 1.00, 1.03 | 0.148 |
| Hemoglobin | 0.93 | 0.88, 0.99 | 0.025 |
| Platelet | 1.00 | 1.00, 1.00 | 0.421 |
| pH | 0.40 | 0.08, 1.89 | 0.247 |
| PO2 | 1.00 | 1.00, 1.00 | 0.208 |
| PCO2 | 1.00 | 0.99, 1.01 | 0.872 |
| Lactate | 1.03 | 0.98, 1.08 | 0.3 |
| Creatinine | 1.09 | 1.01, 1.18 | 0.021 |
| ^1^HR = Hazard Ratio, CI = Confidence Interval | | | |

## Table S12. Multivariate Cox model adjusted with unbalanced covariates for 28-day mortality of original cohort 1

| **Characteristic** | **HR^1^** | **95% CI^1^** | **p-value** |
| --- | --- | --- | --- |
| Group |  |  |  |
| Optimal TWA-BG | 1.00 | Reference |  |
| Low TWA-BG | 1.61 | 1.16, 2.25 | 0.005 |
| Age | 1.03 | 1.02, 1.04 | <0.001 |
| Gender |  |  |  |
| Female | 1.00 | Reference |  |
| Male | 0.91 | 0.69, 1.19 | 0.478 |
| Charlson comorbidity index | 1.10 | 1.03, 1.18 | 0.004 |
| CABG |  |  |  |
| YES | 1.00 | Reference |  |
| NO | 2.43 | 1.16, 5.06 | 0.018 |
| IABP |  |  |  |
| YES | 1.00 | Reference |  |
| NO | 1.01 | 0.68, 1.51 | 0.952 |
| NICOM (time dependent) |  |  |  |
| YES | 1.00 | Reference |  |
| NO | 0.89 | 0.72, 1.09 | 0.265 |
| Mechanical ventilation |  |  |  |
| YES | 1.00 | Reference |  |
| NO | 0.74 | 0.33, 1.67 | 0.465 |
| Sedative therapy |  |  |  |
| YES | 1.00 | Reference |  |
| NO | 1.22 | 0.54, 2.77 | 0.637 |
| Hypertension |  |  |  |
| YES | 1.00 | Reference |  |
| NO | 1.22 | 0.91, 1.64 | 0.181 |
| Liver |  |  |  |
| YES | 1.00 | Reference |  |
| NO | 0.59 | 0.36, 0.96 | 0.033 |
| CAD |  |  |  |
| YES | 1.00 | Reference |  |
| NO | 1.15 | 0.86, 1.53 | 0.337 |
| Malignancy |  |  |  |
| YES | 1.00 | Reference |  |
| NO | 0.87 | 0.59, 1.28 | 0.484 |
| Cardiomyopathy (time dependent) |  |  |  |
| YES | 1.00 | Reference |  |
| NO | 0.95 | 0.88, 1.04 | 0.276 |
| HVD |  |  |  |
| YES | 1.00 | Reference |  |
| NO | 1.50 | 1.14, 1.97 | 0.004 |
| MAP | 0.99 | 0.98, 0.99 | <0.001 |
| Heart rate | 1.01 | 1.00, 1.02 | 0.001 |
| WBC count | 1.02 | 1.00, 1.03 | 0.057 |
| Platelet | 1.00 | 1.00, 1.00 | 0.094 |
| PO2 | 1.00 | 1.00, 1.00 | 0.056 |
| Lactate | 1.11 | 1.07, 1.15 | <0.001 |
| Creatinine | 1.14 | 1.07, 1.22 | <0.001 |
| ^1^HR = Hazard Ratio, CI = Confidence Interval | | | |

## Table S13. Multivariate Cox model adjusted with all covariates and IPTW for 28-day mortality of cohort 1

| **Characteristic** | **HR^1^** | **95% CI^1^** | **p-value** |
| --- | --- | --- | --- |
| Group |  |  |  |
| Optimal TWA-BG | 1.00 | Reference |  |
| Low TWA-BG | 1.89 | 1.16, 3.07 | 0.01 |
| Age | 1.02 | 1.00, 1.04 | 0.122 |
| Gender |  |  |  |
| Female | 1.00 | Reference |  |
| Male | 1.29 | 0.80, 2.08 | 0.296 |
| Weight | 1.00 | 0.99, 1.01 | 0.665 |
| SAPS II (time dependent) | 1.01 | 1.01, 1.02 | <0.001 |
| SOFA score (time dependent) | 1.04 | 1.02, 1.06 | 0.001 |
| Charlson comorbidity index (time dependent) | 1.02 | 0.99, 1.06 | 0.232 |
| CABG |  |  |  |
| YES | 1.00 | Reference |  |
| NO | 11.18 | 3.59, 34.81 | <0.001 |
| PCI |  |  |  |
| YES | 1.00 | Reference |  |
| NO | 0.93 | 0.26, 3.38 | 0.915 |
| IABP |  |  |  |
| YES | 1.00 | Reference |  |
| NO | 1.16 | 0.66, 2.04 | 0.596 |
| PiCCO (time dependent) |  |  |  |
| YES | 1.00 | Reference |  |
| NO | 1.55 | 0.78, 3.07 | 0.214 |
| NICOM (time dependent) |  |  |  |
| YES | 1.00 | Reference |  |
| NO | 0.81 | 0.54, 1.20 | 0.295 |
| Mechanical ventilation (time dependent) |  |  |  |
| YES | 1.00 | Reference |  |
| NO | 1.47 | 0.91, 2.39 | 0.119 |
| Sedative therapy (time dependent) |  |  |  |
| YES | 1.00 | Reference |  |
| NO | 0.98 | 0.60, 1.60 | 0.938 |
| HF (time dependent) |  |  |  |
| YES | 1.00 | Reference |  |
| NO | 1.21 | 1.00, 1.46 | 0.048 |
| Hypertension |  |  |  |
| YES | 1.00 | Reference |  |
| NO | 1.25 | 0.78, 2.00 | 0.358 |
| AFIB |  |  |  |
| YES | 1.00 | Reference |  |
| NO | 1.07 | 0.64, 1.77 | 0.804 |
| T2DM |  |  |  |
| YES | 1.00 | Reference |  |
| NO | 0.70 | 0.43, 1.14 | 0.153 |
| Renal (time dependent) |  |  |  |
| YES | 1.00 | Reference |  |
| NO | 1.12 | 0.96, 1.29 | 0.146 |
| Liver |  |  |  |
| YES | 1.00 | Reference |  |
| NO | 0.86 | 0.44, 1.70 | 0.665 |
| COPD (time dependent) |  |  |  |
| YES | 1.00 | Reference |  |
| NO | 0.88 | 0.75, 1.03 | 0.119 |
| CAD |  |  |  |
| YES | 1.00 | Reference |  |
| NO | 1.00 | 0.62, 1.62 | 0.997 |
| Stroke |  |  |  |
| YES | 1.00 | Reference |  |
| NO | 1.20 | 0.59, 2.46 | 0.612 |
| Malignancy (time dependent) |  |  |  |
| YES | 1.00 | Reference |  |
| NO | 1.04 | 0.85, 1.28 | 0.687 |
| Cardiomyopathy (time dependent) |  |  |  |
| YES | 1.00 | Reference |  |
| NO | 0.87 | 0.76, 1.01 | 0.059 |
| HVD (time dependent) |  |  |  |
| YES | 1.00 | Reference |  |
| NO | 1.19 | 1.05, 1.35 | 0.008 |
| MAP (time dependent) | 1.00 | 0.99, 1.00 | 0.074 |
| Temperature (time dependent) | 0.97 | 0.90, 1.04 | 0.342 |
| Heart rate | 1.01 | 1.00, 1.02 | 0.035 |
| WBC count (time dependent) | 1.01 | 1.00, 1.02 | 0.079 |
| Hemoglobin (time dependent) | 0.98 | 0.95, 1.02 | 0.264 |
| Platelet (time dependent) | 1.00 | 1.00, 1.00 | 0.839 |
| pH | 2.00 | 0.16, 24.20 | 0.587 |
| PO2 | 1.00 | 1.00, 1.00 | 0.906 |
| PCO2 | 1.00 | 0.99, 1.02 | 0.655 |
| Lactate | 1.03 | 0.96, 1.12 | 0.411 |
| Creatinine | 1.07 | 0.96, 1.20 | 0.22 |
| ^1^HR = Hazard Ratio, CI = Confidence Interval | | | |

## Table S14. Survey-weighted Cox model adjusted with all covariates and IPTW for 28-day mortality of cohort 1

| **Characteristic** | **HR^1^** | **95% CI^1^** | **p-value** |
| --- | --- | --- | --- |
| Group |  |  |  |
| Optimal TWA-BG | 1.00 | Reference |  |
| Low TWA-BG | 1.91 | 1.17, 3.12 | 0.009 |
| Age | 1.02 | 1.00, 1.04 | 0.106 |
| Gender |  |  |  |
| Female | 1.00 | Reference |  |
| Male | 1.28 | 0.79, 2.08 | 0.313 |
| Weight | 1.00 | 0.99, 1.01 | 0.654 |
| SAPS II (time dependent) | 1.01 | 1.01, 1.02 | <0.001 |
| SOFA score (time dependent) | 1.04 | 1.01, 1.06 | 0.002 |
| Charlson comorbidity index | 1.08 | 0.95, 1.23 | 0.238 |
| CABG |  |  |  |
| YES | 1.00 | Reference |  |
| NO | 11.42 | 3.61, 36.14 | <0.001 |
| PCI |  |  |  |
| YES | 1.00 | Reference |  |
| NO | 0.94 | 0.26, 3.34 | 0.922 |
| IABP |  |  |  |
| YES | 1.00 | Reference |  |
| NO | 1.16 | 0.66, 2.02 | 0.61 |
| PiCCO (time dependent) |  |  |  |
| YES | 1.00 | Reference |  |
| NO | 1.61 | 0.79, 3.27 | 0.191 |
| NICOM (time dependent) |  |  |  |
| YES | 1.00 | Reference |  |
| NO | 0.82 | 0.55, 1.22 | 0.326 |
| Mechanical ventilation |  |  |  |
| YES | 1.00 | Reference |  |
| NO | 3.30 | 0.62, 17.53 | 0.162 |
| Sedative therapy |  |  |  |
| YES | 1.00 | Reference |  |
| NO | 0.98 | 0.18, 5.30 | 0.98 |
| HF |  |  |  |
| YES | 1.00 | Reference |  |
| NO | 2.00 | 1.07, 3.76 | 0.031 |
| Hypertension |  |  |  |
| YES | 1.00 | Reference |  |
| NO | 1.26 | 0.78, 2.02 | 0.34 |
| AFIB |  |  |  |
| YES | 1.00 | Reference |  |
| NO | 1.09 | 0.65, 1.81 | 0.753 |
| T2DM (time dependent) |  |  |  |
| YES | 1.00 | Reference |  |
| NO | 0.90 | 0.78, 1.05 | 0.189 |
| Renal |  |  |  |
| YES | 1.00 | Reference |  |
| NO | 1.43 | 0.86, 2.38 | 0.165 |
| Liver |  |  |  |
| YES | 1.00 | Reference |  |
| NO | 0.85 | 0.43, 1.67 | 0.636 |
| COPD (time dependent) |  |  |  |
| YES | 1.00 | Reference |  |
| NO | 0.88 | 0.75, 1.03 | 0.116 |
| CAD |  |  |  |
| YES | 1.00 | Reference |  |
| NO | 1.00 | 0.62, 1.60 | 0.997 |
| Stroke |  |  |  |
| YES | 1.00 | Reference |  |
| NO | 1.20 | 0.59, 2.45 | 0.608 |
| Malignancy |  |  |  |
| YES | 1.00 | Reference |  |
| NO | 1.18 | 0.60, 2.34 | 0.627 |
| Cardiomyopathy (time dependent) |  |  |  |
| YES | 1.00 | Reference |  |
| NO | 0.87 | 0.76, 1.00 | 0.055 |
| HVD |  |  |  |
| YES | 1.00 | Reference |  |
| NO | 1.83 | 1.19, 2.82 | 0.006 |
| MAP | 0.99 | 0.98, 1.00 | 0.047 |
| Temperature | 0.91 | 0.72, 1.15 | 0.425 |
| Heart rate | 1.01 | 1.00, 1.02 | 0.031 |
| WBC count | 1.02 | 1.00, 1.05 | 0.103 |
| Hemoglobin | 0.94 | 0.83, 1.07 | 0.343 |
| Platelet | 1.00 | 1.00, 1.00 | 0.712 |
| pH | 2.05 | 0.17, 24.30 | 0.569 |
| PO2 | 1.00 | 1.00, 1.00 | 0.909 |
| PCO2 | 1.00 | 0.99, 1.02 | 0.638 |
| Lactate | 1.03 | 0.96, 1.12 | 0.389 |
| Creatinine | 1.08 | 0.96, 1.21 | 0.182 |
| ^1^HR = Hazard Ratio, CI = Confidence Interval | | | |

## Table S15. Multivariate Cox model adjusted with all covariates for ICU mortality of original cohort 1

| **Characteristic** | **HR^1^** | **95% CI^1^** | **p-value** |
| --- | --- | --- | --- |
| Group |  |  |  |
| Optimal TWA-BG | 1.00 | Reference |  |
| Low TWA-BG | 2.29 | 1.41, 3.73 | <0.001 |
| Age | 1.01 | 0.99, 1.03 | 0.244 |
| Gender |  |  |  |
| Female | 1.00 | Reference |  |
| Male | 1.16 | 0.78, 1.71 | 0.461 |
| Weight | 0.99 | 0.99, 1.00 | 0.19 |
| SAPS II (time dependent) | 1.01 | 1.01, 1.02 | <0.001 |
| SOFA score (time dependent) | 1.02 | 0.99, 1.04 | 0.172 |
| Charlson comorbidity index | 1.11 | 0.98, 1.25 | 0.088 |
| CABG |  |  |  |
| YES | 1.00 | Reference |  |
| NO | 2.67 | 1.20, 5.93 | 0.016 |
| PCI |  |  |  |
| YES | 1.00 | Reference |  |
| NO | 0.63 | 0.26, 1.53 | 0.303 |
| IABP |  |  |  |
| YES | 1.00 | Reference |  |
| NO | 0.94 | 0.57, 1.55 | 0.81 |
| PiCCO |  |  |  |
| YES | 1.00 | Reference |  |
| NO | 3.34 | 0.35, 32.16 | 0.296 |
| NICOM (time dependent) |  |  |  |
| YES | 1.00 | Reference |  |
| NO | 0.82 | 0.65, 1.05 | 0.122 |
| Mechanical ventilation |  |  |  |
| YES | 1.00 | Reference |  |
| NO | 1.09 | 0.25, 4.71 | 0.911 |
| Sedative therapy |  |  |  |
| YES | 1.00 | Reference |  |
| NO | 1.90 | 0.44, 8.26 | 0.389 |
| HF |  |  |  |
| YES | 1.00 | Reference |  |
| NO | 1.17 | 0.75, 1.82 | 0.489 |
| Hypertension |  |  |  |
| YES | 1.00 | Reference |  |
| NO | 1.12 | 0.75, 1.66 | 0.578 |
| AFIB |  |  |  |
| YES | 1.00 | Reference |  |
| NO | 1.52 | 1.00, 2.31 | 0.052 |
| T2DM |  |  |  |
| YES | 1.00 | Reference |  |
| NO | 0.51 | 0.33, 0.80 | 0.003 |
| Renal |  |  |  |
| YES | 1.00 | Reference |  |
| NO | 2.26 | 1.35, 3.77 | 0.002 |
| Liver |  |  |  |
| YES | 1.00 | Reference |  |
| NO | 0.64 | 0.35, 1.18 | 0.151 |
| COPD (time dependent) |  |  |  |
| YES | 1.00 | Reference |  |
| NO | 0.88 | 0.77, 1.01 | 0.06 |
| CAD |  |  |  |
| YES | 1.00 | Reference |  |
| NO | 0.83 | 0.56, 1.23 | 0.359 |
| Stroke |  |  |  |
| YES | 1.00 | Reference |  |
| NO | 1.61 | 0.95, 2.75 | 0.079 |
| Malignancy |  |  |  |
| YES | 1.00 | Reference |  |
| NO | 1.34 | 0.71, 2.53 | 0.368 |
| Cardiomyopathy |  |  |  |
| YES | 1.00 | Reference |  |
| NO | 1.19 | 0.78, 1.81 | 0.431 |
| HVD |  |  |  |
| YES | 1.00 | Reference |  |
| NO | 1.32 | 0.91, 1.93 | 0.144 |
| MAP | 0.98 | 0.97, 0.99 | 0.003 |
| Temperature | 1.01 | 0.84, 1.21 | 0.901 |
| Heart rate (time dependent) | 1.00 | 1.00, 1.00 | 0.586 |
| WBC count (time dependent) | 1.01 | 1.00, 1.02 | 0.007 |
| Hemoglobin | 0.98 | 0.90, 1.06 | 0.557 |
| Platelet | 1.00 | 1.00, 1.00 | 0.414 |
| pH (time dependent) | 0.95 | 0.50, 1.81 | 0.881 |
| PO2 | 1.00 | 1.00, 1.00 | 0.582 |
| PCO2 | 1.00 | 0.98, 1.02 | 0.983 |
| Lactate | 1.00 | 0.94, 1.06 | 0.995 |
| Creatinine | 1.22 | 1.10, 1.35 | <0.001 |
| ^1^HR = Hazard Ratio, CI = Confidence Interval | | | |

## Table S16. Multivariate Cox model adjusted with unbalanced covariates for ICU mortality of original cohort 1

| **Characteristic** | **HR^1^** | **95% CI^1^** | **p-value** |
| --- | --- | --- | --- |
| Group |  |  |  |
| Optimal TWA-BG | 1.00 | Reference |  |
| Low TWA-BG | 2.30 | 1.46, 3.62 | <0.001 |
| Age | 1.03 | 1.01, 1.04 | <0.001 |
| Gender |  |  |  |
| Female | 1.00 | Reference |  |
| Male | 1.00 | 0.71, 1.42 | 0.978 |
| Charlson comorbidity index | 1.07 | 0.98, 1.16 | 0.131 |
| CABG |  |  |  |
| YES | 1.00 | Reference |  |
| NO | 2.05 | 1.00, 4.21 | 0.05 |
| IABP |  |  |  |
| YES | 1.00 | Reference |  |
| NO | 1.09 | 0.67, 1.78 | 0.716 |
| NICOM (time dependent) |  |  |  |
| YES | 1.00 | Reference |  |
| NO | 0.85 | 0.68, 1.05 | 0.132 |
| Mechanical ventilation |  |  |  |
| YES | 1.00 | Reference |  |
| NO | 0.71 | 0.22, 2.29 | 0.569 |
| Sedative therapy |  |  |  |
| YES | 1.00 | Reference |  |
| NO | 1.22 | 0.37, 3.97 | 0.745 |
| Hypertension |  |  |  |
| YES | 1.00 | Reference |  |
| NO | 1.20 | 0.82, 1.75 | 0.344 |
| Liver |  |  |  |
| YES | 1.00 | Reference |  |
| NO | 0.59 | 0.33, 1.07 | 0.084 |
| CAD |  |  |  |
| YES | 1.00 | Reference |  |
| NO | 0.89 | 0.61, 1.28 | 0.521 |
| Malignancy |  |  |  |
| YES | 1.00 | Reference |  |
| NO | 1.13 | 0.66, 1.94 | 0.657 |
| Cardiomyopathy |  |  |  |
| YES | 1.00 | Reference |  |
| NO | 1.23 | 0.82, 1.84 | 0.321 |
| HVD |  |  |  |
| YES | 1.00 | Reference |  |
| NO | 1.62 | 1.13, 2.33 | 0.009 |
| MAP | 0.98 | 0.98, 0.99 | 0.002 |
| Heart rate (time dependent) | 1.00 | 1.00, 1.00 | 0.075 |
| WBC count (time dependent) | 1.01 | 1.00, 1.01 | 0.047 |
| Platelet | 1.00 | 1.00, 1.00 | 0.148 |
| PO2 | 1.00 | 1.00, 1.00 | 0.228 |
| Lactate | 1.09 | 1.04, 1.14 | <0.001 |
| Creatinine | 1.21 | 1.11, 1.31 | <0.001 |
| ^1^HR = Hazard Ratio, CI = Confidence Interval | | | |

## Table S17. Multivariate Cox model adjusted with all covariates and IPTW for ICU mortality of cohort 1

| **Characteristic** | **HR^1^** | **95% CI^1^** | **p-value** |
| --- | --- | --- | --- |
| Group (time dependent) |  |  |  |
| Optimal TWA-BG | 1.00 | Reference |  |
| Low TWA-BG | 1.38 | 1.12, 1.71 | 0.003 |
| Age (time dependent) | 1.00 | 1.00, 1.01 | 0.428 |
| Gender |  |  |  |
| Female | 1.00 | Reference |  |
| Male | 1.15 | 0.61, 2.18 | 0.671 |
| Weight | 1.00 | 0.99, 1.01 | 0.681 |
| SAPS II (time dependent) | 1.01 | 1.00, 1.02 | 0.003 |
| SOFA score (time dependent) | 1.04 | 1.01, 1.08 | 0.023 |
| Charlson comorbidity index | 1.10 | 0.92, 1.32 | 0.290 |
| CABG (time dependent) |  |  |  |
| YES | 1.00 | Reference |  |
| NO | 1.46 | 1.09, 1.97 | 0.012 |
| PCI (time dependent) |  |  |  |
| YES | 1.00 | Reference |  |
| NO | 0.77 | 0.52, 1.14 | 0.194 |
| IABP (time dependent) |  |  |  |
| YES | 1.00 | Reference |  |
| NO | 0.98 | 0.81, 1.20 | 0.873 |
| PiCCO (time dependent) |  |  |  |
| YES | 1.00 | Reference |  |
| NO | 1.71 | 0.74, 3.92 | 0.208 |
| NICOM (time dependent) |  |  |  |
| YES | 1.00 | Reference |  |
| NO | 0.79 | 0.51, 1.23 | 0.301 |
| Mechanical ventilation |  |  |  |
| YES | 1.00 | Reference |  |
| NO | 0.84 | 0.09, 7.72 | 0.876 |
| Sedative therapy |  |  |  |
| YES | 1.00 | Reference |  |
| NO | 3.90 | 0.39, 38.70 | 0.245 |
| HF (time dependent) |  |  |  |
| YES | 1.00 | Reference |  |
| NO | 1.14 | 0.89, 1.46 | 0.296 |
| Hypertension |  |  |  |
| YES | 1.00 | Reference |  |
| NO | 0.98 | 0.53, 1.82 | 0.953 |
| AFIB |  |  |  |
| YES | 1.00 | Reference |  |
| NO | 1.21 | 0.57, 2.56 | 0.617 |
| T2DM |  |  |  |
| YES | 1.00 | Reference |  |
| NO | 0.56 | 0.34, 0.93 | 0.024 |
| Renal |  |  |  |
| YES | 1.00 | Reference |  |
| NO | 2.85 | 1.45, 5.58 | 0.002 |
| Liver |  |  |  |
| YES | 1.00 | Reference |  |
| NO | 0.70 | 0.34, 1.46 | 0.343 |
| COPD (time dependent) |  |  |  |
| YES | 1.00 | Reference |  |
| NO | 0.82 | 0.66, 1.02 | 0.069 |
| CAD |  |  |  |
| YES | 1.00 | Reference |  |
| NO | 0.87 | 0.47, 1.61 | 0.656 |
| Stroke |  |  |  |
| YES | 1.00 | Reference |  |
| NO | 1.75 | 0.74, 4.14 | 0.206 |
| Malignancy |  |  |  |
| YES | 1.00 | Reference |  |
| NO | 1.76 | 0.65, 4.71 | 0.264 |
| Cardiomyopathy |  |  |  |
| YES | 1.00 | Reference |  |
| NO | 1.42 | 0.81, 2.48 | 0.216 |
| HVD (time dependent) |  |  |  |
| YES | 1.00 | Reference |  |
| NO | 1.08 | 0.91, 1.27 | 0.372 |
| MAP (time dependent) | 0.99 | 0.99, 1.00 | 0.002 |
| Temperature | 1.07 | 0.81, 1.42 | 0.629 |
| Heart rate (time dependent) | 1.00 | 1.00, 1.01 | 0.261 |
| WBC count (time dependent) | 1.01 | 1.00, 1.02 | 0.246 |
| Hemoglobin (time dependent) | 1.01 | 0.97, 1.06 | 0.609 |
| Platelet | 1.00 | 1.00, 1.00 | 0.528 |
| pH | 2.73 | 0.08, 93.88 | 0.578 |
| PO2 (time dependent) | 1.00 | 1.00, 1.00 | 0.471 |
| PCO2 (time dependent) | 1.00 | 0.99, 1.01 | 0.738 |
| Lactate | 0.99 | 0.90, 1.08 | 0.780 |
| Creatinine | 1.19 | 1.05, 1.35 | 0.008 |
| ^1^HR = Hazard Ratio, CI = Confidence Interval | | | |

## Table S18. Survey-weighted Cox model adjusted with all covariates and IPTW for ICU mortality of cohort 1

| **Characteristic** | **HR^1^** | **95% CI^1^** | **p-value** |
| --- | --- | --- | --- |
| Group |  |  |  |
| Optimal TWA-BG | 1.00 | Reference |  |
| Low TWA-BG | 2.81 | 1.42, 5.57 | 0.003 |
| Age | 1.01 | 0.99, 1.04 | 0.347 |
| Gender |  |  |  |
| Female | 1.00 | Reference |  |
| Male | 1.15 | 0.61, 2.18 | 0.661 |
| Weight | 1.00 | 0.99, 1.01 | 0.725 |
| SAPS II (time dependent) | 1.01 | 1.00, 1.02 | 0.004 |
| SOFA score (time dependent) | 1.04 | 1.01, 1.08 | 0.023 |
| Charlson comorbidity index | 1.10 | 0.92, 1.31 | 0.302 |
| CABG (time dependent) |  |  |  |
| YES | 1.00 | Reference |  |
| NO | 1.46 | 1.08, 1.97 | 0.014 |
| PCI |  |  |  |
| YES | 1.00 | Reference |  |
| NO | 0.47 | 0.14, 1.63 | 0.234 |
| IABP (time dependent) |  |  |  |
| YES | 1.00 | Reference |  |
| NO | 0.98 | 0.80, 1.20 | 0.858 |
| PiCCO |  |  |  |
| YES | 1.00 | Reference |  |
| NO | 6.34 | 0.48, 84.34 | 0.162 |
| NICOM (time dependent) |  |  |  |
| YES | 1.00 | Reference |  |
| NO | 0.80 | 0.52, 1.23 | 0.310 |
| Mechanical ventilation |  |  |  |
| YES | 1.00 | Reference |  |
| NO | 0.85 | 0.10, 7.58 | 0.883 |
| Sedative therapy |  |  |  |
| YES | 1.00 | Reference |  |
| NO | 3.85 | 0.40, 36.98 | 0.243 |
| HF |  |  |  |
| YES | 1.00 | Reference |  |
| NO | 1.63 | 0.74, 3.61 | 0.228 |
| Hypertension |  |  |  |
| YES | 1.00 | Reference |  |
| NO | 0.99 | 0.53, 1.85 | 0.971 |
| AFIB |  |  |  |
| YES | 1.00 | Reference |  |
| NO | 1.23 | 0.59, 2.56 | 0.588 |
| T2DM |  |  |  |
| YES | 1.00 | Reference |  |
| NO | 0.56 | 0.34, 0.93 | 0.024 |
| Renal |  |  |  |
| YES | 1.00 | Reference |  |
| NO | 2.77 | 1.43, 5.38 | 0.003 |
| Liver |  |  |  |
| YES | 1.00 | Reference |  |
| NO | 0.70 | 0.34, 1.45 | 0.335 |
| COPD (time dependent) |  |  |  |
| YES | 1.00 | Reference |  |
| NO | 0.82 | 0.66, 1.01 | 0.065 |
| CAD |  |  |  |
| YES | 1.00 | Reference |  |
| NO | 0.87 | 0.47, 1.61 | 0.663 |
| Stroke |  |  |  |
| YES | 1.00 | Reference |  |
| NO | 1.73 | 0.74, 4.06 | 0.205 |
| Malignancy |  |  |  |
| YES | 1.00 | Reference |  |
| NO | 1.76 | 0.67, 4.67 | 0.253 |
| Cardiomyopathy |  |  |  |
| YES | 1.00 | Reference |  |
| NO | 1.37 | 0.79, 2.39 | 0.259 |
| HVD |  |  |  |
| YES | 1.00 | Reference |  |
| NO | 1.31 | 0.77, 2.21 | 0.321 |
| MAP | 0.98 | 0.96, 0.99 | 0.002 |
| Temperature | 1.08 | 0.82, 1.43 | 0.592 |
| Heart rate (time dependent) | 1.00 | 1.00, 1.01 | 0.252 |
| WBC count (time dependent) | 1.01 | 1.00, 1.02 | 0.232 |
| Hemoglobin | 1.04 | 0.90, 1.20 | 0.600 |
| Platelet | 1.00 | 1.00, 1.00 | 0.499 |
| pH (time dependent) | 1.32 | 0.46, 3.83 | 0.609 |
| PO2 | 1.00 | 1.00, 1.00 | 0.441 |
| PCO2 | 1.00 | 0.97, 1.02 | 0.674 |
| Lactate (time dependent) | 1.00 | 0.97, 1.03 | 0.829 |
| Creatinine | 1.18 | 1.04, 1.35 | 0.010 |
| ^1^HR = Hazard Ratio, CI = Confidence Interval | | | |

## Table S19. Multivariate Cox model adjusted with all covariates for in-hospital mortality of original cohort 1

| **Characteristic** | **HR^1^** | **95% CI^1^** | **p-value** |
| --- | --- | --- | --- |
| Group |  |  |  |
| Optimal TWA-BG | 1.00 | Reference |  |
| Low TWA-BG | 1.89 | 1.29, 2.75 | <0.001 |
| Age | 1.01 | 1.00, 1.03 | 0.121 |
| Gender |  |  |  |
| Female | 1.00 | Reference |  |
| Male | 1.05 | 0.77, 1.42 | 0.762 |
| Weight | 1.00 | 0.99, 1.00 | 0.41 |
| SAPS II (time dependent) | 1.01 | 1.01, 1.02 | <0.001 |
| SOFA score (time dependent) | 1.00 | 0.99, 1.02 | 0.612 |
| Charlson comorbidity index | 1.09 | 0.99, 1.20 | 0.068 |
| CABG |  |  |  |
| YES | 1.00 | Reference |  |
| NO | 2.09 | 1.01, 4.36 | 0.048 |
| PCI |  |  |  |
| YES | 1.00 | Reference |  |
| NO | 0.94 | 0.42, 2.08 | 0.875 |
| IABP |  |  |  |
| YES | 1.00 | Reference |  |
| NO | 0.83 | 0.54, 1.26 | 0.38 |
| PiCCO |  |  |  |
| YES | 1.00 | Reference |  |
| NO | 1.08 | 0.23, 5.00 | 0.923 |
| NICOM |  |  |  |
| YES | 1.00 | Reference |  |
| NO | 0.54 | 0.27, 1.07 | 0.079 |
| Mechanical ventilation |  |  |  |
| YES | 1.00 | Reference |  |
| NO | 1.13 | 0.42, 3.02 | 0.806 |
| Sedative therapy |  |  |  |
| YES | 1.00 | Reference |  |
| NO | 1.66 | 0.63, 4.38 | 0.31 |
| HF (time dependent) |  |  |  |
| YES | 1.00 | Reference |  |
| NO | 1.07 | 0.96, 1.19 | 0.253 |
| Hypertension |  |  |  |
| YES | 1.00 | Reference |  |
| NO | 1.16 | 0.84, 1.59 | 0.37 |
| AFIB |  |  |  |
| YES | 1.00 | Reference |  |
| NO | 1.08 | 0.78, 1.51 | 0.642 |
| T2DM |  |  |  |
| YES | 1.00 | Reference |  |
| NO | 0.58 | 0.41, 0.83 | 0.003 |
| Renal |  |  |  |
| YES | 1.00 | Reference |  |
| NO | 1.53 | 1.03, 2.26 | 0.036 |
| Liver |  |  |  |
| YES | 1.00 | Reference |  |
| NO | 0.68 | 0.41, 1.13 | 0.141 |
| COPD |  |  |  |
| YES | 1.00 | Reference |  |
| NO | 0.68 | 0.48, 0.97 | 0.033 |
| CAD |  |  |  |
| YES | 1.00 | Reference |  |
| NO | 1.00 | 0.73, 1.37 | 0.979 |
| Stroke (time dependent) |  |  |  |
| YES | 1.00 | Reference |  |
| NO | 1.02 | 0.90, 1.16 | 0.729 |
| Malignancy |  |  |  |
| YES | 1.00 | Reference |  |
| NO | 1.19 | 0.75, 1.90 | 0.463 |
| Cardiomyopathy |  |  |  |
| YES | 1.00 | Reference |  |
| NO | 1.06 | 0.77, 1.47 | 0.709 |
| HVD (time dependent) |  |  |  |
| YES | 1.00 | Reference |  |
| NO | 1.07 | 0.98, 1.17 | 0.139 |
| MAP | 0.98 | 0.97, 0.99 | <0.001 |
| Temperature | 1.00 | 0.86, 1.17 | 0.999 |
| Heart rate | 1.01 | 1.00, 1.01 | 0.148 |
| WBC count | 1.01 | 1.00, 1.03 | 0.12 |
| Hemoglobin | 0.95 | 0.89, 1.02 | 0.137 |
| Platelet (time dependent) | 1.00 | 1.00, 1.00 | 0.864 |
| pH (time dependent) | 0.81 | 0.49, 1.32 | 0.39 |
| PO2 | 1.00 | 1.00, 1.00 | 0.188 |
| PCO2 | 1.00 | 0.99, 1.01 | 0.892 |
| Lactate (time dependent) | 1.00 | 0.99, 1.02 | 0.59 |
| Creatinine | 1.08 | 1.00, 1.16 | 0.046 |
| ^1^HR = Hazard Ratio, CI = Confidence Interval | | | |

## Table S20. Multivariate Cox model adjusted with unbalanced covariates for in-hospital mortality of original cohort 1

| **Characteristic** | **HR^1^** | **95% CI^1^** | **p-value** |
| --- | --- | --- | --- |
| Group |  |  |  |
| Optimal TWA-BG | 1.00 | Reference |  |
| Low TWA-BG | 1.95 | 1.35, 2.80 | <0.001 |
| Age | 1.03 | 1.01, 1.04 | <0.001 |
| Gender |  |  |  |
| Female | 1.00 | Reference |  |
| Male | 0.96 | 0.72, 1.27 | 0.775 |
| Charlson comorbidity index | 1.10 | 1.03, 1.17 | 0.007 |
| CABG |  |  |  |
| YES | 1.00 | Reference |  |
| NO | 1.92 | 0.95, 3.86 | 0.068 |
| IABP |  |  |  |
| YES | 1.00 | Reference |  |
| NO | 0.97 | 0.64, 1.46 | 0.88 |
| NICOM |  |  |  |
| YES | 1.00 | Reference |  |
| NO | 0.53 | 0.28, 1.00 | 0.051 |
| Mechanical ventilation |  |  |  |
| YES | 1.00 | Reference |  |
| NO | 0.76 | 0.33, 1.76 | 0.522 |
| Sedative therapy |  |  |  |
| YES | 1.00 | Reference |  |
| NO | 1.19 | 0.51, 2.76 | 0.692 |
| Hypertension (time dependent) |  |  |  |
| YES | 1.00 | Reference |  |
| NO | 1.06 | 0.97, 1.16 | 0.201 |
| Liver |  |  |  |
| YES | 1.00 | Reference |  |
| NO | 0.59 | 0.37, 0.97 | 0.037 |
| CAD |  |  |  |
| YES | 1.00 | Reference |  |
| NO | 1.07 | 0.79, 1.44 | 0.679 |
| Malignancy |  |  |  |
| YES | 1.00 | Reference |  |
| NO | 1.08 | 0.72, 1.62 | 0.712 |
| Cardiomyopathy |  |  |  |
| YES | 1.00 | Reference |  |
| NO | 1.10 | 0.81, 1.50 | 0.541 |
| HVD (time dependent) |  |  |  |
| YES | 1.00 | Reference |  |
| NO | 1.11 | 1.01, 1.20 | 0.022 |
| MAP | 0.98 | 0.98, 0.99 | <0.001 |
| Heart rate | 1.01 | 1.00, 1.02 | 0.007 |
| WBC count | 1.01 | 1.00, 1.03 | 0.162 |
| Platelet (time dependent) | 1.00 | 1.00, 1.00 | 0.583 |
| PO2 | 1.00 | 1.00, 1.00 | 0.136 |
| Lactate | 1.09 | 1.05, 1.14 | <0.001 |
| Creatinine | 1.10 | 1.03, 1.18 | 0.003 |
| ^1^HR = Hazard Ratio, CI = Confidence Interval | | | |

## Table S21. Multivariate Cox model adjusted with all covariates and IPTW for in-hospital mortality of cohort 1

| **Characteristic** | **HR^1^** | **95% CI^1^** | **p-value** |
| --- | --- | --- | --- |
| Group |  |  |  |
| Optimal TWA-BG | 1.00 | Reference |  |
| Low TWA-BG | 2.07 | 1.23, 3.48 | 0.006 |
| Age | 1.01 | 0.99, 1.03 | 0.562 |
| Gender |  |  |  |
| Female | 1.00 | Reference |  |
| Male | 1.29 | 0.77, 2.15 | 0.334 |
| Weight | 1.00 | 0.99, 1.01 | 0.609 |
| SAPS II (time dependent) | 1.01 | 1.01, 1.02 | <0.001 |
| SOFA score (time dependent) | 1.02 | 1.00, 1.05 | 0.106 |
| Charlson comorbidity index (time dependent) | 1.02 | 0.98, 1.06 | 0.251 |
| CABG (time dependent) |  |  |  |
| YES | 1.00 | Reference |  |
| NO | 1.40 | 1.07, 1.83 | 0.013 |
| PCI |  |  |  |
| YES | 1.00 | Reference |  |
| NO | 0.53 | 0.16, 1.81 | 0.314 |
| IABP |  |  |  |
| YES | 1.00 | Reference |  |
| NO | 1.04 | 0.60, 1.82 | 0.881 |
| PiCCO (time dependent) |  |  |  |
| YES | 1.00 | Reference |  |
| NO | 1.34 | 0.66, 2.70 | 0.421 |
| NICOM (time dependent) |  |  |  |
| YES | 1.00 | Reference |  |
| NO | 0.82 | 0.56, 1.20 | 0.304 |
| Mechanical ventilation (time dependent) |  |  |  |
| YES | 1.00 | Reference |  |
| NO | 1.30 | 0.82, 2.08 | 0.269 |
| Sedative therapy (time dependent) |  |  |  |
| YES | 1.00 | Reference |  |
| NO | 1.03 | 0.65, 1.63 | 0.912 |
| HF (time dependent) |  |  |  |
| YES | 1.00 | Reference |  |
| NO | 1.21 | 0.99, 1.48 | 0.069 |
| Hypertension |  |  |  |
| YES | 1.00 | Reference |  |
| NO | 1.10 | 0.67, 1.81 | 0.712 |
| AFIB |  |  |  |
| YES | 1.00 | Reference |  |
| NO | 0.96 | 0.53, 1.72 | 0.88 |
| T2DM (time dependent) |  |  |  |
| YES | 1.00 | Reference |  |
| NO | 0.82 | 0.71, 0.95 | 0.007 |
| Renal (time dependent) |  |  |  |
| YES | 1.00 | Reference |  |
| NO | 1.14 | 0.96, 1.35 | 0.126 |
| Liver |  |  |  |
| YES | 1.00 | Reference |  |
| NO | 0.70 | 0.35, 1.39 | 0.303 |
| COPD |  |  |  |
| YES | 1.00 | Reference |  |
| NO | 0.53 | 0.29, 0.97 | 0.041 |
| CAD |  |  |  |
| YES | 1.00 | Reference |  |
| NO | 1.18 | 0.70, 1.97 | 0.542 |
| Stroke (time dependent) |  |  |  |
| YES | 1.00 | Reference |  |
| NO | 1.05 | 0.84, 1.30 | 0.69 |
| Malignancy |  |  |  |
| YES | 1.00 | Reference |  |
| NO | 1.73 | 0.87, 3.43 | 0.119 |
| Cardiomyopathy (time dependent) |  |  |  |
| YES | 1.00 | Reference |  |
| NO | 1.02 | 0.89, 1.15 | 0.802 |
| HVD (time dependent) |  |  |  |
| YES | 1.00 | Reference |  |
| NO | 1.10 | 0.98, 1.24 | 0.097 |
| MAP (time dependent) | 0.99 | 0.99, 1.00 | <0.001 |
| Temperature | 1.06 | 0.83, 1.34 | 0.638 |
| Heart rate (time dependent) | 1.00 | 1.00, 1.00 | 0.259 |
| WBC count (time dependent) | 1.00 | 1.00, 1.01 | 0.388 |
| Hemoglobin (time dependent) | 0.99 | 0.95, 1.02 | 0.46 |
| Platelet (time dependent) | 1.00 | 1.00, 1.00 | 0.879 |
| pH | 2.22 | 0.16, 30.01 | 0.548 |
| PO2 | 1.00 | 1.00, 1.00 | 0.046 |
| PCO2 (time dependent) | 1.00 | 0.99, 1.00 | 0.837 |
| Lactate | 1.02 | 0.95, 1.11 | 0.569 |
| Creatinine (time dependent) | 1.00 | 0.97, 1.03 | 0.8 |
| ^1^HR = Hazard Ratio, CI = Confidence Interval | | | |

## Table S22. Survey-weighted Cox model adjusted with all covariates and IPTW for in-hospital mortality of cohort 1

| **Characteristic** | **HR^1^** | **95% CI^1^** | **p-value** |
| --- | --- | --- | --- |
| Group |  |  |  |
| Optimal TWA-BG | 1.00 | Reference |  |
| Low TWA-BG | 2.09 | 1.24, 3.52 | 0.006 |
| Age | 1.01 | 0.99, 1.03 | 0.519 |
| Gender |  |  |  |
| Female | 1.00 | Reference |  |
| Male | 1.29 | 0.77, 2.16 | 0.328 |
| Weight | 1.00 | 0.99, 1.01 | 0.639 |
| SAPS II (time dependent) | 1.01 | 1.01, 1.02 | <0.001 |
| SOFA score (time dependent) | 1.02 | 1.00, 1.04 | 0.11 |
| Charlson comorbidity index | 1.07 | 0.94, 1.22 | 0.32 |
| CABG |  |  |  |
| YES | 1.00 | Reference |  |
| NO | 3.25 | 1.35, 7.83 | 0.008 |
| PCI |  |  |  |
| YES | 1.00 | Reference |  |
| NO | 0.54 | 0.16, 1.87 | 0.333 |
| IABP |  |  |  |
| YES | 1.00 | Reference |  |
| NO | 1.04 | 0.59, 1.83 | 0.887 |
| PiCCO |  |  |  |
| YES | 1.00 | Reference |  |
| NO | 2.66 | 0.30, 23.50 | 0.378 |
| NICOM |  |  |  |
| YES | 1.00 | Reference |  |
| NO | 0.49 | 0.13, 1.82 | 0.286 |
| Mechanical ventilation (time dependent) |  |  |  |
| YES | 1.00 | Reference |  |
| NO | 1.37 | 0.89, 2.09 | 0.148 |
| Sedative therapy |  |  |  |
| YES | 1.00 | Reference |  |
| NO | 0.91 | 0.23, 3.63 | 0.898 |
| HF (time dependent) |  |  |  |
| YES | 1.00 | Reference |  |
| NO | 1.20 | 0.98, 1.47 | 0.08 |
| Hypertension |  |  |  |
| YES | 1.00 | Reference |  |
| NO | 1.08 | 0.66, 1.78 | 0.75 |
| AFIB |  |  |  |
| YES | 1.00 | Reference |  |
| NO | 0.96 | 0.53, 1.73 | 0.888 |
| T2DM |  |  |  |
| YES | 1.00 | Reference |  |
| NO | 0.54 | 0.34, 0.87 | 0.011 |
| Renal |  |  |  |
| YES | 1.00 | Reference |  |
| NO | 1.59 | 0.90, 2.81 | 0.107 |
| Liver |  |  |  |
| YES | 1.00 | Reference |  |
| NO | 0.70 | 0.35, 1.38 | 0.303 |
| COPD |  |  |  |
| YES | 1.00 | Reference |  |
| NO | 0.52 | 0.29, 0.94 | 0.029 |
| CAD |  |  |  |
| YES | 1.00 | Reference |  |
| NO | 1.16 | 0.69, 1.95 | 0.574 |
| Stroke (time dependent) |  |  |  |
| YES | 1.00 | Reference |  |
| NO | 1.05 | 0.84, 1.31 | 0.673 |
| Malignancy |  |  |  |
| YES | 1.00 | Reference |  |
| NO | 1.71 | 0.86, 3.40 | 0.125 |
| Cardiomyopathy |  |  |  |
| YES | 1.00 | Reference |  |
| NO | 1.10 | 0.72, 1.70 | 0.65 |
| HVD (time dependent) |  |  |  |
| YES | 1.00 | Reference |  |
| NO | 1.10 | 0.98, 1.23 | 0.111 |
| MAP | 0.98 | 0.97, 0.99 | <0.001 |
| Temperature | 1.05 | 0.83, 1.34 | 0.661 |
| Heart rate | 1.01 | 1.00, 1.02 | 0.218 |
| WBC count (time dependent) | 1.00 | 1.00, 1.01 | 0.383 |
| Hemoglobin | 0.96 | 0.85, 1.09 | 0.52 |
| Platelet (time dependent) | 1.00 | 1.00, 1.00 | 0.911 |
| pH (time dependent) | 1.28 | 0.60, 2.72 | 0.524 |
| PO2 | 1.00 | 1.00, 1.00 | 0.049 |
| PCO2 | 1.00 | 0.98, 1.01 | 0.738 |
| Lactate (time dependent) | 1.01 | 0.98, 1.03 | 0.644 |
| Creatinine | 1.03 | 0.93, 1.13 | 0.622 |
| ^1^HR = Hazard Ratio, CI = Confidence Interval | | | |

## Table S23. Multivariate Cox model adjusted with all covariates for 28-day mortality of original cohort 2

| **Characteristic** | **HR^1^** | **95% CI^1^** | **p-value** |
| --- | --- | --- | --- |
| Group (time dependent) |  |  |  |
| Optimal TWA-BG | 1.00 | Reference |  |
| High TWA-BG | 1.15 | 1.08, 1.22 | <0.001 |
| Age (time dependent) | 1.01 | 1.00, 1.01 | <0.001 |
| Gender |  |  |  |
| Female | 1.00 | Reference |  |
| Male | 0.86 | 0.71, 1.04 | 0.127 |
| Weight | 1.00 | 0.99, 1.00 | 0.736 |
| SAPS II (time dependent) | 1.01 | 1.00, 1.01 | <0.001 |
| SOFA score (time dependent) | 1.01 | 1.00, 1.03 | 0.025 |
| Charlson comorbidity index (time dependent) | 1.02 | 1.00, 1.04 | 0.067 |
| CABG |  |  |  |
| YES | 1.00 | Reference |  |
| NO | 2.59 | 1.51, 4.46 | <0.001 |
| PCI (time dependent) |  |  |  |
| YES | 1.00 | Reference |  |
| NO | 0.99 | 0.87, 1.13 | 0.93 |
| IABP (time dependent) |  |  |  |
| YES | 1.00 | Reference |  |
| NO | 0.99 | 0.92, 1.07 | 0.818 |
| PiCCO |  |  |  |
| YES | 1.00 | Reference |  |
| NO | 0.82 | 0.26, 2.64 | 0.741 |
| NICOM |  |  |  |
| YES | 1.00 | Reference |  |
| NO | 1.23 | 0.79, 1.93 | 0.359 |
| Mechanical ventilation (time dependent) |  |  |  |
| YES | 1.00 | Reference |  |
| NO | 0.94 | 0.81, 1.10 | 0.449 |
| Sedative therapy (time dependent) |  |  |  |
| YES | 1.00 | Reference |  |
| NO | 1.20 | 1.03, 1.39 | 0.018 |
| HF (time dependent) |  |  |  |
| YES | 1.00 | Reference |  |
| NO | 1.12 | 1.05, 1.20 | 0.001 |
| Hypertension |  |  |  |
| YES | 1.00 | Reference |  |
| NO | 1.04 | 0.83, 1.29 | 0.741 |
| AFIB |  |  |  |
| YES | 1.00 | Reference |  |
| NO | 1.06 | 0.87, 1.30 | 0.572 |
| T2DM |  |  |  |
| YES | 1.00 | Reference |  |
| NO | 1.37 | 1.11, 1.69 | 0.003 |
| Renal |  |  |  |
| YES | 1.00 | Reference |  |
| NO | 1.12 | 0.86, 1.44 | 0.404 |
| Liver |  |  |  |
| YES | 1.00 | Reference |  |
| NO | 1.09 | 0.71, 1.68 | 0.688 |
| COPD |  |  |  |
| YES | 1.00 | Reference |  |
| NO | 0.84 | 0.68, 1.05 | 0.128 |
| CAD |  |  |  |
| YES | 1.00 | Reference |  |
| NO | 0.89 | 0.73, 1.08 | 0.227 |
| Stroke |  |  |  |
| YES | 1.00 | Reference |  |
| NO | 1.12 | 0.83, 1.51 | 0.461 |
| Malignancy |  |  |  |
| YES | 1.00 | Reference |  |
| NO | 1.14 | 0.85, 1.52 | 0.385 |
| Cardiomyopathy (time dependent) |  |  |  |
| YES | 1.00 | Reference |  |
| NO | 0.97 | 0.92, 1.04 | 0.403 |
| HVD (time dependent) |  |  |  |
| YES | 1.00 | Reference |  |
| NO | 1.05 | 0.99, 1.11 | 0.092 |
| MAP | 1.00 | 0.99, 1.00 | 0.644 |
| Temperature | 0.94 | 0.85, 1.03 | 0.184 |
| Heart rate | 1.01 | 1.00, 1.01 | 0.004 |
| WBC count | 1.00 | 0.99, 1.01 | 0.518 |
| Hemoglobin | 0.97 | 0.93, 1.01 | 0.189 |
| Platelet | 1.00 | 1.00, 1.00 | 0.411 |
| pH (time dependent) | 0.93 | 0.69, 1.26 | 0.649 |
| PO2 | 1.00 | 1.00, 1.00 | 0.837 |
| PCO2 | 1.01 | 1.00, 1.01 | 0.148 |
| Lactate (time dependent) | 1.02 | 1.01, 1.03 | <0.001 |
| Creatinine | 1.07 | 1.01, 1.14 | 0.018 |
| ^1^HR = Hazard Ratio, CI = Confidence Interval | | | |

## Table S24. Multivariate Cox model adjusted with unbalanced covariates for 28-day mortality of original cohort 2

| **Characteristic** | **HR^1^** | **95% CI^1^** | **p-value** |
| --- | --- | --- | --- |
| Group (time dependent) |  |  |  |
| Optimal TWA-BG | 1.00 | Reference |  |
| High TWA-BG | 1.15 | 1.08, 1.22 | <0.001 |
| Age (time dependent) | 1.00 | 1.00, 1.01 | <0.001 |
| Weight | 1.00 | 0.99, 1.00 | 0.204 |
| SAPS II (time dependent) | 1.01 | 1.01, 1.01 | <0.001 |
| Charlson comorbidity index (time dependent) | 1.02 | 1.00, 1.03 | 0.014 |
| CABG |  |  |  |
| YES | 1.00 | Reference |  |
| NO | 2.61 | 1.54, 4.43 | <0.001 |
| NICOM |  |  |  |
| YES | 1.00 | Reference |  |
| NO | 1.22 | 0.79, 1.89 | 0.377 |
| HF (time dependent) |  |  |  |
| YES | 1.00 | Reference |  |
| NO | 1.11 | 1.04, 1.18 | 0.002 |
| Hypertension |  |  |  |
| YES | 1.00 | Reference |  |
| NO | 1.06 | 0.85, 1.31 | 0.617 |
| T2DM |  |  |  |
| YES | 1.00 | Reference |  |
| NO | 1.30 | 1.06, 1.59 | 0.012 |
| Renal (time dependent) |  |  |  |
| YES | 1.00 | Reference |  |
| NO | 1.03 | 0.96, 1.10 | 0.483 |
| CAD |  |  |  |
| YES | 1.00 | Reference |  |
| NO | 0.96 | 0.80, 1.15 | 0.65 |
| HVD (time dependent) |  |  |  |
| YES | 1.00 | Reference |  |
| NO | 1.03 | 0.98, 1.09 | 0.249 |
| WBC count | 1.00 | 0.99, 1.01 | 0.434 |
| Platelet | 1.00 | 1.00, 1.00 | 0.626 |
| pH (time dependent) | 0.73 | 0.58, 0.93 | 0.009 |
| PO2 | 1.00 | 1.00, 1.00 | 0.508 |
| Creatinine | 1.09 | 1.03, 1.15 | 0.001 |
| ^1^HR = Hazard Ratio, CI = Confidence Interval | | | |

## Table S25. Multivariate Cox model adjusted with all covariates and IPTW for 28-day mortality of cohort 2

| **Characteristic** | **HR^1^** | **95% CI^1^** | **p-value** |
| --- | --- | --- | --- |
| Group |  |  |  |
| Optimal TWA-BG | 1.00 | Reference |  |
| High TWA-BG | 1.38 | 1.08, 1.76 | 0.009 |
| Age (time dependent) | 1.00 | 1.00, 1.01 | 0.063 |
| Gender (time dependent) |  |  |  |
| Female | 1.00 | Reference |  |
| Male | 0.95 | 0.89, 1.01 | 0.121 |
| Weight | 1.00 | 0.99, 1.00 | 0.474 |
| SAPS II (time dependent) | 1.01 | 1.01, 1.01 | <0.001 |
| SOFA score (time dependent) | 1.01 | 1.00, 1.03 | 0.137 |
| Charlson comorbidity index (time dependent) | 1.04 | 1.01, 1.07 | 0.013 |
| CABG |  |  |  |
| YES | 1.00 | Reference |  |
| NO | 3.07 | 1.65, 5.72 | <0.001 |
| PCI (time dependent) |  |  |  |
| YES | 1.00 | Reference |  |
| NO | 0.95 | 0.81, 1.12 | 0.567 |
| IABP (time dependent) |  |  |  |
| YES | 1.00 | Reference |  |
| NO | 1.03 | 0.94, 1.13 | 0.474 |
| PiCCO |  |  |  |
| YES | 1.00 | Reference |  |
| NO | 1.09 | 0.28, 4.18 | 0.902 |
| NICOM (time dependent) |  |  |  |
| YES | 1.00 | Reference |  |
| NO | 1.02 | 0.83, 1.24 | 0.875 |
| Mechanical ventilation (time dependent) |  |  |  |
| YES | 1.00 | Reference |  |
| NO | 1.01 | 0.80, 1.28 | 0.927 |
| Sedative therapy (time dependent) |  |  |  |
| YES | 1.00 | Reference |  |
| NO | 1.15 | 0.91, 1.46 | 0.242 |
| HF (time dependent) |  |  |  |
| YES | 1.00 | Reference |  |
| NO | 1.13 | 1.04, 1.22 | 0.002 |
| Hypertension |  |  |  |
| YES | 1.00 | Reference |  |
| NO | 1.00 | 0.76, 1.31 | 0.984 |
| AFIB (time dependent) |  |  |  |
| YES | 1.00 | Reference |  |
| NO | 1.03 | 0.96, 1.11 | 0.442 |
| T2DM (time dependent) |  |  |  |
| YES | 1.00 | Reference |  |
| NO | 1.09 | 1.02, 1.17 | 0.011 |
| Renal (time dependent) |  |  |  |
| YES | 1.00 | Reference |  |
| NO | 1.07 | 0.97, 1.17 | 0.19 |
| Liver (time dependent) |  |  |  |
| YES | 1.00 | Reference |  |
| NO | 1.11 | 0.96, 1.29 | 0.176 |
| COPD |  |  |  |
| YES | 1.00 | Reference |  |
| NO | 0.89 | 0.67, 1.17 | 0.393 |
| CAD |  |  |  |
| YES | 1.00 | Reference |  |
| NO | 0.94 | 0.74, 1.20 | 0.632 |
| Stroke |  |  |  |
| YES | 1.00 | Reference |  |
| NO | 1.18 | 0.80, 1.75 | 0.394 |
| Malignancy |  |  |  |
| YES | 1.00 | Reference |  |
| NO | 1.11 | 0.82, 1.50 | 0.502 |
| Cardiomyopathy (time dependent) |  |  |  |
| YES | 1.00 | Reference |  |
| NO | 0.98 | 0.91, 1.06 | 0.658 |
| HVD (time dependent) |  |  |  |
| YES | 1.00 | Reference |  |
| NO | 1.05 | 0.98, 1.12 | 0.163 |
| MAP (time dependent) | 1.00 | 1.00, 1.00 | 0.278 |
| Temperature | 0.95 | 0.85, 1.06 | 0.35 |
| Heart rate | 1.00 | 1.00, 1.01 | 0.037 |
| WBC count | 1.00 | 0.99, 1.01 | 0.671 |
| Hemoglobin | 0.98 | 0.93, 1.03 | 0.363 |
| Platelet | 1.00 | 1.00, 1.00 | 0.484 |
| pH (time dependent) | 1.16 | 0.82, 1.63 | 0.403 |
| PO2 | 1.00 | 1.00, 1.00 | 0.817 |
| PCO2 (time dependent) | 1.00 | 1.00, 1.01 | 0.014 |
| Lactate (time dependent) | 1.03 | 1.01, 1.04 | <0.001 |
| Creatinine | 1.11 | 1.03, 1.18 | 0.003 |
| ^1^HR = Hazard Ratio, CI = Confidence Interval | | | |

## Table S26. Survey-weighted Cox model adjusted with all covariates and IPTW for 28-day mortality of cohort 2

| **Characteristic** | **HR^1^** | **95% CI^1^** | **p-value** |
| --- | --- | --- | --- |
| Group (time dependent) |  |  |  |
| Optimal TWA-BG | 1.00 | Reference |  |
| High TWA-BG | 1.10 | 1.02, 1.18 | 0.011 |
| Age (time dependent) | 1.00 | 1.00, 1.01 | 0.061 |
| Gender |  |  |  |
| Female | 1.00 | Reference |  |
| Male | 0.83 | 0.66, 1.04 | 0.105 |
| Weight | 1.00 | 0.99, 1.00 | 0.485 |
| SAPS II (time dependent) | 1.01 | 1.01, 1.01 | <0.001 |
| SOFA score (time dependent) | 1.01 | 1.00, 1.03 | 0.137 |
| Charlson comorbidity index (time dependent) | 1.04 | 1.01, 1.07 | 0.011 |
| CABG |  |  |  |
| YES | 1.00 | Reference |  |
| NO | 3.08 | 1.65, 5.73 | <0.001 |
| PCI (time dependent) |  |  |  |
| YES | 1.00 | Reference |  |
| NO | 0.95 | 0.81, 1.12 | 0.567 |
| IABP (time dependent) |  |  |  |
| YES | 1.00 | Reference |  |
| NO | 1.03 | 0.94, 1.13 | 0.478 |
| PiCCO |  |  |  |
| YES | 1.00 | Reference |  |
| NO | 1.09 | 0.28, 4.19 | 0.901 |
| NICOM |  |  |  |
| YES | 1.00 | Reference |  |
| NO | 1.01 | 0.51, 2.01 | 0.971 |
| Mechanical ventilation (time dependent) |  |  |  |
| YES | 1.00 | Reference |  |
| NO | 1.01 | 0.80, 1.28 | 0.929 |
| Sedative therapy (time dependent) |  |  |  |
| YES | 1.00 | Reference |  |
| NO | 1.15 | 0.91, 1.46 | 0.245 |
| HF (time dependent) |  |  |  |
| YES | 1.00 | Reference |  |
| NO | 1.13 | 1.04, 1.21 | 0.002 |
| Hypertension |  |  |  |
| YES | 1.00 | Reference |  |
| NO | 1.00 | 0.76, 1.31 | 0.976 |
| AFIB |  |  |  |
| YES | 1.00 | Reference |  |
| NO | 1.11 | 0.87, 1.42 | 0.401 |
| T2DM |  |  |  |
| YES | 1.00 | Reference |  |
| NO | 1.32 | 1.05, 1.66 | 0.015 |
| Renal |  |  |  |
| YES | 1.00 | Reference |  |
| NO | 1.26 | 0.92, 1.73 | 0.148 |
| Liver |  |  |  |
| YES | 1.00 | Reference |  |
| NO | 1.43 | 0.88, 2.34 | 0.149 |
| COPD |  |  |  |
| YES | 1.00 | Reference |  |
| NO | 0.89 | 0.67, 1.16 | 0.386 |
| CAD |  |  |  |
| YES | 1.00 | Reference |  |
| NO | 0.94 | 0.74, 1.20 | 0.618 |
| Stroke |  |  |  |
| YES | 1.00 | Reference |  |
| NO | 1.18 | 0.80, 1.75 | 0.393 |
| Malignancy |  |  |  |
| YES | 1.00 | Reference |  |
| NO | 1.11 | 0.82, 1.50 | 0.505 |
| Cardiomyopathy (time dependent) |  |  |  |
| YES | 1.00 | Reference |  |
| NO | 0.98 | 0.91, 1.06 | 0.645 |
| HVD (time dependent) |  |  |  |
| YES | 1.00 | Reference |  |
| NO | 1.05 | 0.98, 1.12 | 0.168 |
| MAP | 1.00 | 0.99, 1.00 | 0.245 |
| Temperature | 0.95 | 0.85, 1.06 | 0.336 |
| Heart rate | 1.00 | 1.00, 1.01 | 0.036 |
| WBC count | 1.00 | 0.99, 1.01 | 0.659 |
| Hemoglobin | 0.98 | 0.93, 1.03 | 0.384 |
| Platelet | 1.00 | 1.00, 1.00 | 0.498 |
| pH (time dependent) | 1.17 | 0.83, 1.65 | 0.375 |
| PO2 | 1.00 | 1.00, 1.00 | 0.817 |
| PCO2 | 1.01 | 1.00, 1.02 | 0.01 |
| Lactate (time dependent) | 1.03 | 1.01, 1.04 | <0.001 |
| Creatinine | 1.11 | 1.04, 1.19 | 0.002 |
| ^1^HR = Hazard Ratio, CI = Confidence Interval | | | |

## Table S27. Multivariate Cox model adjusted with all covariates for ICU mortality of original cohort 2

| **Characteristic** | **HR^1^** | **95% CI^1^** | **p-value** |
| --- | --- | --- | --- |
| Group |  |  |  |
| Optimal TWA-BG | 1.00 | Reference |  |
| High TWA-BG | 1.87 | 1.45, 2.41 | <0.001 |
| Age | 1.02 | 1.01, 1.03 | 0.006 |
| Gender |  |  |  |
| Female | 1.00 | Reference |  |
| Male | 0.93 | 0.74, 1.18 | 0.554 |
| Weight | 1.00 | 0.99, 1.00 | 0.346 |
| SAPS II (time dependent) | 1.01 | 1.00, 1.01 | <0.001 |
| SOFA score (time dependent) | 1.01 | 1.00, 1.03 | 0.115 |
| Charlson comorbidity index | 1.04 | 0.96, 1.13 | 0.344 |
| CABG |  |  |  |
| YES | 1.00 | Reference |  |
| NO | 2.30 | 1.30, 4.04 | 0.004 |
| PCI |  |  |  |
| YES | 1.00 | Reference |  |
| NO | 0.84 | 0.51, 1.39 | 0.497 |
| IABP |  |  |  |
| YES | 1.00 | Reference |  |
| NO | 0.97 | 0.72, 1.30 | 0.839 |
| PiCCO |  |  |  |
| YES | 1.00 | Reference |  |
| NO | 1.08 | 0.26, 4.50 | 0.912 |
| NICOM (time dependent) |  |  |  |
| YES | 1.00 | Reference |  |
| NO | 1.12 | 0.95, 1.31 | 0.166 |
| Mechanical ventilation (time dependent) |  |  |  |
| YES | 1.00 | Reference |  |
| NO | 0.91 | 0.74, 1.12 | 0.387 |
| Sedative therapy |  |  |  |
| YES | 1.00 | Reference |  |
| NO | 1.90 | 0.98, 3.67 | 0.057 |
| HF (time dependent) |  |  |  |
| YES | 1.00 | Reference |  |
| NO | 1.14 | 1.04, 1.24 | 0.003 |
| Hypertension |  |  |  |
| YES | 1.00 | Reference |  |
| NO | 1.04 | 0.80, 1.36 | 0.757 |
| AFIB |  |  |  |
| YES | 1.00 | Reference |  |
| NO | 1.24 | 0.96, 1.59 | 0.102 |
| T2DM |  |  |  |
| YES | 1.00 | Reference |  |
| NO | 1.31 | 1.01, 1.69 | 0.039 |
| Renal |  |  |  |
| YES | 1.00 | Reference |  |
| NO | 1.26 | 0.91, 1.74 | 0.16 |
| Liver |  |  |  |
| YES | 1.00 | Reference |  |
| NO | 1.21 | 0.70, 2.07 | 0.499 |
| COPD |  |  |  |
| YES | 1.00 | Reference |  |
| NO | 0.89 | 0.68, 1.17 | 0.403 |
| CAD |  |  |  |
| YES | 1.00 | Reference |  |
| NO | 0.87 | 0.68, 1.11 | 0.261 |
| Stroke |  |  |  |
| YES | 1.00 | Reference |  |
| NO | 1.17 | 0.83, 1.65 | 0.375 |
| Malignancy |  |  |  |
| YES | 1.00 | Reference |  |
| NO | 1.36 | 0.93, 1.98 | 0.113 |
| Cardiomyopathy |  |  |  |
| YES | 1.00 | Reference |  |
| NO | 1.18 | 0.90, 1.54 | 0.235 |
| HVD (time dependent) |  |  |  |
| YES | 1.00 | Reference |  |
| NO | 1.07 | 1.00, 1.15 | 0.055 |
| MAP | 1.00 | 0.99, 1.00 | 0.349 |
| Temperature | 0.97 | 0.87, 1.08 | 0.536 |
| Heart rate | 1.01 | 1.00, 1.01 | 0.025 |
| WBC count | 1.00 | 0.99, 1.01 | 0.817 |
| Hemoglobin | 0.98 | 0.94, 1.03 | 0.531 |
| Platelet | 1.00 | 1.00, 1.00 | 0.72 |
| pH (time dependent) | 0.76 | 0.51, 1.11 | 0.157 |
| PO2 | 1.00 | 1.00, 1.00 | 0.413 |
| PCO2 | 1.00 | 0.99, 1.01 | 0.458 |
| Lactate (time dependent) | 1.02 | 1.01, 1.03 | 0.003 |
| Creatinine | 1.11 | 1.03, 1.20 | 0.007 |
| ^1^HR = Hazard Ratio, CI = Confidence Interval | | | |

## Table S28. Multivariate Cox model adjusted with unbalanced covariates for ICU mortality of original cohort 2

| **Characteristic** | **HR^1^** | **95% CI^1^** | **p-value** |
| --- | --- | --- | --- |
| Group |  |  |  |
| Optimal TWA-BG | 1.00 | Reference |  |
| High TWA-BG | 1.82 | 1.42, 2.34 | <0.001 |
| Age | 1.02 | 1.00, 1.03 | 0.005 |
| Weight | 1.00 | 0.99, 1.00 | 0.139 |
| SAPS II (time dependent) | 1.01 | 1.01, 1.01 | <0.001 |
| Charlson comorbidity index | 1.02 | 0.95, 1.08 | 0.604 |
| CABG |  |  |  |
| YES | 1.00 | Reference |  |
| NO | 2.14 | 1.24, 3.69 | 0.006 |
| NICOM (time dependent) |  |  |  |
| YES | 1.00 | Reference |  |
| NO | 1.10 | 0.94, 1.28 | 0.229 |
| HF (time dependent) |  |  |  |
| YES | 1.00 | Reference |  |
| NO | 1.12 | 1.04, 1.21 | 0.004 |
| Hypertension |  |  |  |
| YES | 1.00 | Reference |  |
| NO | 1.04 | 0.80, 1.34 | 0.783 |
| T2DM |  |  |  |
| YES | 1.00 | Reference |  |
| NO | 1.19 | 0.93, 1.52 | 0.167 |
| Renal |  |  |  |
| YES | 1.00 | Reference |  |
| NO | 1.18 | 0.88, 1.59 | 0.268 |
| CAD |  |  |  |
| YES | 1.00 | Reference |  |
| NO | 0.87 | 0.70, 1.09 | 0.236 |
| HVD (time dependent) |  |  |  |
| YES | 1.00 | Reference |  |
| NO | 1.05 | 0.98, 1.13 | 0.138 |
| WBC count | 1.00 | 0.99, 1.01 | 0.914 |
| Platelet | 1.00 | 1.00, 1.00 | 0.586 |
| pH (time dependent) | 0.60 | 0.45, 0.80 | <0.001 |
| PO2 | 1.00 | 1.00, 1.00 | 0.882 |
| Creatinine | 1.12 | 1.05, 1.20 | 0.001 |
| ^1^HR = Hazard Ratio, CI = Confidence Interval | | | |

## Table S29. Multivariate Cox model adjusted with all covariates and IPTW for ICU mortality of cohort 2

| **Characteristic** | **HR^1^** | **95% CI^1^** | **p-value** |
| --- | --- | --- | --- |
| Group |  |  |  |
| Optimal TWA-BG | 1.00 | Reference |  |
| High TWA-BG | 1.38 | 1.01, 1.87 | 0.043 |
| Age | 1.01 | 0.99, 1.02 | 0.289 |
| Gender |  |  |  |
| Female | 1.00 | Reference |  |
| Male | 0.92 | 0.70, 1.20 | 0.525 |
| Weight | 0.99 | 0.99, 1.00 | 0.145 |
| SAPS II (time dependent) | 1.01 | 1.00, 1.01 | <0.001 |
| SOFA score (time dependent) | 1.01 | 0.99, 1.03 | 0.333 |
| Charlson comorbidity index | 1.14 | 1.02, 1.28 | 0.019 |
| CABG |  |  |  |
| YES | 1.00 | Reference |  |
| NO | 3.05 | 1.59, 5.84 | <0.001 |
| PCI |  |  |  |
| YES | 1.00 | Reference |  |
| NO | 0.63 | 0.35, 1.14 | 0.13 |
| IABP |  |  |  |
| YES | 1.00 | Reference |  |
| NO | 1.10 | 0.78, 1.54 | 0.6 |
| PiCCO |  |  |  |
| YES | 1.00 | Reference |  |
| NO | 1.72 | 0.35, 8.40 | 0.505 |
| NICOM (time dependent) |  |  |  |
| YES | 1.00 | Reference |  |
| NO | 1.03 | 0.81, 1.31 | 0.82 |
| Mechanical ventilation |  |  |  |
| YES | 1.00 | Reference |  |
| NO | 0.63 | 0.22, 1.79 | 0.39 |
| Sedative therapy |  |  |  |
| YES | 1.00 | Reference |  |
| NO | 2.46 | 0.86, 7.02 | 0.094 |
| HF (time dependent) |  |  |  |
| YES | 1.00 | Reference |  |
| NO | 1.15 | 1.04, 1.26 | 0.005 |
| Hypertension |  |  |  |
| YES | 1.00 | Reference |  |
| NO | 0.98 | 0.70, 1.38 | 0.926 |
| AFIB |  |  |  |
| YES | 1.00 | Reference |  |
| NO | 1.48 | 1.09, 2.00 | 0.011 |
| T2DM |  |  |  |
| YES | 1.00 | Reference |  |
| NO | 1.18 | 0.90, 1.55 | 0.225 |
| Renal |  |  |  |
| YES | 1.00 | Reference |  |
| NO | 1.47 | 1.00, 2.19 | 0.053 |
| Liver |  |  |  |
| YES | 1.00 | Reference |  |
| NO | 1.68 | 0.89, 3.18 | 0.111 |
| COPD |  |  |  |
| YES | 1.00 | Reference |  |
| NO | 0.96 | 0.69, 1.34 | 0.816 |
| CAD |  |  |  |
| YES | 1.00 | Reference |  |
| NO | 0.94 | 0.70, 1.27 | 0.696 |
| Stroke (time dependent) |  |  |  |
| YES | 1.00 | Reference |  |
| NO | 1.07 | 0.94, 1.21 | 0.312 |
| Malignancy |  |  |  |
| YES | 1.00 | Reference |  |
| NO | 1.42 | 0.94, 2.13 | 0.093 |
| Cardiomyopathy |  |  |  |
| YES | 1.00 | Reference |  |
| NO | 1.23 | 0.89, 1.71 | 0.211 |
| HVD (time dependent) |  |  |  |
| YES | 1.00 | Reference |  |
| NO | 1.08 | 0.99, 1.17 | 0.071 |
| MAP | 0.99 | 0.99, 1.00 | 0.074 |
| Temperature (time dependent) | 1.00 | 0.96, 1.04 | 0.833 |
| Heart rate | 1.01 | 1.00, 1.01 | 0.072 |
| WBC count | 1.00 | 0.99, 1.01 | 0.648 |
| Hemoglobin | 0.98 | 0.93, 1.04 | 0.536 |
| Platelet | 1.00 | 1.00, 1.00 | 0.554 |
| pH (time dependent) | 0.99 | 0.64, 1.54 | 0.96 |
| PO2 | 1.00 | 1.00, 1.00 | 0.631 |
| PCO2 | 1.01 | 1.00, 1.02 | 0.062 |
| Lactate (time dependent) | 1.03 | 1.01, 1.04 | <0.001 |
| Creatinine | 1.15 | 1.04, 1.26 | 0.004 |
| ^1^HR = Hazard Ratio, CI = Confidence Interval | | | |

## Table S30. Survey-weighted Cox model adjusted with all covariates and IPTW for ICU mortality of cohort 2

| **Characteristic** | **HR^1^** | **95% CI^1^** | **p-value** |
| --- | --- | --- | --- |
| Group |  |  |  |
| Optimal TWA-BG | 1.00 | Reference |  |
| High TWA-BG | 1.38 | 1.01, 1.88 | 0.042 |
| Age | 1.01 | 0.99, 1.02 | 0.299 |
| Gender |  |  |  |
| Female | 1.00 | Reference |  |
| Male | 0.92 | 0.71, 1.20 | 0.546 |
| Weight | 0.99 | 0.99, 1.00 | 0.149 |
| SAPS II (time dependent) | 1.01 | 1.00, 1.01 | <0.001 |
| SOFA score (time dependent) | 1.01 | 0.99, 1.03 | 0.339 |
| Charlson comorbidity index | 1.15 | 1.02, 1.28 | 0.017 |
| CABG |  |  |  |
| YES | 1.00 | Reference |  |
| NO | 3.06 | 1.59, 5.89 | <0.001 |
| PCI |  |  |  |
| YES | 1.00 | Reference |  |
| NO | 0.63 | 0.35, 1.14 | 0.129 |
| IABP |  |  |  |
| YES | 1.00 | Reference |  |
| NO | 1.09 | 0.78, 1.54 | 0.604 |
| PiCCO |  |  |  |
| YES | 1.00 | Reference |  |
| NO | 1.71 | 0.35, 8.35 | 0.51 |
| NICOM (time dependent) |  |  |  |
| YES | 1.00 | Reference |  |
| NO | 1.03 | 0.81, 1.31 | 0.831 |
| Mechanical ventilation (time dependent) |  |  |  |
| YES | 1.00 | Reference |  |
| NO | 0.90 | 0.66, 1.24 | 0.533 |
| Sedative therapy |  |  |  |
| YES | 1.00 | Reference |  |
| NO | 2.17 | 0.77, 6.14 | 0.144 |
| HF (time dependent) |  |  |  |
| YES | 1.00 | Reference |  |
| NO | 1.15 | 1.04, 1.26 | 0.004 |
| Hypertension |  |  |  |
| YES | 1.00 | Reference |  |
| NO | 0.98 | 0.70, 1.38 | 0.925 |
| AFIB |  |  |  |
| YES | 1.00 | Reference |  |
| NO | 1.48 | 1.09, 2.00 | 0.011 |
| T2DM |  |  |  |
| YES | 1.00 | Reference |  |
| NO | 1.18 | 0.90, 1.55 | 0.222 |
| Renal |  |  |  |
| YES | 1.00 | Reference |  |
| NO | 1.48 | 1.00, 2.19 | 0.05 |
| Liver |  |  |  |
| YES | 1.00 | Reference |  |
| NO | 1.70 | 0.90, 3.21 | 0.105 |
| COPD |  |  |  |
| YES | 1.00 | Reference |  |
| NO | 0.96 | 0.69, 1.34 | 0.821 |
| CAD |  |  |  |
| YES | 1.00 | Reference |  |
| NO | 0.94 | 0.70, 1.27 | 0.702 |
| Stroke |  |  |  |
| YES | 1.00 | Reference |  |
| NO | 1.27 | 0.85, 1.92 | 0.246 |
| Malignancy |  |  |  |
| YES | 1.00 | Reference |  |
| NO | 1.43 | 0.95, 2.14 | 0.088 |
| Cardiomyopathy |  |  |  |
| YES | 1.00 | Reference |  |
| NO | 1.24 | 0.89, 1.71 | 0.202 |
| HVD (time dependent) |  |  |  |
| YES | 1.00 | Reference |  |
| NO | 1.08 | 0.99, 1.17 | 0.073 |
| MAP | 0.99 | 0.99, 1.00 | 0.075 |
| Temperature | 0.98 | 0.86, 1.11 | 0.739 |
| Heart rate | 1.01 | 1.00, 1.01 | 0.066 |
| WBC count | 1.00 | 0.99, 1.01 | 0.631 |
| Hemoglobin | 0.98 | 0.93, 1.04 | 0.539 |
| Platelet | 1.00 | 1.00, 1.00 | 0.555 |
| pH (time dependent) | 0.99 | 0.64, 1.54 | 0.971 |
| PO2 | 1.00 | 1.00, 1.00 | 0.645 |
| PCO2 | 1.01 | 1.00, 1.02 | 0.062 |
| Lactate (time dependent) | 1.03 | 1.01, 1.04 | <0.001 |
| Creatinine | 1.15 | 1.04, 1.26 | 0.004 |
| ^1^HR = Hazard Ratio, CI = Confidence Interval | | | |

## Table S31. Multivariate Cox model adjusted with all covariates for in-hospital mortality of original cohort 2

| **Characteristic** | **HR^1^** | **95% CI^1^** | **p-value** |
| --- | --- | --- | --- |
| Group |  |  |  |
| Optimal TWA-BG | 1.00 | Reference |  |
| High TWA-BG | 1.66 | 1.34, 2.06 | <0.001 |
| Age | 1.01 | 1.00, 1.03 | 0.01 |
| Gender |  |  |  |
| Female | 1.00 | Reference |  |
| Male | 0.84 | 0.69, 1.02 | 0.082 |
| Weight | 1.00 | 0.99, 1.00 | 0.671 |
| SAPS II (time dependent) | 1.01 | 1.00, 1.01 | <0.001 |
| SOFA score (time dependent) | 1.01 | 1.00, 1.02 | 0.222 |
| Charlson comorbidity index | 1.06 | 0.99, 1.14 | 0.07 |
| CABG |  |  |  |
| YES | 1.00 | Reference |  |
| NO | 2.32 | 1.36, 3.94 | 0.002 |
| PCI |  |  |  |
| YES | 1.00 | Reference |  |
| NO | 0.97 | 0.61, 1.54 | 0.9 |
| IABP |  |  |  |
| YES | 1.00 | Reference |  |
| NO | 0.95 | 0.74, 1.24 | 0.717 |
| PiCCO |  |  |  |
| YES | 1.00 | Reference |  |
| NO | 0.87 | 0.27, 2.84 | 0.821 |
| NICOM (time dependent) |  |  |  |
| YES | 1.00 | Reference |  |
| NO | 1.12 | 0.97, 1.28 | 0.116 |
| Mechanical ventilation (time dependent) |  |  |  |
| YES | 1.00 | Reference |  |
| NO | 0.95 | 0.81, 1.11 | 0.511 |
| Sedative therapy (time dependent) |  |  |  |
| YES | 1.00 | Reference |  |
| NO | 1.18 | 1.01, 1.39 | 0.036 |
| HF (time dependent) |  |  |  |
| YES | 1.00 | Reference |  |
| NO | 1.16 | 1.08, 1.25 | <0.001 |
| Hypertension (time dependent) |  |  |  |
| YES | 1.00 | Reference |  |
| NO | 1.03 | 0.96, 1.10 | 0.406 |
| AFIB |  |  |  |
| YES | 1.00 | Reference |  |
| NO | 1.13 | 0.90, 1.40 | 0.287 |
| T2DM |  |  |  |
| YES | 1.00 | Reference |  |
| NO | 1.28 | 1.03, 1.60 | 0.026 |
| Renal |  |  |  |
| YES | 1.00 | Reference |  |
| NO | 1.24 | 0.95, 1.63 | 0.119 |
| Liver |  |  |  |
| YES | 1.00 | Reference |  |
| NO | 1.04 | 0.68, 1.60 | 0.856 |
| COPD |  |  |  |
| YES | 1.00 | Reference |  |
| NO | 0.81 | 0.65, 1.02 | 0.079 |
| CAD |  |  |  |
| YES | 1.00 | Reference |  |
| NO | 0.81 | 0.66, 1.00 | 0.048 |
| Stroke (time dependent) |  |  |  |
| YES | 1.00 | Reference |  |
| NO | 1.02 | 0.93, 1.11 | 0.734 |
| Malignancy |  |  |  |
| YES | 1.00 | Reference |  |
| NO | 1.25 | 0.93, 1.70 | 0.141 |
| Cardiomyopathy (time dependent) |  |  |  |
| YES | 1.00 | Reference |  |
| NO | 1.01 | 0.95, 1.08 | 0.695 |
| HVD (time dependent) |  |  |  |
| YES | 1.00 | Reference |  |
| NO | 1.04 | 0.98, 1.10 | 0.191 |
| MAP | 1.00 | 0.99, 1.00 | 0.34 |
| Temperature | 0.95 | 0.86, 1.05 | 0.31 |
| Heart rate | 1.01 | 1.00, 1.01 | 0.026 |
| WBC count | 1.00 | 0.99, 1.01 | 0.89 |
| Hemoglobin | 0.99 | 0.95, 1.03 | 0.526 |
| Platelet | 1.00 | 1.00, 1.00 | 0.769 |
| pH (time dependent) | 0.83 | 0.60, 1.14 | 0.253 |
| PO2 | 1.00 | 1.00, 1.00 | 0.759 |
| PCO2 | 1.00 | 1.00, 1.01 | 0.35 |
| Lactate (time dependent) | 1.01 | 1.00, 1.02 | 0.009 |
| Creatinine (time dependent) | 1.02 | 1.00, 1.04 | 0.046 |
| ^1^HR = Hazard Ratio, CI = Confidence Interval | | | |

## Table S32. Multivariate Cox model adjusted with unbalanced covariates for in-hospital mortality of original cohort 2

| **Characteristic** | **HR^1^** | **95% CI^1^** | **p-value** |
| --- | --- | --- | --- |
| Group (time dependent) |  |  |  |
| Optimal TWA-BG | 1.00 | Reference |  |
| High TWA-BG | 1.15 | 1.08, 1.23 | <0.001 |
| Age | 1.01 | 1.00, 1.02 | 0.012 |
| Weight | 1.00 | 0.99, 1.00 | 0.231 |
| SAPS II (time dependent) | 1.01 | 1.01, 1.01 | <0.001 |
| Charlson comorbidity index | 1.06 | 1.01, 1.12 | 0.031 |
| CABG |  |  |  |
| YES | 1.00 | Reference |  |
| NO | 2.32 | 1.39, 3.87 | 0.001 |
| NICOM (time dependent) |  |  |  |
| YES | 1.00 | Reference |  |
| NO | 1.12 | 0.98, 1.28 | 0.088 |
| HF (time dependent) |  |  |  |
| YES | 1.00 | Reference |  |
| NO | 1.16 | 1.09, 1.24 | <0.001 |
| Hypertension (time dependent) |  |  |  |
| YES | 1.00 | Reference |  |
| NO | 1.03 | 0.96, 1.10 | 0.385 |
| T2DM |  |  |  |
| YES | 1.00 | Reference |  |
| NO | 1.20 | 0.97, 1.48 | 0.098 |
| Renal |  |  |  |
| YES | 1.00 | Reference |  |
| NO | 1.19 | 0.92, 1.53 | 0.184 |
| CAD |  |  |  |
| YES | 1.00 | Reference |  |
| NO | 0.86 | 0.71, 1.05 | 0.138 |
| HVD (time dependent) |  |  |  |
| YES | 1.00 | Reference |  |
| NO | 1.03 | 0.97, 1.09 | 0.395 |
| WBC count | 1.00 | 0.99, 1.01 | 0.637 |
| Platelet | 1.00 | 1.00, 1.00 | 0.288 |
| pH (time dependent) | 0.70 | 0.54, 0.90 | 0.005 |
| PO2 | 1.00 | 1.00, 1.00 | 0.596 |
| Creatinine (time dependent) | 1.02 | 1.00, 1.04 | 0.027 |
| ^1^HR = Hazard Ratio, CI = Confidence Interval | | | |

## Table S33. Multivariate Cox model adjusted with all covariates and IPTW for in-hospital mortality of cohort 2

| **Characteristic** | **HR^1^** | **95% CI^1^** | **p-value** |
| --- | --- | --- | --- |
| Group |  |  |  |
| Optimal TWA-BG | 1.00 | Reference |  |
| High TWA-BG | 1.32 | 1.02, 1.71 | 0.038 |
| Age | 1.00 | 0.99, 1.02 | 0.615 |
| Gender |  |  |  |
| Female | 1.00 | Reference |  |
| Male | 0.81 | 0.65, 1.02 | 0.075 |
| Weight | 1.00 | 0.99, 1.00 | 0.271 |
| SAPS II (time dependent) | 1.01 | 1.01, 1.01 | <0.001 |
| SOFA score (time dependent) | 1.01 | 0.99, 1.02 | 0.419 |
| Charlson comorbidity index | 1.15 | 1.04, 1.27 | 0.006 |
| CABG |  |  |  |
| YES | 1.00 | Reference |  |
| NO | 2.82 | 1.54, 5.14 | <0.001 |
| PCI |  |  |  |
| YES | 1.00 | Reference |  |
| NO | 0.74 | 0.43, 1.29 | 0.291 |
| IABP |  |  |  |
| YES | 1.00 | Reference |  |
| NO | 1.14 | 0.84, 1.55 | 0.402 |
| PiCCO |  |  |  |
| YES | 1.00 | Reference |  |
| NO | 1.18 | 0.30, 4.64 | 0.812 |
| NICOM (time dependent) |  |  |  |
| YES | 1.00 | Reference |  |
| NO | 1.03 | 0.84, 1.26 | 0.773 |
| Mechanical ventilation (time dependent) |  |  |  |
| YES | 1.00 | Reference |  |
| NO | 1.02 | 0.79, 1.32 | 0.867 |
| Sedative therapy (time dependent) |  |  |  |
| YES | 1.00 | Reference |  |
| NO | 1.13 | 0.88, 1.45 | 0.352 |
| HF (time dependent) |  |  |  |
| YES | 1.00 | Reference |  |
| NO | 1.16 | 1.07, 1.26 | <0.001 |
| Hypertension (time dependent) |  |  |  |
| YES | 1.00 | Reference |  |
| NO | 1.01 | 0.93, 1.10 | 0.771 |
| AFIB |  |  |  |
| YES | 1.00 | Reference |  |
| NO | 1.19 | 0.91, 1.55 | 0.202 |
| T2DM |  |  |  |
| YES | 1.00 | Reference |  |
| NO | 1.18 | 0.93, 1.49 | 0.176 |
| Renal |  |  |  |
| YES | 1.00 | Reference |  |
| NO | 1.47 | 1.05, 2.06 | 0.025 |
| Liver |  |  |  |
| YES | 1.00 | Reference |  |
| NO | 1.49 | 0.85, 2.60 | 0.165 |
| COPD |  |  |  |
| YES | 1.00 | Reference |  |
| NO | 0.89 | 0.67, 1.19 | 0.433 |
| CAD |  |  |  |
| YES | 1.00 | Reference |  |
| NO | 0.88 | 0.67, 1.15 | 0.34 |
| Stroke (time dependent) |  |  |  |
| YES | 1.00 | Reference |  |
| NO | 1.02 | 0.92, 1.14 | 0.663 |
| Malignancy |  |  |  |
| YES | 1.00 | Reference |  |
| NO | 1.26 | 0.92, 1.73 | 0.153 |
| Cardiomyopathy (time dependent) |  |  |  |
| YES | 1.00 | Reference |  |
| NO | 1.04 | 0.96, 1.12 | 0.353 |
| HVD (time dependent) |  |  |  |
| YES | 1.00 | Reference |  |
| NO | 1.04 | 0.97, 1.11 | 0.306 |
| MAP | 0.99 | 0.99, 1.00 | 0.052 |
| Temperature | 0.95 | 0.85, 1.07 | 0.435 |
| Heart rate | 1.00 | 1.00, 1.01 | 0.061 |
| WBC count | 1.00 | 0.99, 1.01 | 0.845 |
| Hemoglobin | 0.99 | 0.94, 1.04 | 0.6 |
| Platelet | 1.00 | 1.00, 1.00 | 0.775 |
| pH (time dependent) | 1.03 | 0.72, 1.47 | 0.876 |
| PO2 (time dependent) | 1.00 | 1.00, 1.00 | 0.747 |
| PCO2 | 1.01 | 1.00, 1.02 | 0.053 |
| Lactate (time dependent) | 1.02 | 1.01, 1.03 | 0.004 |
| Creatinine (time dependent) | 1.03 | 1.01, 1.05 | 0.005 |
| ^1^HR = Hazard Ratio, CI = Confidence Interval | | | |

## Table S34. Survey-weighted Cox model adjusted with all covariates and IPTW for in-hospital mortality of cohort 2

| **Characteristic** | **HR^1^** | **95% CI^1^** | **p-value** |
| --- | --- | --- | --- |
| Group (time dependent) |  |  |  |
| Optimal TWA-BG | 1.00 | Reference |  |
| High TWA-BG | 1.08 | 1.00, 1.17 | 0.042 |
| Age | 1.00 | 0.99, 1.02 | 0.622 |
| Gender |  |  |  |
| Female | 1.00 | Reference |  |
| Male | 0.81 | 0.65, 1.02 | 0.074 |
| Weight | 1.00 | 0.99, 1.00 | 0.274 |
| SAPS II (time dependent) | 1.01 | 1.01, 1.01 | <0.001 |
| SOFA score (time dependent) | 1.01 | 0.99, 1.02 | 0.429 |
| Charlson comorbidity index | 1.15 | 1.04, 1.27 | 0.006 |
| CABG |  |  |  |
| YES | 1.00 | Reference |  |
| NO | 2.81 | 1.54, 5.12 | <0.001 |
| PCI |  |  |  |
| YES | 1.00 | Reference |  |
| NO | 0.74 | 0.43, 1.28 | 0.283 |
| IABP |  |  |  |
| YES | 1.00 | Reference |  |
| NO | 1.14 | 0.84, 1.55 | 0.394 |
| PiCCO |  |  |  |
| YES | 1.00 | Reference |  |
| NO | 1.17 | 0.30, 4.60 | 0.822 |
| NICOM (time dependent) |  |  |  |
| YES | 1.00 | Reference |  |
| NO | 1.03 | 0.84, 1.26 | 0.76 |
| Mechanical ventilation (time dependent) |  |  |  |
| YES | 1.00 | Reference |  |
| NO | 1.02 | 0.79, 1.32 | 0.873 |
| Sedative therapy (time dependent) |  |  |  |
| YES | 1.00 | Reference |  |
| NO | 1.13 | 0.88, 1.45 | 0.353 |
| HF (time dependent) |  |  |  |
| YES | 1.00 | Reference |  |
| NO | 1.16 | 1.07, 1.26 | <0.001 |
| Hypertension (time dependent) |  |  |  |
| YES | 1.00 | Reference |  |
| NO | 1.01 | 0.93, 1.10 | 0.783 |
| AFIB |  |  |  |
| YES | 1.00 | Reference |  |
| NO | 1.19 | 0.91, 1.55 | 0.206 |
| T2DM |  |  |  |
| YES | 1.00 | Reference |  |
| NO | 1.18 | 0.93, 1.49 | 0.177 |
| Renal |  |  |  |
| YES | 1.00 | Reference |  |
| NO | 1.47 | 1.05, 2.06 | 0.024 |
| Liver |  |  |  |
| YES | 1.00 | Reference |  |
| NO | 1.49 | 0.85, 2.60 | 0.166 |
| COPD |  |  |  |
| YES | 1.00 | Reference |  |
| NO | 0.89 | 0.67, 1.19 | 0.434 |
| CAD |  |  |  |
| YES | 1.00 | Reference |  |
| NO | 0.88 | 0.67, 1.15 | 0.335 |
| Stroke (time dependent) |  |  |  |
| YES | 1.00 | Reference |  |
| NO | 1.02 | 0.92, 1.14 | 0.662 |
| Malignancy |  |  |  |
| YES | 1.00 | Reference |  |
| NO | 1.26 | 0.92, 1.73 | 0.154 |
| Cardiomyopathy (time dependent) |  |  |  |
| YES | 1.00 | Reference |  |
| NO | 1.04 | 0.96, 1.12 | 0.342 |
| HVD (time dependent) |  |  |  |
| YES | 1.00 | Reference |  |
| NO | 1.04 | 0.97, 1.11 | 0.321 |
| MAP | 0.99 | 0.99, 1.00 | 0.052 |
| Temperature | 0.95 | 0.85, 1.07 | 0.423 |
| Heart rate | 1.00 | 1.00, 1.01 | 0.062 |
| WBC count | 1.00 | 0.99, 1.01 | 0.85 |
| Hemoglobin | 0.99 | 0.94, 1.04 | 0.585 |
| Platelet | 1.00 | 1.00, 1.00 | 0.752 |
| pH (time dependent) | 1.03 | 0.72, 1.47 | 0.854 |
| PO2 | 1.00 | 1.00, 1.00 | 0.892 |
| PCO2 | 1.01 | 1.00, 1.02 | 0.054 |
| Lactate (time dependent) | 1.02 | 1.01, 1.03 | 0.004 |
| Creatinine (time dependent) | 1.03 | 1.01, 1.05 | 0.005 |
| ^1^HR = Hazard Ratio, CI = Confidence Interval | | | |
